# The Benefits and Harms of Screening for Prostate Cancer in Adults Aged 18 Years and Older: A Systematic Review

**DOI:** 10.3390/curroncol33040199

**Published:** 2026-03-31

**Authors:** Alexandria Bennett, Niyati Vyas, Nicole Shaver, Faris Almoli, Taddele Kibret, Andrew Loblaw, Lisa Del Giudice, Xiaomei Yao, Becky Skidmore, Melissa Brouwers, Julian Little, David Moher

**Affiliations:** 1 School of Epidemiology and Public Health, Faculty of Medicine, University of Ottawa, Ottawa, ON K1G 5Z3, Canada; d.bennett@uottawa.ca (A.B.); nicole.shaver@uottawa.ca (N.S.); almoli.faris@gmail.com (F.A.); tkibr005@uottawa.ca (T.K.); melissa.brouwers@uottawa.ca (M.B.); jlittle@uottawa.ca (J.L.); dmoher@ohri.ca (D.M.); 2 Evaluative Clinical Sciences, Odette Cancer Research Program, Sunnybrook Research Institute, University of Toronto, Toronto, ON M4N 3M5, Canada; andrew.loblaw@sunnybrook.ca; 3 Department of Family and Community Medicine, Sunnybrook Health Sciences Centre, University of Toronto, Toronto, ON M4N 3M5, Canada; lisa.delgiudice@sunnybrook.ca; 4 Department of Health Research Methods, Evidence, and Impact, McMaster University, Hamilton, ON L8N 3Z5, Canada; yaoxia@mcmaster.ca; 5 Department of Oncology, McMaster University, Hamilton, ON L8V5C2, Canada; 6 Independent Information Specialist, Ottawa, ON K1T 3Z2, Canada; becky.skidmore.rls@gmail.com; 7 Clinical Epidemiology Program, Ottawa Hospital Research Institute, Ottawa, ON K1H 8L6, Canada

**Keywords:** prostate cancer, screening, prostate-specific antigen, MRI screening, primary care, systematic review, guideline

## Abstract

Given ongoing uncertainty about the benefits and harms of prostate-specific antigen (PSA) screening, this systematic review updates the evidence to inform guideline recommendations for adults aged ≥ 18 years in primary care. We searched multiple bibliographic databases from inception to 30 May 2022, with an update on 24 July 2024, for randomized controlled trials (RCTs) and comparative observational studies evaluating PSA-based screening with or without adjunctive technologies such as magnetic resonance imaging (MRI). Studies were selected in duplicate, with data extraction and quality assessment verified by a second reviewer; risk of bias and evidence certainty were assessed using study design-specific tools and GRADE. Four RCTs and one cohort study (17 articles) were included: ERSPC, PLCO and CAP compared PSA screening with no screening, while STHLM3-MRI evaluated a risk-based test combined with MRI targeted biopsy. Meta-analysis showed 0.96 fewer prostate cancer deaths per 1000 individuals invited to screen, corresponding to a 12% relative reduction over 9.5–22 years (RR 0.88, 95% CI 0.81–0.95). One trial estimated 2.3% to 10.3% overdiagnosis over 10–14 years. Overall certainty of evidence was low or very low. PSA screening may offer a small mortality benefit, but uncertainty and variable harms limit confidence, underscoring the need for high-quality evidence, particularly for MRI and risk-based screening strategies.

## Simple Summary

With ongoing debate surrounding the benefits and harms of prostate-specific antigen (PSA) screening test for prostate cancer, we conducted this review to gather evidence that could help inform prostate cancer screening guideline recommendations for adults aged 18 years and older. We analyzed results from four large randomized clinical trials and one cohort study that compared PSA screening with no screening, including one study that evaluated risk-based testing with magnetic resonance imaging (MRI). Overall, PSA screening was associated with about one fewer prostate cancer death per 1000 people invited for screening over 9.5–22 years, suggesting a small potential benefit. However, one trial also observed overdiagnosis, and the overall quality of the evidence across all outcomes was low, limiting the confidence in these findings. Further high-quality research is recommended, particularly on new MRI and risk-based screening strategies.

## 1. Introduction

Prostate cancer is the most common cancer among individuals with a prostate in 112 countries, representing 15% of all cancer cases. A report by the Lancet Commission projects that the number of cases will rise to 2.9 million by 2040, highlighting the need for urgent strategies and recommendations to effectively address this issue [1]. There are several key risk factors for prostate cancer, including family history, with an estimated 20% of cases linked to familial connections [2,3]. This is largely due to the interaction of multiple shared susceptibility genes of moderate to low penetrance, shared environmental exposures, or potential biases [4]. Familial clustering may be partly explained by genetic predisposition and recent segregation analyses that suggest an autosomal dominant gene could contribute to this inherited risk [5]. Shared environmental factors also contribute to familial risk [2,6]. Genome-wide association studies have identified over 400 single nucleotide polymorphisms associated with increased susceptibility to prostate cancer outcomes [2,6,7,8]. Germline mutations in breast cancer predisposition genes significantly influence an individual’s susceptibility to carcinogenesis [9]. Individuals with a prostate carrying a BRCA1 mutation have a standardized incidence ratio (SIR) of 2.35 for prostate cancer compared to the general population, while those with a BRCA2 mutation exhibit a higher SIR of 4.45 [9]. Additionally, BRCA2 mutations raises the lifetime risk of developing prostate cancer by about 20%, whereas BRCA1 mutations by about 9.5%, both notably higher compared to non-carriers [6,9]. Race and ethnicity influence risk, with Black individuals, particularly those in the Caribbean and sub-Saharan Africa, experiencing the highest prevalence and mortality rates, while differences in diagnostic practices and socioeconomic factors may further impact access to the quality of care [2,3,7,10,11,12]. Moreover, there is inconsistency in the evidence regarding other risk factors, such as alcohol consumption [2], tobacco smoking [13,14], dietary habits [2,15], excess weight [16], and industrial or occupational exposures [15,17]. Additionally, while benign prostatic hyperplasia (BPH) has been associated with an increased risk of prostate cancer, evidence on a causal relationship remains mixed, with some studies supporting a direct link [18,19] and others suggesting shared risk factors or detection bias [20,21,22].

PSA testing measures the total concentration of PSA in the bloodstream and is the most common method for screening for prostate cancer [23]. While there are no universally recommended cutoff values for PSA levels, some guidelines suggest thresholds based on age and race in the screening context [24,25]. Elevated PSA levels can result from non-cancerous conditions as well, including benign prostatic hyperplasia or urinary tract infections [23,26,27]. Though widely used for diagnosing and managing prostate cancer, PSA testing has uncertainties and conflicting guidance regarding its benefits and potential harms. It has been suggested that PSA levels should be monitored over time and considered with other factors like symptoms and prostate size [28]. The Digital Rectal Examination (DRE) is another method used in conjunction with the PSA test in clinical practice [29]. Although it is commonly understood that PSA combined with DRE improves effectiveness for prostate cancer screening, while DRE alone is often viewed as inadequate [30], there remains variability in practice and uncertainty about the value of each modality. A Canadian survey also highlighted that using DRE as a standalone screening tool is not feasible due to insufficient physician training [31].

Prostate cancer screening may be beneficial in reducing morbidity and mortality through early detection, especially at treatable stages. While results from the extended follow-ups of a large European randomized trial [32] and a systematic review [33] indicate that PSA-based screening may lower prostate cancer mortality, this benefit is not consistently supported across trials, including conflicting evidence from the US Prostate, Lung, Colorectal, and Ovary trial [34]. Additionally, results from one study also suggests that any potential gains in life expectancy from screening may be offset by reductions in quality of life [35]. One significant concern related to the harms of screening for prostate cancer is overdiagnosis [35,36,37]. Overdiagnosis can lead to overtreatment and result in treatment-related side effects such as urinary incontinence, erectile dysfunction, and bowel disturbances, ultimately affecting the overall quality of life [38], although these symptoms are largely mitigated by the appropriate use of active surveillance in favourable risk patients [39]. Additionally, the performance of the PSA test can vary depending on the threshold used, and it may not be adequate to use PSA testing alone in detecting clinically significant prostate cancer. Its effectiveness can be enhanced by utilizing additional assessment methods such as risk calculators [40], multiparametric MRI (mpMRI) [41], or genetic information. Incorporating genetic markers into PSA screening has also been proposed to enable more personalized approaches [42]. Recent genome-wide association studies suggest that genetic data can improve understanding of PSA variation across ancestry groups [43]. While some studies found limited benefit in combining genetic information with PSA for risk discrimination [44], others showed that genetically adjusted PSA levels may enhance detection of aggressive prostate cancer [45]. Polygenic scores and emerging at-home saliva-based genetic tests further highlight the potential for personalized screening strategies [46].

Despite the substantial burden of prostate cancer, the role of routine screening remains the subject of debate due to the complex trade-offs between the potential benefits and harms. The Canadian Task Force on Preventive Health Care released guidance in 2014 and recommended against PSA screening for individuals with a prostate, citing insufficient evidence of mortality reduction and potential harms such as false positives and overdiagnosis [47]. Similarly, the American College of Physicians (2015) [48], the American Academy of Family Physicians (2019) [49], the U.S. Preventive Services Task Force (2018) [25,50], and the UK National Screening Committee (2020) [51] also advised against screening for men aged 50 to 69, due to unclear effectiveness and associated risks. More recently, in November 2025, the UK National Screening Committee released a draft recommendation followed by a public consultation period, and updated guidance is awaited [52]. Other groups, including the BMJ Rapid Recommendations Panel (2018) [53] and the National Comprehensive Cancer Network [54], have supported shared decision-making in patient care. The European, Canadian, and American Association of Urology advocates for screening and counselling before PSA testing and encourages an individualized approach to early detection [55,56,57]. A recent study by the International Agency for Research on Cancer (IARC) also noted a significant variability in prostate cancer incidence across Europe, suggesting overdiagnosis and warning against the population-level impacts of unregulated PSA testing [58]. Emerging global initiatives, such as the Prostate Cancer Awareness and Screening Initiative for the European Union (PRAISE-U) led by the IARC, aim to provide concrete evidence for a risk-stratified approach to the early detection of prostate cancer [59]. These differences across guidelines, such as variation in screening age thresholds, risk stratification criteria, and use of tools like MRI or genetic testing, emphasize persistent clinical uncertainty, specifically regarding risk-adapted approaches based on earlier trials, and underscore the need for this review.

Although newer screening tools such as multiparametric MRI and risk-based algorithms have emerged in recent years and may shift the benefit–harm balance of PSA-screening, current guidelines vary in their adoption and interpretation of these advances. Many lack consensus on how best to integrate such tools in routine practice, reflecting an ongoing gap between evolving evidence and clinical recommendations. Therefore, this systematic review aims to evaluate the benefits and harms of PSA screening, both as a standalone method and in combination with other approaches such as risk stratification and MRI to ensure that recommendations reflect up-to-date advances and are evidence-based.

Building on the identified gaps and advances in prostate cancer screening, this review addresses two key questions (KQs). The first key question (KQ1) is “what are the benefits and harms of prostate cancer screening?”, as well as the sub-questions: (a) do the benefits and harms differ by initial screening modalities (i.e., PSA alone, DRE alone, or PSA+DRE)?; (b) in screening with PSA, do benefits and harms differ by PSA threshold value?; (c) do the benefits and harms differ by age group (<55 years, 55–69 years, ≥70 years)?; (d) do the benefits and harms differ by other risk factors such as race/ethnicity and/or family history? The second key question (KQ2) is “what are the benefits and harms of incorporating additional information (e.g., risk stratification, MRI) into clinical decision-making following an elevated PSA test?”

## 2. Materials and Methods

### 2.1. Protocol and Registration

This review was conducted and reported in accordance with the Preferred Reporting Items for Systematic Reviews and Meta-Analyses (PRISMA) 2020 checklist (Supplementary File S1) and following guidance from the Cochrane Handbook and the Grading of Recommendations Assessment, Development and Evaluation (GRADE) working group [60,61]. Details on protocol development, eligibility criteria, and outcome determination are available in the registered protocol [62] with PROSPERO (CRD42022314407) and accessible on the Open Science Framework (https://osf.io/dm32k/).

### 2.2. Eligibility Criteria

The detailed inclusion and exclusion criteria for KQ1 and KQ2 are presented in Table 1, organized according to the PICOTS framework and supplemented with additional information on eligible settings, study designs, and publication languages. For KQ1, this review included individuals aged 18 years or older who were not identified as having an increased risk for prostate cancer. Eligible screening interventions included a group with clinical or laboratory tests, including PSA alone, DRE alone, and PSA with DRE before biopsy. Eligible comparators included no prostate screening, usual care, or an alternate type of screening (i.e., DRE alone) for individuals. We considered studies conducted in primary-healthcare-related settings and included randomized controlled trials (RCTs), cluster RCTs, quasi-randomized trials, and cohort studies published from 2019 up to the date of the literature search and from 2012 onward for non-randomized trials (nRCTs). Outcomes reflecting the potential benefits of prostate cancer screening included a reduction in prostate cancer-related mortality, all-cause mortality, and incidence of metastatic cancer. Eligible harms outcomes were increased false positives, overdiagnosis, complications due to biopsy, urinary or bowel incontinence, and erectile dysfunction. Health-related quality of life and psychological effects were considered as either potential benefits or harms depending on the direction of effect.

For KQ2, we included individuals aged 18 years or older with an elevated PSA test as described in Table 1. Eligible interventions comprised strategies incorporating groups offered with additional tests, such as risk stratification tools or MRI—used alone, sequentially, or in combination to inform decisions regarding prostate biopsy. The comparator group included no additional testing (i.e., PSA-based screening only) or usual care. Studies conducted in primary care or primary-healthcare-related settings were eligible. We included RCTs, cluster RCTs, and cohort studies that met our predefined eligibility criteria. Outcomes representing potential benefits included a reduction in prostate cancer-related mortality, all-cause mortality, and incidence of metastatic cancer. Potential harms encompassed increased false positive results, overdiagnosis, biopsy-related complications, urinary or bowel incontinence, and erectile dysfunction. Health-related quality of life and psychological effects were evaluated as either potential benefits or harms, depending on their direction and clinical relevance.

### 2.3. Data Sources and Search Strategy

The search strategies were designed and refined through an iterative process by an experienced medical information specialist in collaboration with the review team. Prior to implementation, the MEDLINE search strategy underwent peer review by a second senior information specialist using the PRESS Checklist (Supplementary File S2) [63]. The complete electronic search strategies are provided in Supplementary File S3.

Database searching was conducted in Ovid MEDLINE^®^ ALL, Embase Classic+Embase, and EBM Reviews—Cochrane Central Register of Controlled Trials (CENTRAL) using Ovid’s multifile search function, with duplicate records removed via the platform’s deduplication tool. Searches for RCTs were performed in all three databases. The strategies combined controlled vocabulary terms like “Prostatic Neoplasms”, “Mass Screening”, “Digital Rectal Examination” with relevant keywords, i.e., “prostate cancer”, “screen”, “TRUS”. An adapted version of the 2008 Cochrane highly sensitive search strategy, (i.e., sensitivity- and precision-maximizing version) was applied to identify RCTs in MEDLINE and Embase, while no methodological filter was used in CENTRAL. In addition, searches for nRCTs were conducted in MEDLINE and Embase. To update the 2020 UK NSC report [51] and to capture RCTs, the search was restricted to studies published from 20 August 2019 onward. To capture nRCTs, we updated the search strategy from the 2013 Cochrane review [64] and therefore limited our search from June 2012. There were no language restrictions on any of the searches, but, when possible, animal-only records, opinion pieces and case studies were removed from the results. We also removed conference abstracts from Embase and CENTRAL. All searches were performed on 30 May 2022 and updated on 24 July 2024. Results were downloaded and deduplicated using EndNote version 9.3.3 (Clarivate) and uploaded to DistillerSR (version 2.35) [65]. To identify relevant grey literature for the review, a targeted website search was conducted on organizations with a focus on prostate cancer, pathology, urology, general practice, and healthcare quality. The search strategy and the grey literature process are outlined in Supplementary Files S3 and S4.

### 2.4. Study Selection

Screening was completed by two independent reviewers in two phases: (1) title and abstract screening and (2) full-text screening. After deduplicating the search results, studies were uploaded to DistillerSR, a reference management and systematic review software. Before starting the screening process, the review team conducted a pilot exercise using a random sample of 50 references for titles and abstracts and 25 randomly selected references for full-text review. Screening commenced when there was a 95% agreement during the pilot among reviewers based on the pre-specified inclusion and exclusion criteria for each specific KQ. Any discrepancies among the reviewers were resolved following a discussion or consultation with a third reviewer.

### 2.5. Data Extraction

A group of reviewers completed the data extraction process using Microsoft Excel. This process involved one reviewer independently extracting data from included studies, while another reviewer verified the extracted information. Any discrepancies identified during the verification process were resolved either through consensus among reviewers or by consulting a third reviewer. Details regarding the preliminary data items and the data extraction form can be found in Supplementary File S5. This information includes study publication details, intervention and comparator groups, as well as outcome data for both groups. Additionally, data were extracted from included studies on the sample size, the adjusted and unadjusted effect measures, and the reported limitations of the studies.

### 2.6. Risk-of-Bias Assessments

Two reviewers assessed the risk of bias for each included study, distributing the studies equally between them. After this initial assessment, another reviewer verified their evaluations. The Cochrane risk of bias tool 2.0 was utilized for the quality appraisal of RCTs [66]. In contrast, the relevant study design checklist from the Joanna Briggs Institute (JBI) was used to assess the methodological quality of non-randomized or observational studies [67].

To remain consistent with our previous review methods, the overall risk judgement of a trial based on the Cochrane risk of bias 2.0 tool was determined as follows: if all domains in the tool were assessed as ‘low risk’, then the overall judgement is also classified as ‘low risk’. Conversely, if any critical domain is rated as having a ‘high risk’, the overall risk of bias will be judged as ‘high risk’. Additionally, trials may be classified as having ‘some concerns’ if there are multiple unclear domains. The results of the risk-of-bias assessments were summarized narratively and visually in a table. Since the JBI checklists do not provide a recommended scoring system for overall study quality, we implemented a scale-based approach to tally quality scores for each item, where zero indicates unclear or no answers and one signifies that the item was met.

### 2.7. Data Analysis

The study characteristics, risk of bias, and the methodological quality of included studies are presented both in tables and through a narrative summary. Whenever possible, we calculated and presented relative and absolute effects with 95% confidence intervals in the GRADE summary of findings and evidence profile tables. For dichotomous outcomes, we reported effect sizes using relative risk (RR), hazard ratio (HR), and risk difference (RD). For any continuous outcomes, we utilized the mean difference (MD) as the effect estimate.

We prioritized data from the longest available follow-up period, as evidence indicates that extended follow-up after randomization may yield more robust estimates of the impact of screening on prostate cancer mortality and metastasis in clinical trials, though we acknowledge that very long follow-ups may yield diminishing returns due to dilution of the randomized contrast through contamination and screening cessation [68]. However, all reported follow-up durations were presented in the summary of findings table for each outcome to ensure transparency.

### 2.8. Summary of Key Findings

Results were evaluated separately for each study outcome according to relevant comparators and are presented in a GRADE Summary of Findings Table. This table provides comprehensive details on the interventions and comparators, sample size, the number of included studies included in the analysis, both relative and absolute effects, assessments of evidence certainty, and the overall strength of evidence. As decided a priori, we reported the relative risk or odds ratio along with the corresponding 95% confidence intervals by following the GRADE guidelines for calculations. In instances of continuous outcomes, where different measurement tools were used to assess outcomes, we reported the mean difference along with the 95% confidence intervals. In accordance with GRADE guidelines for estimating absolute effects indirectly, these were derived by applying the pooled relative effect estimates for each outcome to the baseline risk from the comparator group. When multiple studies contributed baseline risks, these were averaged across studies before calculating the absolute effects.

### 2.9. Data Synthesis

We a priori decided to present the results from RCTs and observational studies separately, given the inherent differences in study design. Prior to conducting a meta-analysis, we summarized the characteristics of each included study and compared key elements of the PICOTS framework to identify potential sources of clinical and methodological heterogeneity across study designs. Statistical heterogeneity was evaluated using the I^2^ statistic and Cochran’s Q test, with a significance threshold offset at *p*-value <0.10. For the I^2^ statistics, heterogeneity was classified as low (0–25%), moderate (26–50%), substantial (51–75%), or considerable (>75%). In case of detecting the considerable heterogeneity (>75%), pooling of results was considered only if the heterogeneity could be explained through sample size and subgroup analyses.

Where appropriate, study results were pooled using the RevMan software (version 5.3) [69], with the DerSimonian and Laird random-effects model. Forest plots were generated to visually display the range of effect estimates from the included studies. For outcomes where pooling was not feasible, a narrative synthesis was conducted. Additionally, pooled estimates were also used to support the assessment the domains of inconsistency and imprecision when rating the evidence certainty according to GRADE.

### 2.10. Subgroup and Sensitivity Analysis

Based on an a priori protocol, we planned subgroup analyses by age group (including those under 55 years), number of screening rounds attended, PSA level, ethnicity, obesity, and family history of prostate cancer, as reported in detail by the study authors. Additionally, we planned sensitivity analyses to assess the robustness and reliability of our review findings. The parameters of interest included restricting the analysis to studies of low or moderate overall risk of bias, different publication types (i.e., removing abstracts only or pre-prints), or issues as considered in the risk of bias tools.

### 2.11. Certainty of the Evidence

We assessed the overall certainty of evidence for each outcome using the GRADE framework. Five domains were assessed: risk of bias, inconsistency, indirectness, imprecision, and risk of publication bias. These domains helped determine the overall rating of evidence certainty. Each outcome was rated as very low, low, moderate, or high certainty of evidence. All RCTs were initially graded as having high certainty of evidence and were subsequently downgraded based on the ratings of each individual domain. Conversely, all observational studies were initially rated as having low certainty of evidence and were downgraded for issues in the domains mentioned above or, where applicable, upgraded based on the evaluation of large effect sizes, dose-response gradients, or the effect of plausible confounding.

We determined risk of bias ratings for each trial. To evaluate inconsistency, we examined whether effect sizes varied across studies by analyzing heterogeneity. In instances where a meta-analysis was conducted, we assessed heterogeneity using the I^2^ statistic along with other criteria, such as the spreading of point estimates, the confidence intervals overlap, and the relevance of statistical tests (i.e., low *p*-values). Imprecision was assessed using a minimally contextualized approach. For binary outcomes, we evaluated 95% confidence intervals to determine whether they included or excluded the null effect (i.e., a relative risk of 1.0) [70]. Additionally, in the absence of a predefined clinical threshold, we applied the GRADE working group’s suggested rule for an appreciable benefit and harm that warrants rating down if a relative risk reduction (RRR) or a RR increase of 25% or more [70]. Confidence intervals that included or crossed the null effect were rated down for imprecision. For continuous outcomes, we rated imprecision considering the optimal information size (OIS), which considers the sample size required to detect an important difference, using conventional thresholds for type 1 (α = 0.05), type II error (β = 0.2) and an appropriate population standard deviation based on one of the relevant studies [70]. In assessing indirectness, we considered the generalizability of the evidence for a particular outcome concerning our PICO criteria. We have included footnotes providing details related to the ratings of each domain and our rationale in all the GRADE and summary of findings table.

## 3. Results

### 3.1. Protocol Deviations

Due to resource constraints, we did not complete KQ3 or KQ4, as outlined in the original protocol. For KQ2, a de novo review was not performed as the search strategy was deemed similar enough to that of KQ1 to perform one search strategy for both questions. To expedite the screening process during the search update, we employed DAISY Rank, a machine learning prioritization tool within DistillerSR to rank references by relevance using the highest rank score (between 0 and 1), which indicates how likely a reference is to be included, with scores closer to 1 indicating a higher likelihood of inclusion. We chose a remaining score of 0.1 to exclude remaining references. We performed a quality check using DistillerAI to review references that were screened manually and by AI. For the quality assessment of observational studies, we used the JBI critical appraisal tool instead of the ROBINS-I tool to ensure consistency across projects.

### 3.2. Results of the Search

**Figure 1:**
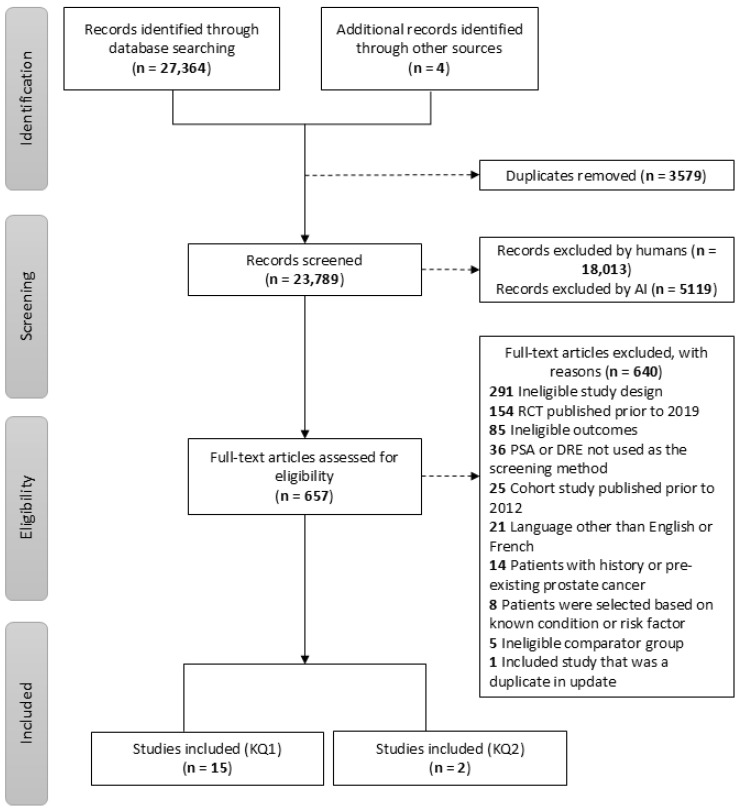
PRISMA flow diagram.

A total of 27,364 records were identified through our electronic search, and an additional four records were identified through our grey literature search. Following deduplication, 23,789 titles and abstracts were reviewed, of which 18,013 were excluded by human reviewers and 5119 were excluded by AI screening. A total of 657 full-text studies were reviewed by human reviewers, of which 640 were excluded. In total, 17 studies met our eligibility criteria and were included in this review. The PRISMA diagram (Figure 1) below provides details on the search results, including reasons for exclusion. The full list of excluded studies with reasons for exclusion is available in Supplementary File S6.

### 3.3. Study Characteristics

Detailed information on the study characteristics of included studies is outlined in Supplementary File S7. For KQ1, we included 15 articles from three randomized controlled trials [32,71,72,73,74,75,76,77,78,79,80,81,82,83] and one cohort study [84]. The RCTs consisted of the European Randomized Study of Screening for Prostate Cancer (ERSPC), the US Prostate, Lung, Colorectal, and Ovary (PLCO) trial, and the Cluster Randomized Trial of PSA testing for Prostate Cancer (CAP), all of which were also incorporated in the 2020 UK NSC report [51] and have additional years of follow-up. The ERSPC trial included eight trial centers across multiple countries, with differences in PSA thresholds, screening intervals, and recruitment strategies. Belgium and Netherlands joined ERSPC in 1991 and 1994. Six countries (Finland, Italy, Sweden, Spain, Portugal and Switzerland) joined in 1995–1996, and France joined in 2002; however, Portugal was excluded due to issues with data. The ERSPC targeted men aged 50–74 years and typically screened every 4 years, except in Sweden, where it was every 2 years. Most centers used a PSA cutoff value of 3.0 ng/mL as an indication for biopsy, except in Finland, where a cutoff of 4.0 ng/mL was applied. The CAP trial was performed in the United Kingdom and randomized men aged 50–69 years for a single screening test, with a PSA cutoff value for biopsy set to 3.0 ng/ml. In contrast, the PLCO trial randomized American men aged 55–74 years with a one-year screening interval, using a PSA cutoff value of 3.0 ng/mL to guide biopsy recommendations; however, biopsy was not mandated. The cohort study used data from the Surveillance, Epidemiology and End Results (SEER) database in the United States.

Two articles evaluated the STHLM3-MRI trial conducted between 2018 and 2020, and were relevant for KQ2 [85,86]. This trial involved men aged 50–74 years using a combined screening approach (i.e., PSA screening and the risk prediction model (Stockholm3 test)) followed by a random assignment of individuals to either the experimental biopsy group or the standard biopsy group.

### 3.4. Risk of Bias

Overall, we had some concerns or high concerns of bias in the ERSPC, PLCO, CAP, and STHLM3-MRI trials. The Finnish and French arm of the ERSPC trial received a high-risk rating due to insufficient information on baseline characteristics, which complicated the evaluation of the randomization process, low participation rates, and high rates of contamination from PSA testing in the control arm [72,73,74,77,78]. The Dutch, Spanish, and Swedish arms of the ERSPC trial were rated with some concerns of bias due to contamination in the control arm [71,75,76,80]. The PLCO trial was rated high due to contamination in the control group (more men had PSA screening in the control than in the experimental arm) [87], while the CAP trial raised concerns primarily related to contamination despite its cluster-randomized design [79,81]. The STHLM3-MRI trial was rated with a high risk of bias due to lack of information on allocation concealment, concerns with participant and clinician awareness of the intervention, and risk of contamination within the control arm [85,86]. The single included cohort study was rated with a high risk of bias due to inadequate adjustment of confounding variables and differences between groups based on predicted data, along with an insufficient timeframe for outcomes [84]. Detailed risk-of-bias assessments are available in Supplementary File S8.

### 3.5. Pooling and Subgroup Analyses

Where available, results are presented by age group, number of screening rounds attended, and PSA level under our findings. Forest plots were used to visualize meta-analyses (Supplementary File S9). We were unable to perform planned subgroup analyses for those under 55 years of age, ethnicity, obesity, and family history due to lack of data reported for these factors.

### 3.6. Findings

The findings for RCTs and cohort studies are presented below. We did not find any studies that reported on outcomes on erectile dysfunction, incontinence, false positives, or psychological effects.

#### 3.6.1. KQ1–PSA Screening vs. No Screening

##### Prostate Cancer Mortality

Seven articles, each based on independent data from different participant centers with no overlapping patients [32,71,77,79,81,82], originated from three RCTs (CAP, ERSPC, and PLCO). Collectively, these studies reported 0.96 fewer prostate cancer deaths per 1000 in individuals who were invited to participate in screening compared to those who were not invited (95% CI 1.52 fewer to 0.40 fewer) over a range of 9.5 to 22 years of follow-up. For participants who were 50 to 59 years of age, two articles [74,83] from one RCT (ERSPC) reported 1.25 fewer prostate cancer deaths per 1000 (95% CI 2.45 fewer to 0.5 more), while for those who were 60 to 69 years of age, the same RCTs reported 1.26 fewer deaths per 1000 (95% CI 2.70 fewer to 0.36 more) over a range of 9.5 to 16 years of follow-up. Only one RCT (ERSPC) [32] provided results on participants who were 70 years of age or older as a rate of person years and found that there were 0.09 more prostate cancer deaths per 1000 person-years over 16 years of follow-up. The overall GRADE ratings were low or very low due to concerns with risk of bias and imprecision (Supplementary File S9: Table S1, Figure S1).

One RCT (ERSPC) [77] provided results on prostate cancer mortality stratified by the number of screening rounds attended with 15 years of follow-up. The paper provided a hazard ratio as a relative measure and found that for no screening rounds attended, there were 4.64 more prostate cancer deaths per 1000 individuals who were invited to participate in screening compared to those who were not invited (95% CI 2 more to 8 more). For one screening round attended, there were 5.44 more prostate cancer deaths per 1000 (95% CI 2.64 more to 8.96 more), for two screening rounds attended, there were 4.16 fewer prostate cancer deaths per 1000 (95% CI 5.20 fewer to 2.72 fewer), and, for three screening rounds attended, there were 6.64 fewer prostate cancer deaths per 1000 (95% CI 7.28 fewer to 5.36 fewer). The overall GRADE ratings were rated as low due to concerns with risk of bias (Supplementary File S9: Table S2).

##### All-Cause Mortality

Five articles [32,71,74,75,81] from two RCTs (CAP and ERSC) reported 2.37 more deaths from any cause per 1000 individuals who were invited to participate in screening compared to those who were not invited (95% CI 4.74 fewer to 11.85 more) over a range of 9.5 to 22 years of follow-up. For participants who were 50 to 59 years of age, one article [74] from one RCT (ERSPC) reported a rate of 0.5 more deaths from any cause per 1000 person-years (95% CI 0.2 fewer to 1.3 more) over a median of 9.5 years of follow-up. For those who were 60 to 69 years of age, two articles [32,74] from one RCT (ERSPC) reported 0 fewer deaths per 1000 (95% CI 10.2 fewer to 10.2 more) over a range of 9.5 to 16 years of follow-up. No studies provided results on participants who were 70 years of age or older. The overall GRADE ratings were rated low due to concerns with risk of bias (Supplementary File S9: Table S3, Figures S2 and S3).

##### Metastatic Cancer

Four articles [75,76,78,79] from two RCTs (ERSPC and PLCO) reported 2.1 fewer metastatic cancers per 1000 individuals who were invited to participate in screening compared to those who were not invited (95% CI 3.25 fewer to 0.1 fewer) over a range of 15 to 21 years of follow-up. The GRADE rating was rated very low due to concerns with risk of bias and inconsistency across studies (Supplementary File S9: Table S4, Figure S4).

##### Overdiagnosis

One RCT [72] estimated overdiagnosis in participants invited to screen compared to those not invited to screen in the Finnish component of the ERSPC. Overdiagnosis was calculated using the “catch-up” method, defined as the relative excess cumulative incidence of prostate cancer cases in the invited-to-screen group at the point during follow-up when the cumulative incidence of prostate cancer in both groups stabilizes. In the 1929–1932 birth cohort, the cumulative incidence excess of prostate cancers was 4 more per 1000 (95% CI 11 fewer to 19 more) at 14 years of follow-up, yielding a relative diagnosis rate of 2.3%. At 10 years of follow-up, the incidence excess ranged between 26 more per 1000 (95% CI 13 more to 39 more, 15.4% relative overdiagnosis rate) in the 1933–1936 birth cohort and 10 more per 1000 (95% CI 2 more to 18 more, 10.3% relative overdiagnosis rate) in the 1941–1944 birth cohort. Outcomes for all groups were judged to be of very low certainty evidence due to risk of bias and imprecision (Supplementary File S9: Table S5).

One non-randomized study [84] estimated overdiagnosis for the screened cohort versus unscreened cohorts. To estimate overdiagnosis by age stratum, the study projected what prostate cancer incidence would have been without PSA screening by modeling and extrapolating trends prior to PSA screening introduction through to 1995 using linear regression by age and year. Excess cases were calculated by subtracting these predicted values from observed diagnoses, standardized to the U.S. population. For those aged 45 to 49 years, there was an observed incidence of 10,232 prostate cancer cases with screening and a predicted incidence of 4277 cases without screening, resulting in 5955 excess cases (95% CI 5,803 to 6,106) at 8 years of follow-up. For other age groups, the same study estimated 19,756 excess cases (95% CI 19,480 to 20,031) for those aged 50 to 54 years, 41,376 excess cases (95% CI: 40,977 to 41,774) for those aged 55 to 59 years, 77,014 excess cases (95% CI 76,471 to 77,558) for those aged 60 to 64 years, 116,263 excess cases (95% CI 115,596 to 116,930) for those aged 65 to 69 years, 113,188 excess cases (95% CI 112,530 to 113,846) for those aged 70 to 74 years, 58,798 excess cases (95% CI 58,324 to 59,273) for those aged 75 to 79 years, and 15,893 excess cases (95% CI 15,646 to 16,140) for those aged 80 years and older.

The non-randomized study also estimated overdiagnosis by dividing the risk of biopsy detected cancers from the screened Prostate Cancer Prevention Trial (PCPT) or ERSPC cohorts by the long-term risk of clinical prostate cancer, metastasis and cancer-specific mortality in the unscreened Malmö Preventive Program (MPP) cohort. When stratified by PSA level at age 60, overdiagnosis ranged between 110 more per 1000 (79 more to 152 more) for a PSA level of 1.0 ng/mL (RR 2.8, 95% CI: 2.3 to 3.5) to 67 fewer per 1000 (235 fewer to 0 fewer) for a PSA level of 10 ng/mL (RR 0.8, 95% CI 0.3 to 1.0) at 25 years of follow-up. All outcomes were judged to be of very low certainty evidence due to risk of bias (Supplementary File S9: Tables S6 and S7).

##### Quality of Life

One RCT [73] reported on the generic and disease-specific health-related quality of life (HRQOL) among individuals with prostate cancer in the screening arm and control arm of the population-based Finnish Randomized Study of Screening for Prostate Cancer (FinRSPC).

Prostate cancer-specific HRQOL was measured using the University of California, Los Angeles Prostate Cancer Index (UCLA-PCI) and consisted of six scales (urinary function, urinary bother, bowel function, bowel bother, sexual function, sexual bother). After re-scoring, the UCLA-PCI scale ranges from 0 to 100, with 100 representing normal healthy functioning or no bother. At 10 years of follow-up, the mean difference in cancer-specific HRQOL for those invited to PSA-screening compared to those not invited to screening ranged between 2 points lower (95% CI: 9 lower to 5 higher) and 6 points higher (95% CI: 1 higher to 11 higher) across the six scales. This indicated that cancer-specific HRQOL was slightly higher in those who received PSA-screening in all subscales except for “sexual bother”, but the differences were small. All outcomes were judged to be of low certainty evidence due to the risk of bias (Supplementary File S9: Table S9).

Generic HRQOL was measured using the RAND 36-Item Health Survey consisting of eight scales (physical functioning, role-physical, role-emotional, bodily pain, general health, vitality, social functioning, mental health). The RAND-36 scale was re-scored between 0 and 100, with 100 representing better functioning and less bother. At 10 years of follow-up, the mean difference based on adjusted mean scores in generic HRQOL for those invited to PSA-screening compared to those not invited to screening ranged between 2 points lower (95% CI: 9 lower to 5 higher) and 3 points higher (95% CI: 1 lower to 7 higher) across the eight scales. All outcomes were judged to be of low certainty evidence due to risk of bias (Supplementary File S9: Table S10).

#### 3.6.2. KQ2–PSA Screening vs. PSA Screening with Additional Testing

##### Overdiagnosis

One RCT provided data on overdiagnosis [85] from the STHLM3-MRI trial when comparing additional screening risk testing (PSA ≥ 3 ng/mL or Stockholm3 ≥ 11% followed by targeted biopsy) compared to usual care (no additional testing and standard biopsy). Overdiagnosis was defined as the detection of clinically insignificant cancers (Gleason score 6). At a follow-up of ≥ 200 days after receiving PSA results, the number of clinically insignificant cancers was lower in the experimental biopsy group than in the standard biopsy group (80 fewer per 1000 (95% CI 110 fewer to 50 fewer). The certainty of evidence was judged to be low due to risk of bias (Supplementary File S9: Table S8).

##### Complications Due to Biopsy

One RCT provided data on post-biopsy complications [85] from the STHLM3-MRI trial when comparing additional screening risk testing (PSA ≥ 3 ng/mL or Stockholm3 ≥ 11% followed by targeted biopsy) compared to usual care (no additional testing and standard biopsy). At a 30-day follow-up after the biopsy procedure, the number of infections was lower in the experimental biopsy group than in the standard biopsy group (20 fewer per 1000 patients; 95% CI: 40 fewer to 1 more). However, the certainty of this evidence was rated very low due to the risk of bias and imprecision (Supplementary File S9: Table S11). Similarly, at the 30-day follow-up, the number of post-biopsy hospitalizations was lower in the experimental biopsy group than the standard biopsy group (10 fewer cases per 1000 patients; 95% CI: 30 fewer to 1 fewer). The certainty of evidence was rated low due to the risk of bias (Supplementary File S9: Table S11).

Another study [86] also examined post-biopsy outcomes, but it included patients from the same STHLM3-MRI trial [85]. However, the population differed as it encompassed individuals with PSA < 3 and PSA ≥ 3 ng/mL/Stockholm3 ≥ 11%. At the 30-day follow-up after the biopsy, the experimental group experienced fewer post-biopsy infections and hospitalizations compared to the standard biopsy group (26 fewer infections per 1000 patients; 95% CI: 41 fewer to 11 fewer and 22 fewer hospitalizations per 1000 patients; 95% CI: 34 fewer to 9 fewer). The certainty of evidence was judged to be very low due to risk of bias associated with indirectness (Supplementary File S9: Table S11).

### 3.7. Sensitivity Analyses

We conducted sensitivity analyses by excluding studies assessed as having a high risk of bias, as well as the study by Hugosson et al. (2019) [32], which is a cumulative analysis of all ERSPC-centered trials. For each subgroup analysis, we performed additional sensitivity analysis restricted to RCTs with a low risk of bias, focusing on outcomes related to prostate-cancer mortality, all-cause mortality and metastatic cancer across different age groups. The findings remained consistent regardless of whether high risk of bias studies were included. Furthermore, excluding the Hugosson et al. (2019) [32] study for prostate cancer mortality and all-cause mortality resulted in reduced heterogeneity compared to the original subgroup analysis. Sensitivity analyses are presented in Supplementary File S10 (Figures S1–S9).

## 4. Discussion

### 4.1. Summary of Findings

This systematic review update and meta-analysis examined the benefits and harms of PSA-based prostate cancer screening compared with no screening, as well as the impact of incorporating additional information—such as risk stratification tools or MRI—into clinical decision-making following an elevated PSA result. The available evidence suggests that prostate cancer screening may provide benefits, including reductions in prostate cancer mortality and in the incidence of metastatic cancer, along with potential harms, including overdiagnosis, uncertain effects on quality of life, and associated complications.

In this updated review, the absolute effect for prostate cancer mortality was estimated at 0.96 fewer deaths per 1000 individuals invited to screening compared with those not invited, corresponding to a 12% relative reduction in the risk of prostate cancer mortality across all age groups over follow-up periods ranging from 9.5 to 22 years (RR 0.88 95% 0.81 to 0.95). Another study looked at prostate cancer mortality stratified by screening rounds attended and suggested that individuals who attended at least two screening rounds would see a reduction in the risk of prostate cancer mortality over 15 years of follow-up (HR 0.48 95% 0.35 to 0.66) [77]. For metastatic cancer outcomes, we found the absolute effect was 2.1 fewer metastatic cancers per 1000 individuals invited to screen compared to not invited for all ages over 15 to 21 years of follow-up (RR 0.58 95% CI 0.35 to 0.98). We did not find any studies that evaluated prostate cancer mortality or metastatic cancers from PSA screening when incorporating additional information following an elevated PSA test.

Few studies reported various overdiagnosis estimates and used different calculation methods, making it difficult to draw robust conclusions. One RCT estimated a range of 2.3% to 10.3% overdiagnosed cancers in the screening group stratified by birth cohort over 10 to 14 years of follow-up [72]. Only one study measured generic and disease-specific quality of life in individuals invited to screen compared to no invitation and found no major differences observed in quality of life between both groups during five to 15 years of follow-up [73]. Adverse events were noted in one RCT that compared standard biopsy to MRI with targeted and standard biopsy if the MRI results suggested prostate cancer and found fewer events (i.e., hospitalization or infection) in the intervention group with MRI [85,86].

The certainty of evidence was rated as low to very low certainty, indicating substantial uncertainty regarding the true effects of prostate cancer screening on the outcomes evaluated in this review. We found no evidence to assess the benefits and harms of screening in different ethnic and racial groups, nor did we identify any new RCTs compared to the previous 2014 review [47], the 2020 UK NSC review [51], or the 2013 Cochrane review [64]. The screening periods of the included trials that evaluated screening compared to no screening ranged between 1991 and 2003, while the STHLM3-MRI trial evaluated the addition of MRI within screening strategies where screening occurred between 2018 and 2020.

### 4.2. Current Practice and Challenges

Current practice for prostate cancer screening is characterized by considerable variability across jurisdictions and organizations, reflecting the uncertainty surrounding optimal approaches for balancing the benefits of early detection with the potential harms of overdiagnosis. The Canadian Task Force on Preventive Health Care and UK NSC recommend against routine PSA screening due to limited evidence of mortality benefits and concerns of harms, while the Canadian, American, and European Urological Association guidelines all recommend that individuals aged 50 to 69 years should be offered PSA screening with shared decision-making [57,88,89]. Conflicting guidance can lead to clinical uncertainty and inconsistent screening practices. One of the key challenges in guideline development is addressing equity considerations and identifying which individuals are most likely to benefit from screening, particularly given the indolent nature of many prostate cancers. Marginalized populations, including Black and Indigenous men, are known to be at a higher risk for aggressive prostate cancers and yet often face barriers to screening and remain underrepresented in screening programs and guideline recommendations [90,91,92]. Future guidelines should explicitly address equitable access to risk-adapted screening for these populations. The Canadian Urological Association Guidelines note the importance of equity and access but do not provide a formal framework for addressing screening disparities in Indigenous or other underserved populations. Implementation of personalized or risk-adapted screening strategies such as risk calculators have the potential to tailor screening strategies based on individual risk; however, widespread adoption is inconsistent, particularly in Canada [93]. In clinical practice, PSA testing is performed during the diagnostic evaluation of men presenting with urinary symptoms or prostate enlargement [57,94,95]. Given the high prevalence of urinary symptoms in older men, a substantial portion of men may undergo PSA testing outside the context of organized screening programs. While the scope of the present review was limited to PSA-based screening in asymptomatic men, widespread opportunistic testing should be considered when interpreting the overall impact of population-based screening strategies.

### 4.3. Implications

This review highlights the need for more uniform prostate cancer screening practices, especially in Canada. Policymakers and guideline developers should be aware of the limitations of the evidence base summarized in this review, which primarily stems from RCTs where screening was initiated between 1993 and 2009, prior to the adoption of newer techniques in clinical practice [96]. Since the release of the 2014 recommendation by the Canadian Task Force on Preventive Health Care, the screening landscape has evolved significantly with the introduction of MRI [97]. Research evaluating MRI within screening pathways remains limited, with few studies meeting our eligibility criteria in this review. It will be important for guideline and policy makers to revisit and refine screening recommendations to ensure they reflect current evidence and evolving clinical practice.

Estimating overdiagnosis and other harms, such as false positives, psychological distress, and related complications, remains a challenge in screening research and is highlighted in this review. It is difficult to accurately estimate overdiagnosis due to varying definitions, long lead times, and the need for a long enough follow-up time which many trials are unable to capture. These challenges can lead to inconsistent recommendations across guidelines, which supports the need for shared decision-making to understand the individual risk and potential outcomes. This review further highlights the need for updated, evidence-informed guidance that acknowledges evolving practices and supports equitable screening decisions.

### 4.4. Strengths and Limitations

An important strength of this review is its comprehensive and methodologically rigorous approach to synthesizing evidence on the benefits and harms of prostate cancer screening. We conducted and reported our review in accordance with established guidance, which ensures transparency, consistency, and completeness throughout the review process. We focused on RCT data and applied risk of bias certainty assessment to ensure robustness in our conclusions. The review also highlights recent developments in the field of screening, including emerging evidence on MRI-based screening strategies, which enhances its relevance to evolving clinical and policy contexts. However, the limitations of evidence from this review should be acknowledged. Most of the evidence on PSA-based screening originates from RCTs where screening occurred between 1993 and 2009 and does not account for newer technologies and risk-based approaches that are currently being explored. Studies that evaluate screening are difficult to conduct due to the large number of participants required, risk of contamination, and loss to follow-up. Additionally, few included studies evaluated the effects of PSA screening in different racial or ethnic groups, or by family history, and must be weighed by guideline developers when examining the current evidence. Additionally, the limitations of this review include that we did not seek to evaluate patient values and preferences through the evidence or patient engagement. It will be important to consider the patient perspective when formulating recommendations, especially in patients who have been screened but did not develop cancer.

## 5. Conclusions

In summary, this systematic review highlights the challenges of prostate cancer screening, particularly in balancing the potential benefits of disease-specific mortality against the risks of overdiagnosis and associated harms. While evidence from large, randomized trials suggests screening might reduce prostate cancer mortality at 9.5 to 22 years, the overall certainty of the evidence was low, limiting our confidence in the magnitude and consistency of these effects. Estimates of harms, including overdiagnosis and adverse events, remain imprecise and highly variable across studies. Advances in other technologies, such as mpMRI and risk-based screening strategies, offer promise in improving the benefits and reducing the harms of screening; however, high-quality evidence on their long-term impact remains limited, as we were unable to draw robust conclusions from this review. Given these conclusions, screening decisions should emphasize individualized risk assessment and shared decision-making. Future research is needed to strengthen the evidence base, notably in evaluating newer technologies, long-term outcomes, and screening impacts in high-risk and underrepresented populations, which is essential for developing updated, evidence-informed, and equitable screening guidance.

## Figures and Tables

**Table 1:** Eligibility criteria *.

	KQ1	KQ2
**Population**	Adults (≥18 years) not known to be at elevated risk for prostate cancer. Secondary analyses for decision-making: Screening interval (KQ1); PSA thresholds (KQ1b); Age: <55 years, 55–69 years, ≥70 years (KQ1c); Race and/or ethnicity (KQ1d); Obesity, as defined by study authors (KQ1d); Family history (KQ1d) Exclusion: Individuals with pre-existing or previous history of prostate cancer. Individuals specifically selected for the presence of another condition or risk factor (i.e., individuals working with chemicals known to be carcinogenic or individuals with known genetic markers). Individuals who have had a previous PSA screen and/or individuals with a “normal” change in urine function are not excluded. Normal will be defined by clinician judgement.	Adults (≥18 years) with an elevated * PSA test * Definition of elevated to be determined by included study. Secondary analyses for decision-making: PSA thresholds; Age: <55 years, 55–69 years, ≥70 years; Race and/or ethnicity; Obesity, as defined by study authors; Family history Exclusion: Individuals with history of prostate cancer. Individuals who have had a previous PSA screen are not excluded. Individuals specifically selected for the presence of another condition or risk factor (e.g., other types of cancer, individuals working with chemicals known to be carcinogenic, individuals with known genetic risk)
**Interventions**	One or more clinical or lab test (e.g., PSA+DRE, PSA alone, DRE alone) with or without additional tests before biopsy. Exclusion: Other screening method that does not include PSA or DRE.	Additional testing (e.g., risk stratification, MRI). Tests used alone, sequentially or in combination to determine the need for biopsy, including but not limited to: Clinical variables (e.g., age, family history of prostate cancer, a previous biopsy); Ratio of free to total PSA; Blood biomarkers (PSA, MIC1 etc.) or biomarker panels (4K panel, STHLM3 panel); Urine biomarkers; Genetic markers; DRE; Prostate volume; Imaging markers/techniques (e.g., mp-MRI); Nomograms combining one or more of the above variables or tests. Exclusion: Any post-biopsy intervention (e.g., MRI that stratifies risk of an already diagnosed cancer)
**Comparator**	No screening, usual care, or alternate type of screening within the options previously stated (e.g., DRE alone) (KQ1a)	No additional testing (PSA-based screening only (including single threshold PSA test, age-specific thresholds, variable screening intervals)) or usual care
**Outcomes**	Potential benefits: Reduced prostate cancer mortality Reduced all-cause mortality Reduced incidence of metastatic cancer Potential harms: False positives Overdiagnosis Complications due to biopsy Incontinence (urinary or bowel) Erectile dysfunction Either benefit or harm: Quality of life or functioning (overall and disease-specific *) Psychological effects As defined/reported by study authors. * Scales with acceptable measurement properties (e.g., validity, reliability) for use in prostate cancer	Potential benefits: Reduced prostate cancer mortality Reduced all-cause mortality Reduced incidence of metastatic cancer Potential harms: False positives Overdiagnosis Complications due to biopsy Incontinence (urinary or bowel) Erectile dysfunction Either benefit or harm: Quality of life or functioning (overall and disease-specific *) Psychological effects As defined/reported by study authors. * Scales with acceptable measurement properties (e.g., validity, reliability) for use in prostate cancer
**Timing of** **Outcome assessment**	Any timing	Any timing
**Setting**	Primary care settings Exclusion: Settings not generalizable to primary care	Primary care settings Exclusion: Settings not generalizable to primary care
**Study design**	Benefits and harms: Randomized (including cluster RCTs), quasi-randomized, and controlled clinical trials Harms only: Cohort studies Exclusion: Editorials, commentaries, letters, conference proceedings, government reports, case series, case report, narrative reviews, systematic reviews	RCTs (including cluster RCTs), observational studies with consecutively enrolled populations Exclusion: Case reports, case series, systematic reviews, narrative reviews, editorials, commentaries, letters, conference proceedings, government reports.
**Language**	English or French	English or French
**Dates of** **publication**	2019 to present (RCTs) 2012 to present (nRCTs)	2019 to present (RCTs) 2012 to present (nRCTs)

* Note: Eligibility criteria were adapted from our previously published protocol [62], available under the Creative Commons Attribution 4.0 International l License (CC BY 4.0).

## Data Availability

Any data or materials can be found on the Open Science Framework (https://osf.io/dm32k/).

## Supplementary Materials

The following supporting information can be downloaded at: https://www.mdpi.com/article/10.3390/curroncol33040199/s1, Supplementary File S1: PRISMA 2020 checklist; Supplementary File S2: Completed PRESS form; Supplementary File S3: Database search strategies; Supplementary File S4: List of grey literature sources; Supplementary File S5: Data extraction items; Supplementary File S6: List of excluded studies; Supplementary File S7 (Table S1): Study characteristics table; Supplementary File S8 (Table S1): RoB for included RCTs, Supplementary File S8 (Table S2): RoB for included non-randomized study; Supplementary File S9 (Table S1): GRADE table for Prostate Cancer mortality outcome by age (KQ1, RCTs), Supplementary File S9 (Figure S1): Forest plot for Prostate Cancer mortality outcome by age (KQ1, RCTs), Supplementary File S9 (Table S2): GRADE table for Prostate Cancer mortality outcome by screening rounds (KQ1, RCTs), Supplementary File S9 (Table S3): GRADE table for All-cause mortality outcome by all ages and age-subgroup (KQ1, RCTs), Supplementary File S9 (Figure S2): Forest plot for all-cause mortality outcome for all ages (KQ1, RCTs), Supplementary File S9 (Figure S3) Forest plot for all-cause mortality outcome for age sub-group (KQ1, RCTs), Supplementary File S9 (Table S4): GRADE table for Metastatic cancer outcome for all ages (KQ1, RCTs), Supplementary File S9 (Figure S4): Forest plot for Metastatic cancer outcome for all ages (KQ1, RCTs), Supplementary File S9 (Table S5): GRADE table for Overdiagnosis outcome (KQ1, RCTs), Supplementary File S9 (Table S6): GRADE table for the overdiagnosis outcome by age (KQ1, non-randomized study), Supplementary File S9 (Table S7): GRADE table for the overdiagnosis outcome by PSA levels (KQ1, non-randomized study), Supplementary File S9 (Table S8): GRADE table for the overdiagnosis outcome with the additional screening test (KQ2, RCTs), Supplementary File S9 (Table S9) GRADE table for the Quality of life outcome based on a UCLA-PCI survey (KQ1, RCTs), Supplementary File S9 (Table S10) GRADE table for the Quality of life outcome based on RAND-36 survey (KQ1, RCTs), Supplementary File S9 (Table S11) GRADE table for adverse events outcome post-biopsy (KQ2, RCTs); Supplementary File S10: Sensitivity analyses for different outcomes (Prostate cancer mortality, all-cause mortality and metastatic cancer; Figures S1–S9).
