# Supplementary material for: The Benefits and Harms of Screening for Prostate Cancer in Adults Aged 18 Years and Older: A Systematic Review
Source: Curr Oncol. 2026 Mar 31;33(4):199. doi: 10.3390/curroncol33040199 (PMC13115368; doi:10.3390/curroncol33040199)

Supplementary Materials:

Supplementary File S1. PRISMA 2020 checklist 3

Supplementary File S2: Completed PRESS form 6

Supplementary File S3: Database search strategies 19

Supplementary File S4: List of grey literature sources 25

Supplementary File S5: Data Extraction Items 26

Supplementary File S6: List of excluded studies with reasons 28

Ineligible study design (n=291) 28

RCT published prior to 2019 (n=154) 55

Ineligible outcomes (n=85) 73

PSA or DRE was not used as the screening method (n=36) 83

Cohort study published prior to 2012 (n=25) 87

Language other than English or French (n=21) 90

Patients have previous history or pre-existing prostate cancer (n=14) 92

Patients were selected based on known condition or risk factor (n=8) 93

Ineligible comparator group (n=5) 95

Duplicate of already included study (n=1) 95

Supplementary File S7: Study characteristics table 96

Supplementary File S8: Risk of Bias Summary 102

Supplementary File S9: GRADE Evidence Profile Tables and Forest Plots 103

Table S1: KQ1 Prostate cancer mortality (RCTs stratified by age) 103

Figure S1. KQ1 Prostate cancer mortality (RCTs) - All ages (follow-up: range 9.5 years to 22 years) 105

Table S2: KQ1 Prostate cancer mortality (RCTs stratified by screening rounds attended) 106

Table S3: KQ1 All-cause mortality (RCTs) 108

Figure S2: KQ1 All-cause mortality (RCTs) - All ages (follow-up: range 9.5 years to 22 years) 109

Figure S3: KQ1 All-cause mortality (RCTs) - Subgroup: 60-69 years (follow-up: range 9.5 years to 16 years) 110

Table S4: KQ1 Metastatic cancer (RCTs) 111
Figure S4: KQ1 Metastatic cancer (RCTs) - All ages (follow-up: range 15 years to 21 years) 112
Table S5: KQ1 Overdiagnosis (RCTs stratified by birth cohort) 113
Table S6: KQ1 Overdiagnosis (non-randomized study stratified by age) 115
Table S7: KQ1 Overdiagnosis (non-randomized study stratified by PSA level at age 60) 118
Table S8: KQ2 Overdiagnosis (RCTs) 121
Table S9: KQ1 Quality of life (RCTs, cancer specific, UCLA-PCI survey) 122
Table S10: KQ1 Quality of life (RCTs, generic health, RAND-36 survey) 124
Table S11: KQ2 Adverse events (post-biopsy) 127
Bibliography 129
Supplementary File S10: Sensitivity Analyses 130
Figure S1. RCT - Prostate cancer mortality, all ages (low RoB RCTs) 130
Figure S2. RCT - Prostate cancer mortality, all ages (without Hugosson et al., 2019) 130
Figure S3. Prostate cancer mortality, 50-59 years (low RoB RCTs) 130
Figure S4. Prostate cancer mortality, 60-69 years (low RoB RCTs) 130
Figure S5. All-cause mortality, all ages (low RoB RCTs) 131
Figure S6. All-cause mortality, all ages (without Hugosson et al., 2019) 131
Figure S7. All-cause mortality, 60-69 years (low RoB studies) 131
Figure S8. All-cause mortality, 60-69 years (without Hugosson et al., 2019) 131
Figure S9. Metastatic cancer, all ages (low RoB RCTs) 131

Supplementary File S1. PRISMA 2020 checklist

Section and Topic	Item #	Checklist item	Pg #
**TITLE**			
Title	1	Identify the report as a systematic review.	1
**ABSTRACT**			
Abstract	2	See the PRISMA 2020 for Abstracts checklist.	1, 2
**INTRODUCTION**			
Rationale	3	Describe the rationale for the review in the context of existing knowledge.	2 - 4
Objectives	4	Provide an explicit statement of the objective(s) or question(s) the review addresses.	3, 4
**METHODS**			
Eligibility criteria	5	Specify the inclusion and exclusion criteria for the review and how studies were grouped for the syntheses.	4 - 6
Information sources	6	Specify all databases, registers, websites, organisations, reference lists and other sources searched or consulted to identify studies. Specify the date when each source was last searched or consulted.	7
Search strategy	7	Present the full search strategies for all databases, registers and websites, including any filters and limits used.	7, S3, S4
Selection process	8	Specify the methods used to decide whether a study met the inclusion criteria of the review, including how many reviewers screened each record and each report retrieved, whether they worked independently, and if applicable, details of automation tools used in the process.	7
Data collection process	9	Specify the methods used to collect data from reports, including how many reviewers collected data from each report, whether they worked independently, any processes for obtaining or confirming data from study investigators, and if applicable, details of automation tools used in the process.	7, S5
Data items	10a	List and define all outcomes for which data were sought. Specify whether all results that were compatible with each outcome domain in each study were sought (e.g. for all measures, time points, analyses), and if not, the methods used to decide which results to collect.	7, 8
	10b	List and define all other variables for which data were sought (e.g. participant and intervention characteristics, funding sources). Describe any assumptions made about any missing or unclear information.	7, 8
Study risk of bias assessment	11	Specify the methods used to assess risk of bias in the included studies, including details of the tool(s) used, how many reviewers assessed each study and whether they worked independently, and if applicable, details of automation tools used in the process.	8

Effect measures	12	Specify for each outcome the effect measure(s) (e.g. risk ratio, mean difference) used in the synthesis or presentation of results.	8
Synthesis methods	13a	Describe the processes used to decide which studies were eligible for each synthesis (e.g. tabulating the study intervention characteristics and comparing against the planned groups for each synthesis (item #5)).	8
	13b	Describe any methods required to prepare the data for presentation or synthesis, such as handling of missing summary statistics, or data conversions.	8
	13c	Describe any methods used to tabulate or visually display results of individual studies and syntheses.	8, 9
	13d	Describe any methods used to synthesize results and provide a rationale for the choice(s). If meta-analysis was performed, describe the model(s), method(s) to identify the presence and extent of statistical heterogeneity, and software package(s) used.	9
	13e	Describe any methods used to explore possible causes of heterogeneity among study results (e.g. subgroup analysis, meta-regression).	9
	13f	Describe any sensitivity analyses conducted to assess robustness of the synthesized results.	9
Reporting bias assessment	14	Describe any methods used to assess risk of bias due to missing results in a synthesis (arising from reporting biases).	N/A
Certainty assessment	15	Describe any methods used to assess certainty (or confidence) in the body of evidence for an outcome.	9, 10
**RESULTS**			
Study selection	16a	Describe the results of the search and selection process, from the number of records identified in the search to the number of studies included in the review, ideally using a flow diagram.	10, 11
	16b	Cite studies that might appear to meet the inclusion criteria, but which were excluded, and explain why they were excluded.	S6
Study characteristics	17	Cite each included study and present its characteristics.	11, 12, S7
Risk of bias in studies	18	Present assessments of risk of bias for each included study.	12, S8
Results of individual studies	19	For all outcomes, present, for each study: (a) summary statistics for each group (where appropriate) and (b) an effect estimate and its precision (e.g. confidence/credible interval), ideally using structured tables or plots.	12 - 15
Results of syntheses	20a	For each synthesis, briefly summarise the characteristics and risk of bias among contributing studies.	12 - 15

	20b	Present results of all statistical syntheses conducted. If meta-analysis was done, present for each the summary estimate and its precision (e.g. confidence/credible interval) and measures of statistical heterogeneity. If comparing groups, describe the direction of the effect.	12 – 15, S9
	20c	Present results of all investigations of possible causes of heterogeneity among study results.	12 – 15, S9
	20d	Present results of all sensitivity analyses conducted to assess the robustness of the synthesized results.	15, S10
Reporting biases	21	Present assessments of risk of bias due to missing results (arising from reporting biases) for each synthesis assessed.	N/A
Certainty of evidence	22	Present assessments of certainty (or confidence) in the body of evidence for each outcome assessed.	12- 15, S9
**DISCUSSION**			
Discussion	23a	Provide a general interpretation of the results in the context of other evidence.	16
	23b	Discuss any limitations of the evidence included in the review.	17, 18
	23c	Discuss any limitations of the review processes used.	17, 18
	23d	Discuss implications of the results for practice, policy, and future research.	17
**OTHER INFORMATION**			
Registration and protocol	24a	Provide registration information for the review, including register name and registration number, or state that the review was not registered.	4
	24b	Indicate where the review protocol can be accessed, or state that a protocol was not prepared.	4
	24c	Describe and explain any amendments to information provided at registration or in the protocol.	10
Support	25	Describe sources of financial or non-financial support for the review, and the role of the funders or sponsors in the review.	19
Competing interests	26	Declare any competing interests of review authors.	19
Availability of data, code and other materials	27	Report which of the following are publicly available and where they can be found: template data collection forms; data extracted from included studies; data used for all analyses; analytic code; any other materials used in the review.	19

From: Page MJ, McKenzie JE, Bossuyt PM, Boutron I, Hoffmann TC, Mulrow CD, et al. The PRISMA 2020 statement: an updated guideline for reporting systematic reviews. BMJ 2021;372:n71. doi: 10.1136/bmj.n71 For more information, visit: http://www.prisma-statement.org/

Supplementary File S2: Completed PRESS form

***PRESS Guideline* 2015— Search Submission & Peer Review Assessment**

Reference: McGowan J, Sampson M, Salzwedel DM, Cogo E, Foerster V, Lefebvre C. PRESS Peer Review of Electronic Search Strategies: 2015 guideline statement. *J Clin Epidemiol* 2016;75:40-6. Available: http://www.jclinepi.com/article/S0895-4356(16)00058-5/pdf.

Search submission: This section to be filled in by the searcher

Searcher: Becky Skidmore Email: bskidmore@rogers.com

Date submitted: 2022 May 27 Date requested by: 2022 May 30

1. **Systematic Review Title**

Screening for prostate cancer: protocol for updating multiple systematic reviews to inform a Canadian Task Force on Preventive Health Care guideline update (KQ1 & 2)

2. **This search strategy is …**

X	My PRIMARY (core) database strategy — First time submitting a strategy for search question and database
	My PRIMARY (core) strategy — Follow-up review NOT the first time submitting a strategy for search question and database. If this is a response to peer review, itemize the changes made to the review suggestions
	SECONDARY search strategy— First time submitting a strategy for search question and database
	SECONDARY search strategy — NOT the first time submitting a strategy for search question and database. If<br>this is a response to peer review, itemize the changes made to the review suggestions

3. **Database** (e.g., MEDLINE, CINAHL) *[mandatory]*

MEDLINE

4. **Interface** (e.g., Ovid, EbscoHost…) *[mandatory]*

Ovid

Research Question (Describe the purpose of the search) *[mandatory]*

KQ1

What are the benefits and harms of prostate cancer screening?

a) Do the benefits and harms differ by screening modalities (i.e., PSA alone, DRE alone, or PSA+DRE)?

b) In screening with PSA, do benefits and harms differ by PSA threshold value?

c) Do the benefits and harms differ by age group (<55 years, 55-69 years, ≥70 years)?

d) Do the benefits and harms differ by other risk factors such as race/ethnicity and/or family history?

KQ2

What are the benefits and harms of incorporating additional information (e.g., risk stratification, MRI) into clinical decision making following an elevated PSA test?

6. **PICO Format** Outline the PICOs for your question — i.e., Patient, Intervention, Comparison, Outcome, and Study Design — as applicable

KQ1

**P**	Individuals not known to be at elevated risk for prostate cancer.<br>*Secondary analyses for decision-making*:<br>• Screening interval (KQ1)<br>• PSA thresholds (KQ1b)<br>• Age: <55 years, 55-69 years, ≥70 years (KQ1c)<br>• Race and/or ethnicity (KQ1d)<br>• Obesity, as defined by study authors (KQ1d)<br>Family history (KQ1d)
**I / Exposure**	One or more clinical or lab test (e.g., PSA+DRE, PSA alone, DRE alone) with or without additional tests before biopsy.
**C**	No screening<br>Usual care<br>Alternate type of screening within the options previously stated (e.g., DRE alone) [KQ1a]
**O**	<u>Potential benefits</u><br>1. Reduced prostate cancer mortality<br>2. Reduced all-cause mortality<br>3. Reduced incidence of metastatic cancer<br><br><u>Potential harms</u><br>4. False positives<br>5. Overdiagnosis[a]<br>6. Complications due to biopsy<br>7. Incontinence (urinary or bowel)<br>8. Erectile dysfunction

	Either benefit or harm<br>9. Quality of life or functioning (overall and disease-specific*)<br>10. Psychological effects<br><br>*As defined/reported by study authors.*<br><br>**scales with acceptable measurement properties (e.g., validity, reliability) for use in prostate cancer*
**S**	Benefits and harms<br>Randomized (including cluster RCTs), quasi-randomized, and controlled clinical trials<br><br>Harms only<br>Cohort studies (if needed)[b]

[b]If certainty in the evidence is a barrier to the development of recommendations and the CTFPHC believes that further evidence from cohort studies may influence their recommendations.

KQ2

	Inclusion criteria	Exclusion criteria
**Population**	Individuals with an elevated* PSA test<br><br>**definition of elevated to be determined by included study*<br><br>*Secondary analyses for decision-making*:<br>• PSA thresholds	Individuals <18 years. Individuals with history of prostate cancer. Individuals who have had a previous PSA screen are not excluded.<br>Individuals specifically selected for the presence of another condition or risk factor (e.g., other types of cancer, individuals working with chemicals known to be carcinogenic, individuals with known genetic risk)

	• Age: <55 years, 55-69 years, ≥70 years<br>• Race and/or ethnicity<br>• Obesity, as defined by study authors<br>• Family history	
**Interventions**	Additional testing (e.g., risk stratification, MRI)<br>Tests used alone, sequentially or in combination to determine the need for biopsy, including but not limited to: Clinical variables (e.g., age, family history of prostate cancer, a previous biopsy), ratio of free to total PSA, Blood biomarkers (PSA, MIC1 etc.) or biomarker panels (4K panel, STHLM3 panel), Urine biomarkers, Genetic markers, DRE, Prostate volume, Imaging markers/techniques (e.g., mp-MRI), Nomograms combining one or more of the above variables or tests.	Any post-biopsy intervention (e.g., MRI that stratifies risk of an already diagnosed cancer)
**Comparator**	No additional testing (PSA-based screening only (including single threshold PSA test, age-specific thresholds, variable screening intervals))<br>Usual care	N/A
**Outcomes**	<u>Potential benefits</u><br>1. Reduced prostate cancer mortality	N/A

	2. Reduced all-cause mortality<br>3. Reduced incidence of metastatic cancer<br><br><u>Potential harms</u><br>4. False positives<br>5. Overdiagnosis[a]<br>6. Complications due to biopsy<br>7. Incontinence (urinary or bowel)<br>8. Erectile dysfunction<br><br><u>Either benefit or harm</u><br>9. Quality of life or functioning (overall and disease-specific*)<br>10. Psychological effects<br><br>*As defined/reported by study authors.*<br><br>**scales with acceptable measurement properties (e.g., validity, reliability) for use in prostate cancer*	
**Timing of outcome assessment**	Any timing	N/A

**Delivery setting**	Primary care settings	Settings not generalizable to primary care
**Study design**	RCTs (including cluster RCTs), observational studies with consecutively enrolled populations	Case reports, case series, systematic reviews[b], narrative reviews, editorials, commentaries, letters, conference proceedings, government reports.
**Language**	English or French	N/A
**Dates of publication**	All dates	N/A

7. **Inclusion Criteria** (List criteria such as age groups, study designs, etc., to be included) *[optional]*

8. **This search strategy is …**

All dates are included (as per the UK NSC search), however, the current RCT update will include 2019 to present; nRCTs from June 2012 to present.

Languages – English or French (Note – Language limits not applied within search)

9. **Exclusion Criteria** (List criteria such as study designs, date limits, etc., to be excluded) **[optional]**

Case reports, case series, systematic reviews[b], narrative reviews, editorials, commentaries, letters, conference proceedings, government reports.

10. ■ **Was a search filter applied?** Yes

In-h

If YES, which one(s) (e.g., Cochrane RCT filter, PubMed Clinical Queries filter)? Provide the source if this is a published filter. *[mandatory if YES to previous question — textbox]*

Cochrane 2008 HSSS strategy, sensitivity- and precision-maximizing version, slightly amended

11. **Notes or comments you feel would be useful for the peer reviewer** *[optional]*

This search is a combination of two different searches and refined for our needs. It was felt that both KQs could be addressed in this one search as PSA testing is included.

12. **Please copy and paste your search strategy here, exactly as run, including the number of hits per line. [mandatory]**

Database: Ovid MEDLINE(R) ALL <1946 to May 26, 2022>

Search Strategy:

----

1 exp Prostatic Neoplasms/ (142521)

2 (prostat* adj3 (neoplas* or cancer* or carcinoma* or adenocarcinom* or tumour* or tumor* or malignan* or metasta* or angiosarcoma* or sarcoma* or teratoma* or lymphoma* or blastoma* or microcytic* or leiomyosarcoma* or lump?)).tw,kf. (165230)

3 (PIN and (prostat* or intraepithelial or neoplas*)).tw,kf. (1315)

4 or/1-3 [PROSTATE CANCER] (187281)

5 Mass Screening/ (113531)

6 "Early Detection of Cancer"/ (33829)

7 (screen* or detect*).tw,kf. (3304523)

8 (identif* or recogni*).ti. (432439)

9 ((early or earlier or earliest) adj5 (identif* or recogni*)).tw,kf. (82757)

10 (case finding? or casefinding?).tw,kf. (6044)

11 exp Prostatic Neoplasms/di, pc [Diagnosis, Prevention & Control] (23960)

12 Prostatic Intraepithelial Neoplasia/di, pc [Diagnosis, Prevention & Control] (280)

13 Digital Rectal Examination/ (1017)

14 (rect* adj2 exam*).tw,kf. (6998)

15 DRE.tw,kf. (3356)

16 Palpation/ (7853)

17 palpat*.tw,kf. (16289)

18 ((transrectal* or trans-rectal*) adj4 (ultrasonograph* or ultrasound* or ultra-sonograph* or ultra-sound*)).tw,kf. (8699)

19 (TRUS or TRUSB).tw,kf. (3218)

20 ((prostat* specific antigen* or PSA) adj3 (diagnos* or screen* or test* or elevat* or level? or score? or result?)).tw,kf. (22503)

21 Prostate-Specific Antigen/ and (Diagnostic Tests, Routine/ or "Diagnostic Techniques and Procedures"/) (83)

22 or/5-21 [SCREENING] (3765903)

23 4 and 22 [PROSTATE CANCER - SCREENING] (65373)

24 (controlled clinical trial or randomized controlled trial or pragmatic clinical trial or equivalence trial).pt. (660567)

25 "Clinical Trials as Topic"/ (199944)

26 exp "Controlled Clinical Trials as Topic"/ (164991)

27 (randomi#ed or randomi#ation? or randomly or RCT or placebo*).tw,kf. (1131530)

28 ((singl* or doubl* or trebl* or tripl*) adj (mask* or blind* or dumm*)).tw,kf. (188875)

29 trial.ti. (263006)

30 or/24-29 (1583534)

31 23 and 30 [PROSTATE CANCER - SCREENING - RCTs] (6039)

32 (2019082* or 2019083* or 201909* or 201910* or 201911* or 201912* or 202*).dt. (4131938)

33 31 and 32 [RCTs - UPDATE PERIOD] (734)

34 controlled clinical trial.pt. (94882)

35 Controlled Clinical Trial/ or Controlled Clinical Trials as Topic/ (100471)

36 (control* adj2 trial).tw,kf. (192023)

37 Non-Randomized Controlled Trials as Topic/ (1046)

38 (nonrandom* or non-random* or quasi-random* or quasi-experiment*).tw,kf. (66824)

39 (nRCT or non-RCT).tw,kf. (430)

40 Controlled Before-After Studies/ (697)

41 (control* adj3 ("before and after" or "before after")).tw,kf. (4916)

42 Interrupted Time Series Analysis/ (1625)

43 time series.tw,kf. (40099)

44 (pre- adj3 post-).tw,kf. (102298)

45 (pretest adj3 posttest).tw,kf. (6687)

46 Historically Controlled Study/ (222)

47 (control* adj2 study).tw,kf. (196842)

48 Control Groups/ (1830)

49 (control* adj2 group?).tw,kf. (577755)

50 trial.ti. (263006)

51 or/34-50 (1270617)

52 23 and 51 [PROSTATE CANCER - SCREENING - nRCTs] (4065)

53 (201206* or 201207* or 201208* or 201209* or 201210* or 201211* or 201212* or 2013* or 2014* or 2015* or 2016* or 2017* or 2018* or 2019* or 202*).dt. (12353163)

54 52 and 53 [nRCTs - UPDATE PERIOD] (2091)

55 33 or 54 [RCTS, nRCTs] (2488)

56 exp Animals/ not Humans/ (5010745)

57 55 not 56 [ANIMAL-ONLY REMOVED] (2464)

58 (comment or editorial or news or newspaper article or case reports).pt. (3844934)

59 (letter not (letter and randomized controlled trial)).pt. (1175623)

60 57 not (58 or 59) [OPINION PIECES REMOVED] (2360)

****

Peer review assessment: this section to be filled in by the reviewer

Reviewer: Kaitryn Campbell	Email: kcamlolo668@gmail.com	Date completed: 28 May 2022

Do you wish to be acknowledged? (If yes, the review team will be advised to add an acknowledgement to any publications related to this work). Yes please.

The suggested acknowledgement is “We thank Kaitryn Campbell, MLIS, MSc (St. Joseph’s Healthcare Hamilton/McMaster University) for peer review of the MEDLINE search strategy.”

1. TRANSLATION

	A --No revisions	X
	B -- Revision(s) suggested	
	C -- Revision(s) required	

If “B” or “C,” please provide an explanation or example:

A --No revisions	X
B -- Revision(s) suggested	
C -- Revision(s) required	

If "B" or "C," please provide an explanation or example:

3. SUBJECT HEADINGS

A --No revisions	X
B -- Revision(s) suggested	
C -- Revision(s) required	

If "B" or "C," please provide an explanation or example:

4. TEXT WORD SEARCHING

A --No revisions	
B -- Revision(s)suggested	X
C -- Revision(s) required	

If "B" or "C," please provide an explanation or example:

Throughout, just wondering why you chose to use tw,kf. instead of tw,kf,kw.

Line 2, suggest hyphenate: adeno-carcinom*, angio-sarcoma*

Line 8, suggest add .kf,kw. to .ti.

5. SPELLING, SYNTAX, AND LINE NUMBERS

A --No revisions	X
B -- Revision(s)suggested	
C -- Revision(s) required	

If "B" or "C," please provide an explanation or example:

A --No revisions	X
B -- Revision(s) suggested	
C -- Revision(s) required	

If “B” or “C,” please provide an explanation or example:

A --No revisions	
B -- Revision(s) suggested	X
C -- Revision(s) required	

Additional comments:

Nicely done (clean & sensible to combine the searches for both questions). No errors or omissions detected and just a couple of suggestions made.

Supplementary File S3: Database search strategies

Prostate Cancer
KQ1 & 2
Final Strategies – Appendix 1
2022 May 30

Ovid Multifile

Database: Embase Classic+Embase <1947 to 2022 May 27>, Ovid MEDLINE(R) ALL <1946 to May 27, 2022>, EBM Reviews - Cochrane Central Register of Controlled Trials <April 2022>
Search Strategy:
----

1 exp Prostatic Neoplasms/ (426243)
2 (prostat* adj3 (neoplas* or cancer* or carcinoma* or adenocarcinom* or adeno-carcinom* or tumour* or tumor* or malignan* or metasta* or angiosarcoma* or angio-sarcoma* or sarcoma* or teratoma* or lymphoma* or blastoma* or microcytic* or leiomyosarcoma* or lump?)).tw,kw,kf. (430955)
3 (PIN and (prostat* or intraepithelial or neoplas*)).tw,kw,kf. (3545)
4 or/1-3 [PROSTATE CANCER] (513167)
5 Mass Screening/ (181952)
6 "Early Detection of Cancer"/ (44407)
7 (screen* or detect*).tw,kw,kf. (7868673)
8 (identif* or recogni*).ti,kw,kf. (1022950)
9 ((early or earlier or earliest) adj5 (identif* or recogni*)).tw,kw,kf. (213030)
10 (case finding? or casefinding?).tw,kw,kf. (15112)
11 exp Prostatic Neoplasms/di, pc [Diagnosis, Prevention & Control] (65584)
12 Prostatic Intraepithelial Neoplasia/di, pc [Diagnosis, Prevention & Control] (853)
13 Digital Rectal Examination/ (11506)
14 (rect* adj2 exam*).tw,kw,kf. (19482)
15 DRE.tw,kw,kf. (9337)
16 Palpation/ (31804)
17 palpat*.tw,kw,kf. (47171)
18 ((transrectal* or trans-rectal*) adj4 (ultrasonograph* or ultrasound* or ultra-sonograph* or ultra-sound*)).tw,kw,kf. (23238)
19 (TRUS or TRUSB).tw,kw,kf. (10744)
20 ((prostat* specific antigen* or PSA) adj3 (diagnos* or screen* or test* or elevat* or level? or score? or result?)).tw,kw,kf. (65060)
21 Prostate-Specific Antigen/ and (Diagnostic Tests, Routine/ or "Diagnostic Techniques and Procedures"/) (1065)
22 or/5-21 [SCREENING] (8978491)
23 4 and 22 [PROSTATE CANCER - SCREENING] (181823)

24 (controlled clinical trial or randomized controlled trial or pragmatic clinical trial or equivalence trial).pt. (1305497)
25 "Clinical Trials as Topic"/ (322120)
26 exp "Controlled Clinical Trials as Topic"/ (409594)
27 (randomi#ed or randomi#ation? or randomly or RCT or placebo*).tw,kw,kf. (3973714)
28 ((singl* or doubl* or trebl* or tripl*) adj (mask* or blind* or dumm*)).tw,kw,kf. (789920)
29 trial.ti. (1000703)
30 or/24-29 (4955154)
31 23 and 30 [PROSTATE CANCER - SCREENING - RCTs] (20390)
32 (2019082* or 2019083* or 201909* or 201910* or 201911* or 201912* or 202*).dt. (4138387)
33 31 and 32 [RCTs - UPDATE PERIOD] (734)
34 controlled clinical trial.pt. (188023)
35 Controlled Clinical Trial/ or Controlled Clinical Trials as Topic/ (577691)
36 (control* adj2 trial).tw,kw,kf. (1108798)
37 Non-Randomized Controlled Trials as Topic/ (13466)
38 (nonrandom* or non-random* or quasi-random* or quasi-experiment*).tw,kw,kf. (169212)
39 (nRCT or non-RCT).tw,kw,kf. (1134)
40 Controlled Before-After Studies/ (243239)
41 (control* adj3 ("before and after" or "before after")).tw,kw,kf. (13035)
42 Interrupted Time Series Analysis/ (236395)
43 time series.tw,kw,kf. (87003)
44 (pre- adj3 post-).tw,kw,kf. (317208)
45 (pretest adj3 posttest).tw,kw,kf. (21915)
46 Historically Controlled Study/ (253520)
47 (control* adj2 study).tw,kw,kf. (1013756)
48 Control Groups/ (124647)
49 (control* adj2 group?).tw,kw,kf. (1665003)
50 trial.ti. (1000703)
51 or/34-50 (4661092)
52 23 and 51 [PROSTATE CANCER - SCREENING - nRCTs] (17094)
53 (201206* or 201207* or 201208* or 201209* or 201210* or 201211* or 201212* or 2013* or 2014* or 2015* or 2016* or 2017* or 2018* or 2019* or 202*).dt. (12359612)
54 52 and 53 [nRCTs - UPDATE PERIOD] (2096)
55 33 or 54 [RCTS, nRCTs] (2493)
56 exp Animals/ not Humans/ (17882502)
57 55 not 56 [ANIMAL-ONLY REMOVED] (2469)
58 (comment or editorial or news or newspaper article or case reports).pt. (4577683)
59 (letter not (letter and randomized controlled trial)).pt. (2404517)
60 57 not (58 or 59) [OPINION PIECES REMOVED] (2365)
61 60 use medall [MEDLINE RECORDS] (2365)
62 exp prostate tumor/ (277538)
63 (prostat* adj3 (neoplas* or cancer* or carcinoma* or adenocarcinom* or adeno-carcinom* or tumour* or tumor* or malignan* or metasta* or angiosarcoma* or angio-sarcoma* or

sarcoma* or teratoma* or lymphoma* or blastoma* or microcytic* or leiomyosarcoma* or lump?)).tw,kw,kf. (430955)
64 (PIN and (prostat* or intraepithelial or neoplas*)).tw,kw,kf. (3545)
65 or/62-64 [PROSTATE CANCER] (490710)
66 mass screening/ (181952)
67 cancer screening/ (124050)
68 early cancer diagnosis/ (10863)
69 (screen* or detect*).tw,kw,kf. (7868673)
70 (identif* or recogni*).ti,kw,kf. (1022950)
71 ((early or earlier or earliest) adj5 (identif* or recogni*)).tw,kw,kf. (213030)
72 (case finding? or casefinding?).tw,kw,kf. (15112)
73 exp prostate tumor/di, pc [Diagnosis, Prevention & Control] (41622)
74 digital rectal examination/ (11506)
75 (rect* adj2 exam*).tw,kw,kf. (19482)
76 DRE.tw,kw,kf. (9337)
77 palpation/ (31804)
78 palpat*.tw,kw,kf. (47171)
79 transrectal ultrasonography/ (13648)
80 ((transrectal* or trans-rectal*) adj4 (ultrasonograph* or ultrasound* or ultra-sonograph* or ultra-sound*)).tw,kw,kf. (23238)
81 (TRUS or TRUSB).tw,kw,kf. (10744)
82 ((prostat* specific antigen* or PSA) adj3 (diagnos* or screen* or test* or elevat* or level? or score? or result?)).tw,kw,kf. (65060)
83 prostate specific antigen/ and (diagnostic test/ or laboratory test/) (1245)
84 or/66-83 [SCREENING] (8985868)
85 65 and 84 [PROSTATE CANCER - SCREENING] (168853)
86 exp randomized controlled trial/ or controlled clinical trial/ (1565252)
87 clinical trial/ (1591371)
88 exp "controlled clinical trial (topic)"/ (235883)
89 (randomi#ed or randomi#ation? or randomly or RCT or placebo*).tw,kw,kf. (3973714)
90 ((singl* or doubl* or trebl* or tripl*) adj (mask* or blind* or dumm*)).tw,kw,kf. (789920)
91 trial.ti. (1000703)
92 or/86-91 [RCT FILTER] (5503990)
93 85 and 92 [PROSTATE CANCER - SCREENING - RCTs] (24054)
94 (2019082* or 2019083* or 201909* or 201910* or 201911* or 201912* or 202*).dc. (5595954)
95 93 and 94 [RCTs - UPDATE PERIOD] (2165)
96 exp controlled clinical trial/ (1565252)
97 exp "controlled clinical trial (topic)"/ (235883)
98 (control* adj2 trial*).tw,kw,kf. (1455418)
99 (nonrandom* or non-random* or quasi-random* or quasi-experiment*).tw,kw,kf. (169212)
100 (nRCT or non-RCT).tw,kw,kf. (1134)
101 (control* adj3 ("before and after" or "before after")).tw,kw,kf. (13035)
102 time series analysis/ (32540)

103 time series.tw,kw,kf. (87003)
104 pretest posttest control group design/ (597)
105 (pre- adj3 post-).tw,kw,kf. (317208)
106 (pretest adj3 posttest).tw,kw,kf. (21915)
107 controlled study/ (8855076)
108 (control* adj2 stud$3).tw,kw,kf. (1177002)
109 control group/ (124532)
110 (control$ adj2 group$1).tw,kw,kf. (1665003)
111 or/96-110 [nRCT FILTER] (12403082)
112 85 and 111 [PROSTATE CANCER - SCREENING - nRCTs] (46532)
113 (201206* or 201207* or 201208* or 201209* or 201210* or 201211* or 201212* or 2013* or 2014* or 2015* or 2016* or 2017* or 2018* or 2019* or 202*).dc. (17116342)
114 112 and 113 [nRCTs - UPDATE PERIOD] (25230)
115 95 or 114 [RCTs, nRCTs] (25631)
116 exp animal/ or exp animal experimentation/ or exp animal model/ or exp animal experiment/ or nonhuman/ or exp vertebrate/ (58909407)
117 exp human/ or exp human experimentation/ or exp human experiment/ (46175154)
118 116 not 117 (12736192)
119 115 not 118 [ANIMAL-ONLY REMOVED] (24620)
120 editorial.pt. (1334532)
121 letter.pt. not (letter.pt. and randomized controlled trial/) (2404416)
122 case report/ or exp case study/ (5194170)
123 119 not (120 or 121 or 122) [OPINION PIECES REMOVED] (24175)
124 conference abstract.pt. (4425980)
125 123 not 124 [CONFERENCE ABSTRACTS REMOVED] (15529)
126 125 use emczd [EMBASE RECORDS] (15529)
127 exp Prostatic Neoplasms/ (426243)
128 (prostat* adj3 (neoplas* or cancer* or carcinoma* or adenocarcinom* or adeno-carcinom* or tumour* or tumor* or malignan* or metasta* or angiosarcoma* or angio-sarcoma* or sarcoma* or teratoma* or lymphoma* or blastoma* or microcytic* or leiomyosarcoma* or lump?)).ti,ab,kw. (424902)
129 (PIN and (prostat* or intraepithelial or neoplas*)).ti,ab,kw. (3505)
130 or/127-129 [PROSTATE CANCER] (510802)
131 Mass Screening/ (181952)
132 "Early Detection of Cancer"/ (44407)
133 (screen* or detect*).ti,ab,kw. (7844637)
134 (identif* or recogni*).ti. (964728)
135 ((early or earlier or earliest) adj5 (identif* or recogni*)).ti,ab,kw. (212615)
136 (case finding? or casefinding?).ti,ab,kw. (14989)
137 exp Prostatic Neoplasms/di, pc [Diagnosis, Prevention & Control] (65584)
138 Prostatic Intraepithelial Neoplasia/di, pc [Diagnosis, Prevention & Control] (853)
139 Digital Rectal Examination/ (11506)
140 (rect* adj2 exam*).ti,ab,kw. (19320)
141 DRE.ti,ab,kw. (9253)

142 Palpation/ (31804)
143 palpat*.ti,ab,kw. (47122)
144 ((transrectal* or trans-rectal*) adj4 (ultrasonograph* or ultrasound* or ultra-sonograph* or ultra-sound*)).ti,ab,kw. (22981)
145 (TRUS or TRUSB).ti,ab,kw. (10658)
146 ((prostat* specific antigen* or PSA) adj3 (diagnos* or screen* or test* or elevat* or level? or score? or result?)).ti,ab,kw. (64967)
147 Prostate-Specific Antigen/ and (Diagnostic Tests, Routine/ or "Diagnostic Techniques and Procedures"/) (1065)
148 or/131-147 [SCREENING] (8911509)
149 130 and 148 [PROSTATE CANCER - SCREENING] (180952)
150 (201908* or 201909* or 201910* or 201911* or 201912* or 202*).up. (42411458)
151 149 and 150 [RCTs - UPDATE PERIOD] (89509)
152 (conference abstract or journal conference abstract).pt. (4618054)
153 151 not 152 [CONFERENCE ABSTRACTS REMOVED] (84450)
154 153 use cctr [CENTRAL RECORDS] (3451)
155 61 or 126 or 154 [ALL DATABASES] (21345)
156 33 use medall [MEDLINE RCTS] (734)
157 156 not 56 [ANIMAL-ONLY REMOVED] (728)
158 157 not (58 or 59) [OPINION PIECES REMOVED] (708)
159 95 use emczd [EMBASE RCTS] (2165)
160 159 not 118 [ANIMAL-ONLY REMOVED] (2147)
161 160 not (120 or 121 or 122) [OPINION PIECES REMOVED] (2040)
162 161 not 124 [CONFERENCE ABSTRACTS REMOVED] (1242)
163 158 or 162 or 154 [ALL DATABASES - RCTS] (5401)
164 remove duplicates from 163 (4436) [**TOTAL UNIQUE RCTS**]
165 164 use medall [MEDLINE UNIQUE RCTS] (705)
166 164 use emczd [EMBASE UNIQUE RCTS] (660)
167 164 use cctr [CENTRAL UNIQUE RECORDS] (3071)
168 54 use medall [MEDLINE NRCTS] (2096)
169 168 not 56 [ANIMAL-ONLY REMOVED] (2076)
170 169 not (58 or 59) [OPINION PIECES REMOVED] (1981)
171 114 use emczd [EMBASE NRCTS] (25230)
172 171 not 118 [ANIMAL-ONLY REMOVED] (24222)
173 172 not (120 or 121 or 122) [OPINION PIECES REMOVED] (23828)
174 173 not 124 [CONFERENCE ABSTRACTS REMOVED] (15214)
175 170 or 174 [ALL DATABASES - NRCTS] (17195)
176 175 not 163 [OVERLAP WITH RCTS REMOVED] (15944)
177 limit 176 to yr="2020-current" (4471)
178 remove duplicates from 177 (4293)
179 limit 176 to yr="2017-2019" (4818)
180 remove duplicates from 179 (4412)
181 limit 176 to yr="2014-2016" (4425)
182 remove duplicates from 181 (3937)

183 176 not (177 or 179 or 181) (2230)
184 remove duplicates from 183 (2022)
185 178 or 180 or 182 or 184 [**TOTAL UNIQUE nRCTs**] (14664)
186 185 use medall [MEDLINE UNIQUE nRCTs] (1656)
187 185 use emczd [EMBASE UNIQUE nRCTs] (13008)
188 164 or 185 [**TOTAL UNIQUE RCTS AND NRCTS**] (19100)

Supplementary File S4: List of grey literature sources

Eligibility criteria: Articles from 2019 to July 2022

Key words: PSA, DRE, PSA Screening

Organization	Date searched	Website Link
**Relevant websites**		
Best Practice Advocacy Centre New Zealand (bpacNZ)	20-Jul-22	https://bpac.org.nz/Default.aspx
Prostate Cancer Foundation of Australia (PCFA)	20-Jul-22	https://www.prostate.org.au/
The Royal College of Pathologists of Australasia (RCPA)	22-Jul-22	https://www.rcpa.edu.au/
Cancer Council Australia	22-Jul-22	https://www.cancer.org.au/
Urological Society of Australia and New Zealand (USANZ)	26-Jul-22	https://www.usanz.org.au/
Royal Australian College of General Practitioners (RACGP)	26-Jul-22	https://www.racgp.org.au/
Health Quality Ontario (HQO)	27-Jul-22	https://www.hqontario.ca
Institut national d’excellence en santé et en services sociaux	27-Jul-22	https://www.inesss.qc.ca

Supplementary File S5: Data Extraction Items

Study identification

1. Author
2. Year of publication
3. Name of journal/source document
4. Study funding source
5. Country

Study characteristics

6. Type of study/study design
    a. Randomized controlled trial
    b. Quasi-randomized study
    c. Non-randomized experimental
    d. Cohort study
    e. Case-control study
    f. Cross-sectional study
    g. Systematic review
    h. Qualitative study
    i. Descriptive study
7. Randomization method (if applicable)
8. Study aim/objective
9. Study start and end date
10. Trial (e.g., European Randomized Study of Screening for Prostate Cancer), if applicable
11. Number of centres (if applicable)
12. Duration of follow-up
13. Protocol deviations (if applicable)

Population characteristics

14. Total number of participants (sample size)
15. Recruitment method
16. eligibility criteria for participants
17. Response rate
18. Population age
19. Special populations (e.g., ethnic groups, LGBTQ+, disability)
    a. information regarding respondent bias/representativeness of the included population
20. number enrolled, assigned to each arm, received intended screening, analyzed, and losses and exclusions after randomization

Methods (details about intervention/exposure)

21. type of screening test performed
22. screening program details (e.g., screening interval, universal/targeted, coverage, setting)
23. type of additional testing (KQ2)
24. follow-up tests, biopsy, procedures, care, or treatment
25. Statistical methods and additional analysis (adjusted analyses, secondary analyses, missing data)

Outcome data

26. Relevant outcomes evaluated (n/%, SD, OR, RR, HR, etc.)
27. Outcomes of interest
    a. Prostate Cancer Mortality
    b. All-cause mortality
    c. False positives
    d. Overdiagnosis
    e. Complications due to biopsy
    f. Quality of life or functioning (overall and disease-specific; using validated scales)
    g. Incontinence (urinary or bowel)
    h. Erectile dysfunction
    i. Incidence of metastatic cancer
    j. Psychological Effects

Study findings

28. Measures of effect
29. Overall study conclusions
30. Reported limitations
31. Additional comments

Supplementary File S6: List of excluded studies with reasons

Ineligible study design (n=291)

1. Finne, P., Finne, R., Auvinen, A., Juusela, H., Aro, J., auml, auml;tt, auml;nen, L., Hakama, M., Rannikko, S., Tammela, T. L., Stenman, U.. Predicting the outcome of prostate biopsy in screen-positive men by a multilayer perceptron network. Urology. 2000. Vol.56:418-422p

2. Collin, S. M., Metcalfe, C., Donovan, J. L., Athene Lane, J., Davis, M., Neal, D. E., Hamdy, F. C., Martin, R. M.. Associations of sexual dysfunction symptoms with PSA-detected localised and advanced prostate cancer: a case-control study nested within the UK population-based ProtecT (Prostate testing for cancer and Treatment) study. European journal of cancer (Oxford, England :. 2009. Vol.45:3254-3261p

3. Etzioni, R., Falcon, S., Gann, P. H., Kooperberg, C. L., Penson, D. F., Stampfer, M. J.. Prostate-specific antigen and free prostate-specific antigen in the early detection of prostate cancer: do combination tests improve detection?. Cancer epidemiology, biomarkers & prevention. 2004. Vol.13:1640-1645p

4. Wald, N. J., Bestwick, J. P., Morris, J. K.. Multi-marker risk-based screening for prostate cancer. Journal of Medical Screening. 2022. 29:123-133

5. Preston, M. A., Gerke, T., Carlsson, S. V., Signorello, L., Sjoberg, D. D., Markt, S. C., Kibel, A. S., Trinh, Q. D., Steinwandel, M., Blot, W., Vickers, A. J., Lilja, H., Mucci, L. A., Wilson, K. M.. Baseline Prostate-specific Antigen Level in Midlife and Aggressive Prostate Cancer in Black Men. European Urology. 2019. 75:399-407

6. Ankerst, D. P., Liss, M., Zapata, D., Hoefler, J., Thompson, I. M., Leach, R. J.. A case control study of sarcosine as an early prostate cancer detection biomarker. BMC Urology. 2015. 15:99

7. Bancroft, E. K., Page, E. C., Castro, E., Lilja, H., Vickers, A., Sjoberg, D., Assel, M., Foster, C. S., Mitchell, G., Drew, K., Maehle, L., Axcrona, K., Evans, D. G., Bulman, B., Eccles, D., McBride, D., van Asperen, C., Vasen, H., Kiemeney, L. A., Ringelberg, J., Cybulski, C., Wokolorczyk, D., Selkirk, C., Hulick, P. J., Bojesen, A., Skytte, A. B., Lam, J., Taylor, L., Oldenburg, R., Cremers, R., Verhaegh, G., van Zelst-Stams, W. A., Oosterwijk, J. C., Blanco, I., Salinas, M., Cook, J., Rosario, D. J., Buys, S., Conner, T., Ausems, M. G., Ong, K. R., Hoffman, J., Domchek, S., Powers, J., Teixeira, M. R., Maia, S., Foulkes, W. D., Taherian, N., Ruijs, M., Helderman-van den Enden, A. T., Izatt, L., Davidson, R., Adank, M. A., Walker, L., Schmutzler, R., Tucker, K., Kirk, J., Hodgson, S., Harris, M., Douglas, F., Lindeman, G. J., Zgajnar, J., Tischkowitz, M., Clowes, V. E., Susman, R., Ramon y Cajal, T., Patcher, N., Gadea, N., Spigelman, A., van Os, T., Liljegren, A., Side, L., Brewer, C., Brady, A. F., Donaldson, A., Stefansdottir, V., Friedman, E., Chen-Shtoyerman, R., Amor, D. J., Copakova, L., Barwell, J., Giri, V. N., Murthy, V., Nicolai, N., Teo, S. H., Greenhalgh, L., Strom, S., Henderson, A., McGrath, J., Gallagher, D., Aaronson, N., Ardern-Jones, A., Bangma, C., Dearnaley, D., Costello,

P., Eyfjord, J., Rothwell, J., Falconer, A., Gronberg, H., Hamdy, F. C., Johannsson, O., Khoo, V., Kote-Jarai, Z., Lubinski, J., Axcrona, U., Melia, J., McKinley, J., Mitra, A. V., Moynihan, C., Rennert, G., Suri, M., Wilson, P., Killick, E., Collaborators, Impact, Moss, S., Eeles, R. A.. Targeted prostate cancer screening in BRCA1 and BRCA2 mutation carriers: results from the initial screening round of the IMPACT study. European Urology. 2014. 66:489-99

8. Larsen, S. B., Brasso, K., Iversen, P., Christensen, J., Christiansen, M., Carlsson, S., Lilja, H., Friis, S., Tjonneland, A., Dalton, S. O.. Baseline prostate-specific antigen measurements and subsequent prostate cancer risk in the Danish Diet, Cancer and Health cohort. European Journal of Cancer. 2013. 49:3041-8

9. Vickers, A. J., Ulmert, D., Sjoberg, D. D., Bennette, C. J., Bjork, T., Gerdtsson, A., Manjer, J., Nilsson, P. M., Dahlin, A., Bjartell, A., Scardino, P. T., Lilja, H.. Strategy for detection of prostate cancer based on relation between prostate specific antigen at age 40-55 and long term risk of metastasis: case-control study. BMJ. 2013. 346:f2023

10. Hill, O. T., Mason, T. J., Schwartz, S. W., Foulis, P. R.. Improving prostate cancer detection in veterans through the development of a clinical decision rule for prostate biopsy. BMC Urology. 2013. 13:6

11. Rundle, A., Neckerman, K. M., Sheehan, D., Jankowski, M., Kryvenko, O. N., Tang, D., Rybicki, B. A.. A prospective study of socioeconomic status, prostate cancer screening and incidence among men at high risk for prostate cancer. Cancer Causes & Control. 2013. 24:297-303

12. Mkanta, W. N., Ndjakani, Y., Bandiera, F. C., Blumenthal, D. S., Nseyo, U. O., Asal, N. R.. Prostate Cancer Screening and Mortality in Blacks and Whites: A Hospital-based Case-Control Study. Journal of the National Medical Association. 2015. 107:32-8

13. Lee, J., Yang, S. W., Jin, L., Lee, C. L., Lee, J. Y., Shin, J. H., Lim, J. S., Song, K. H.. Is PSA density of the peripheral zone as a useful predictor for prostate cancer in patients with gray zone PSA levels?. BMC Cancer. 2021. 21(1) (no pagination):#pages#

14. Rybicki, B. A., Kryvenko, O. N., Wang, Y., Jankowski, M., Trudeau, S., Chitale, D. A., Gupta, N. S., Rundle, A., Tang, D.. Racial differences in the relationship between clinical prostatitis, presence of inflammation in benign prostate and subsequent risk of prostate cancer. Prostate Cancer and Prostatic Diseases. 2016. 19(2):145-150

15. Van Leeuwen, P. J., Otto, S. J., Kranse, R., Roobol, M. J., Bul, M., Zhu, X., De Koning, H., Schroder, F. H.. Increased non-prostate cancer death risk in clinically diagnosed prostate cancer. BJU International. 2012. 110(2):188-194
16. Spooner, J., Birk, M., Espinosa, V., Hoag, N.. The times they are a-changin': The evolution of prostate cancer screening practices and beliefs among primary care

physicians in Victoria, British Columbia. Canadian Urological Association Journal. 2021. 15:192-196

17. Catalona, W. J., Richie, J. P., Ahmann, F. R., Hudson, M. A., Scardino, P. T., Flanigan, R. C., DeKernion, J. B., Ratliff, T. L., Kavoussi, L. R., Dalkin, B. L., Waters, W. B., MacFarlane, M. T., Southwick, P. C.. Comparison of digital rectal examination and serum prostate specific antigen in the early detection of prostate cancer: results of a multicenter clinical trial of 6,630 men. Journal of urology. 1994. Vol.151:1283-1290p

18. Burgess, L., Aldrighetti, C. M., Ghosh, A., Niemierko, A., Chino, F., Huynh, M. J., Efstathiou, J. A., Kamran, S. C.. Association of the USPSTF Grade D Recommendation Against Prostate-Specific Antigen Screening With Prostate Cancer-Specific Mortality. JAMA Network Open. 2022. 5:e2211869

19. Makau-Barasa, L. K., Manirakiza, A., Carvalho, A. L., Rebbeck, T. R.. Prostate Cancer Screening, Diagnostic, Treatment Procedures and Costs in Sub-Saharan Africa: A Situational Analysis. Cancer Control. 2022. 29:#pages#

20. Braga, R., Costa, A. R., Pina, F., Moura-Ferreira, P., Lunet, N.. Prostate cancer screening in Portugal: Prevalence and perception of potential benefits and adverse effects. European Journal of Cancer Prevention. 2020. #volume#:248-251

21. Harrison, S., Tilling, K., Turner, E. L., Lane, J. A., Simpkin, A., Davis, M., Donovan, J., Hamdy, F. C., Neal, D. E., Martin, R. M.. Investigating the prostate specific antigen, body mass index and age relationship: is an age-BMI-adjusted PSA model clinically useful?. Cancer Causes and Control. 2016. 27(12):1465-1474

22. Drummond, F. J., O'Leary, E., Gavin, A., Kinnear, H., Sharp, L.. Mode of prostate cancer detection is associated with the psychological wellbeing of survivors: results from the PiCTure study. Supportive Care in Cancer. 2016. 24(5):2297-2307

23. Prajsner, A., Chudek, J., Szybalska, A., Piotrowicz, K., Zejda, J., Wiecek, A.. Socioeconomic determinants of prostate-specifc antigen testing and estimation of the prevalence of undiagnosed prostate cancer in an elderly Polish population based on the PolSenior study. Archives of Medical Science. 2016. 12(5):1028-1035

24. Shirazi, M., Ariafar, A., Zeyghami, S., Hosseini, M. M., Khezri, A. A.. Association of diet with prostate specific antigen and prostate volume. Nephro-Urology Monthly. 2014. 6(4) (no pagination):#pages#

25. Kotwal, A. A., Mohile, S. G., Dale, W.. Remaining life expectancy measurement and PSA screening of older men. Journal of Geriatric Oncology. 2012. 3(3):196-204

26. Bandala-Jacques, A., Castellanos Esquivel, K. D., Perez-Hurtado, F., Hernandez-Silva, C., Reynoso-Noveron, N.. Prostate Cancer Risk Calculators for Healthy Populations: Systematic Review. JMIR Cancer. 2021. 7:e30430

27. Harland, N., Stenzl, A., Todenhofer, T.. Role of Multiparametric Magnetic Resonance Imaging in Predicting Pathologic Outcomes in Prostate Cancer. The World Journal of Mens Health. 2021. 39:38-47

28. Paschen, U., Sturtz, S., Fleer, D., Lampert, U., Skoetz, N., Dahm, P.. Assessment of prostate-specific antigen screening: an evidence-based report by the German Institute for Quality and Efficiency in Health Care. BJU International. 2022. 129:280-289

29. Kim, A., Chung, K. C., Keir, C., Patrick, D. L.. Patient-reported outcomes associated with cancer screening: a systematic review. BMC Cancer. 2022. 22:223

30. Tan, G. H., Nason, G., Ajib, K., Woon, D. T. S., Herrera-Caceres, J., Alhunaidi, O., Perlis, N.. Smarter screening for prostate cancer. World journal of urology. 2019. 37(6):991-999

31. Neal, D. E., Donovan, J. L.. Prostate cancer: to screen or not to screen?. Lancet oncology. 2000. Vol.1:17-24p

32. Clements, M. B., Abdalla, B., Culp, S. H., Costabile, R. A., Krupski, T. L.. Prostate Cancer Characteristics in the US Preventive Services Task Force Grade D Era: A Single-Center Study and Meta-Analysis. Urologia Internationalis. 2020. 104:692-698

33. Fenton, J. J., Weyrich, M. S., Durbin, S., Liu, Y., Bang, H., Melnikow, J.. Prostate-Specific Antigen-Based Screening for Prostate Cancer: Evidence Report and Systematic Review for the US Preventive Services Task Force. JAMA. 2018. 319:1914-1931

34. Rahal, A. K., Badgett, R. G., Hoffman, R. M.. Screening Coverage Needed to Reduce Mortality from Prostate Cancer: A Living Systematic Review. PLoS ONE [Electronic Resource]. 2016. 11:e0153417

35. Carter, J. L., Coletti, R. J., Harris, R. P.. Quantifying and monitoring overdiagnosis in cancer screening: a systematic review of methods. BMJ. 2015. 350:g7773

36. Lee, Y. J., Park, J. E., Jeon, B. R., Lee, S. M., Kim, S. Y., Lee, Y. K.. Is prostate-specific antigen effective for population screening of prostate cancer? A systematic review. Annals of Laboratory Medicine. 2013. 33:233-41

37. Carter, H. B., Albertsen, P. C., Barry, M. J., Etzioni, R., Freedland, S. J., Greene, K. L., Holmberg, L., Kantoff, P., Konety, B. R., Murad, M. H., Penson, D. F., Zietman, A. L.. Early detection of prostate cancer: AUA Guideline. Journal of Urology. 2013. 190:419-26

38. Ilic, D., Neuberger, M. M., Djulbegovic, M., Dahm, P.. Screening for prostate cancer. Cochrane Database of Systematic Reviews. 2013. #volume#:CD004720

39. Henning, G. M., Andriole, G. L., Kim, E. H.. Liquid biomarkers for early detection of prostate cancer and summary of available data for their use in African-American men. Prostate Cancer and Prostatic Diseases.. 2022. #volume#:#pages#

40. Green, A., Tait, C., Aboumarzouk, O., Somani, B. K., Cohen, N. P.. There are calls for a national screening programme for prostate cancer: What is the evidence to justify such a national screening programme?. Scottish Medical Journal. 2013. 58(2):64-68

41. DeFrank, J. T., Barclay, C., Sheridan, S., Brewer, N. T., Gilliam, M., Moon, A. M., Rearick, W., Ziemer, C., Harris, R.. The Psychological Harms of Screening: the Evidence We Have Versus the Evidence We Need. Journal of General Internal Medicine. 2015. 30(2):242-248

42. Hayes, J. H., Barry, M. J.. Screening for prostate cancer with the prostate-specific antigen test: A review of current evidence. JAMA - Journal of the American Medical Association. 2014. 311(11):1143-1149

43. Ilic, D., Djulbegovic, M., Jung, J. H., Hwang, E. C., Zhou, Q., Cleves, A., Agoritsas, T., Dahm, P.. Prostate cancer screening with prostate-specific antigen (PSA) test: A systematic review and meta-analysis. BMJ (Online). 2018. 362 (no pagination):#pages#

44. Deng, T., Zhang, M., Feng, S., Duan, X., Zhang, T., Cai, C., Lan, Y., Wu, W., Zeng, G.. Number of screening rounds and risk of prostate cancer: A systematic review and meta-analysis. International Journal of Clinical and Experimental Medicine. 2018. 11(1):1-11

45. Dong, D. X., Ji, Z. G.. Current progress and controversies in prostate cancer management. Chinese Medical Journal. 2017. 130(24):2991-2995

46. Chad-Friedman, E., Coleman, S., Traeger, L. N., Pirl, W. F., Goldman, R., Atlas, S. J., Park, E. R.. Psychological distress associated with cancer screening: A systematic review. Cancer. 2017. 123(20):3882-3894

47. Pron, G.. Prostate-specific antigen (Psa)-based population screening for prostate cancer: An evidence-based analysis. Ontario Health Technology Assessment Series. 2015. 15:#pages#

48. Loeb, S., Bjurlin, M. A., Nicholson, J., Tammela, T. L., Penson, D. F., Carter, H. B., Carroll, P., Etzioni, R.. Overdiagnosis and overtreatment of prostate cancer. European Urology. 2014. 65(6):1046-1055

49. Wallner, L. P., Jacobsen, S. J.. Prostate-specific antigen and prostate cancer mortality: A systematic review. American Journal of Preventive Medicine. 2013. 45(3):318-326

50. Kotwal, A. A., Walter, L. C.. Cancer Screening Among Older Adults: a Geriatrician's Perspective on Breast, Cervical, Colon, Prostate, and Lung Cancer Screening. Current Oncology Reports. 2020. 22:108

51. Raoof, S., Lee, R. J., Jajoo, K., Mancias, J. D., Rebbeck, T. R., Skates, S. J.. Multi-cancer early detection technologies: a review informed by past cancer screening studies. Cancer epidemiology, biomarkers & prevention : a publication of the American Association for Cancer Research, cosponsored by the American Society of Preventive Oncology.. 2022. 23:#pages#

52. Grubb, R. L.. Prostate Cancer: Update on Early Detection and New Biomarkers. Missouri medicine. 2018. 115(2):132-134

53. Djavan, B., Eckersberger, E., Finkelstein, J., Sadri, H., Taneja, S. S., Lepor, H.. Prostate-specific Antigen Testing and Prostate Cancer Screening. Primary Care - Clinics in Office Practice. 2010. 37(3):441-459

54. He, B. M., Chen, R., Sun, T. Q., Yang, Y., Zhang, C. L., Ren, S. C., Gao, X., Sun, Y. H.. Prostate cancer risk prediction models in Eastern Asian populations: Current status, racial difference, and future directions. Asian Journal of Andrology. 2020. 22(2):158-161

55. Ito, K., Oki, R., Sekine, Y., Arai, S., Miyazawa, Y., Shibata, Y., Suzuki, K., Kurosawa, I.. Screening for prostate cancer: History, evidence, controversies and future perspectives toward individualized screening. International Journal of Urology. 2019. 26(10):956-970

56. Cupp, M. R., Oesterling, J. E.. Prostate-Specific Antigen, Digital Rectal Examination, and Transrectal Ultrasonography: Their Roles in Diagnosing Early Prostate Cancer. Mayo Clinic Proceedings. 1993. 68(3):297-306

57. Heijnsdijk, E. A. M., Bangma, C. H., Borras, J. M., de Carvalho, T. M., Castells, X., Eklund, M., Espinas, J. A., Graefen, M., Gronberg, H., Lansdorp-Vogelaar, I., Leeuwen, P. J. V., Nelen, V., Recker, F., Roobol, M. J., Vandenbulcke, P., de Koning, H. J.. Summary statement on screening for prostate cancer in Europe. International journal of cancer. 2018. Vol.142:741-746p

58. Schroder, F. H.. Detection of prostate cancer: the impact of the European Randomized Study of Screening for Prostate Cancer (ERSPC). Canadian journal of urology. 2005. 12:2-6

59. Bangma, C. H., Rietbergen, J. B., Schr, ouml;der, F. H.. Prostate-specific antigen as a screening test. The Netherlands experience. Urologic clinics of North America. 1997. Vol.24:307-314p

60. Aizer, A. A., D'Amico, A. V.. Should all colorectal cancer patients over age 60 be screened for prostate cancer?. Oncology. 2013. 27:#pages#

61. Finne, P., Stenman, U. H., auml, auml;tt, auml;nen, L., auml;kinen, T., Tammela, T. L., Martikainen, P., Ruutu, M., Ala-Opas, M., Aro, J., Karhunen, P. J., Lahtela, J., Rissanen, P., Juusela, H., Hakama, M., Auvinen, A.. The Finnish trial of prostate cancer screening: where are we now?. BJU international. 2003. 92:22-26

62. Austin, O. J.. Prostate-Specific Antigen Prostate Cancer Screening: answers to the Critical Questions. Annals of long term care. 2012. Vol.20:16-21p

63. Becher, E., Wang, A., Lepor, H.. Prostate Cancer Screening and Management in Solid Organ Transplant Candidates and Recipients. Reviews in Urology. 2019. 21:85-92

64. Hugosson, J.. Stopping screening, when and how?. Translational Andrology & Urology. 2018. 7:46-53

65. Gohagan, J. K., Prorok, P. C., Greenwald, P., Kramer, B. S.. The PLCO Cancer Screening Trial: Background, Goals, Organization, Operations, Results. Reviews on Recent Clinical Trials. 2015. 10:173-80

66. Andriole, G. L.. Update of the Prostate, Lung, Colorectal, and Ovarian Cancer Screening Trial. Recent Results in Cancer Research. 2014. 202:53-7

67. Punnen, S., Pavan, N., Parekh, D. J.. Finding the Wolf in Sheep's Clothing: The 4Kscore Is a Novel Blood Test That Can Accurately Identify the Risk of Aggressive Prostate Cancer. Reviews in Urology. 2015. 17:3-13

68. Moul, J. W.. Screening for prostate cancer in african americans. Current Urology Reports. 2000. 1(1):57-64

69. Zhang, K., Bangma, C. H., Roobol, M. J.. Prostate cancer screening in Europe and Asia. Asian Journal of Urology. 2017. 4(2):86-95

70. Becerra, M. F., Atluri, V. S., Bhattu, A. S., Punnen, S.. Serum and urine biomarkers for detecting clinically significant prostate cancer. Urologic Oncology: Seminars and Original Investigations. 2021. 39(10):686-690

71. Eapen, R. S., Herlemann, A., Washington, S. L., Cooperberg, M. R.. Impact of the United States Preventive Services Task Force 'D' recommendation on prostate cancer screening and staging. Current Opinion in Urology. 2017. 27(3):205-209

72. Carlsson, S. V., Roobol, M. J.. Improving the evaluation and diagnosis of clinically significant prostate cancer in 2017. Current Opinion in Urology. 2017. 27(3):198-204

73. Lewis, R., Hornberger, B.. The current state of prostate-specific antigen testing. JAAPA : official journal of the American Academy of Physician Assistants. 2016. 29(9):51-53

74. Mulhem, E., Fulbright, N., Duncan, N.. Prostate cancer screening. American Family Physician. 2015. 92(8):683-688

75. Alberts, A. R., Schoots, I. G., Roobol, M. J.. Prostate-specific antigen-based prostate cancer screening: Past and future. International Journal of Urology. 2015. 22(6):524-532

76. Morgan, T., Palapattu, G., Wei, J.. Screening for Prostate Cancer-Beyond Total PSA, Utilization of Novel Biomarkers. Current Urology Reports. 2015. 16(9) (no pagination):#pages#

77. Quaas, J.. PSA screening for prostate cancer. American Family Physician. 2015. 91(9):606A-606B

78. Tabayoyong, W., Abouassaly, R.. Prostate Cancer Screening and the Associated Controversy. Surgical Clinics of North America. 2015. 95(5):1023-1039

79. Richardson, A. K., Potter, J. D.. Screening for colorectal cancer and prostate cancer: Challenges for New Zealand. New Zealand Medical Journal. 2014. 127(1395):23-30

80. Caruana, M., Kang, Y. J., Smith, D., O'Connell, D., Canfell, K.. Estimating the benefits and harms of PSA testing in the Australian context. Cancer Forum. 2014. 38(3):221-223

81. Etzioni, R. D., Thompson, I. M.. What do the screening trials really tell us and where do we go from here?. Urologic Clinics of North America. 2014. 41(2):223-228

82. Roobol, M. J., Carlsson, S. V.. Risk stratification in prostate cancer screening. Nature Reviews Urology. 2013. 10(1):38-48

83. Castro, E., Goh, C. L., Eeles, R. A.. Prostate cancer screening in BRCA and Lynch syndrome mutation carriers. American Society of Clinical Oncology educational book / ASCO. American Society of Clinical Oncology. Meeting.. 2013. #volume#:#pages#

84. Borza, T., Konijeti, R., Kibel, A. S.. Early Detection, PSA Screening, and Management of Overdiagnosis. Hematology/Oncology Clinics of North America. 2013. 27(6):1091-1110

85. Vellekoop, A., Loeb, S.. The Utility of Prostate-Specific Antigen Screening and Prostate Cancer Treatment in Elderly Patients. Current Translational Geriatrics and Gerontology Reports. 2013. 2(2):51-57

86. Bretthauer, M., Hoff, G.. Comparative effectiveness research in cancer screening programmes. BMJ (Online). 2012. 344(7860) (no pagination):#pages#

87. Killick, E., Bancroft, E., Kote-Jarai, Z., Eeles, R.. Beyond Prostate-specific Antigen - Future Biomarkers for the Early Detection and Management of Prostate Cancer. Clinical Oncology. 2012. 24(8):545-555

88. Rove, K. O., Crawford, E. D.. Randomized controlled screening trials for prostate cancer using prostate-specific antigen: A tale of contrasts. World Journal of Urology. 2012. 30(2):137-142

89. Smith, D. W., Stoimenova, D., Eid, K., Barqawi, A.. The role of targeted focal therapy in the management of low-risk prostate cancer: Update on current challenges. Prostate Cancer. 2012. (no pagination):#pages#

90. Anonymous. PSA-based screening for prostate cancer: Too many adverse effects. Prescrire International. 2012. 21(130):215-217

91. Turkbey, B.. Beyond the AJR: "Comparison of Multiparametric Magnetic Resonance Imaging-Targeted Biopsy with Systematic Transrectal Ultrasonography Biopsy for Biopsy-Naive Men at Risk for Prostate Cancer A Phase 3 Randomized Clinical Trial". AJR. American Journal of Roentgenology. 2021. 07:07

92. Raslan, M., Mercader, C., Lamb, A. D.. Re: Prostate Cancer Screening Using a Combination of Risk-prediction, MRI, and Targeted Prostate Biopsies (STHLM3-MRI): A Prospective, Population-based, Randomised, Open-label, Non-inferiority Trial. European Urology. 2022. 81:543-544

93. Dalela, D., Sood, A., Keeley, J., Rogers, C., Menon, M., Abdollah, F.. Generalizability of Prostate-Specific Antigen (PSA) Screening Trials in a "Real World" Setting: A Nationwide Survey Analysis. Urology. 2021. 148:1-3

94. Shoag, J. E., Nyame, Y. A., Gulati, R., Etzioni, R., Hu, J. C.. Reconsidering the Trade-offs of Prostate Cancer Screening. New England Journal of Medicine. 2020. 382:2465-2468

95. Hackshaw, A., Berg, C. D.. An efficient randomised trial design for multi-cancer screening blood tests: nested enhanced mortality outcomes of screening trial. The Lancet Oncology. 2021. 22(10):1360-1362

96. Bratt, O., Lilja, H.. Modern prostate cancer diagnostics reduce overdiagnosis-will they open up for population-based screening?. Scandinavian Journal of Urology. 2021. 55(6):491-492

97. Albertsen, P. C.. Prostate cancer screening: a new way forward or another false start?. Nature Reviews Urology. 2021. 18(10):579-580

98. Martin, R. M., Dixon, P., Turner, E., Keeney, E.. Contribution of the Cluster randomised triAl of PSA testing for Prostate cancer (CAP) to the ongoing debate on the value of prostate cancer screening. BJU international. 2022. Vol.129:269-270p

99. Martin, R. M., Donovan, J. L., Turner, E. L., Metcalfe, C., Young, G. J., Walsh, E. I., Lane, J. A., Noble, S., Oliver, S. E., Evans, S., Sterne, J. A. C., Holding, P., Ben-Shlomo, Y., Brindle, P., Williams, N. J., Hill, E. M., Ng, S. Y., Toole, J., Tazewell, M. K., Hughes, L. J., Davies, C. F., Thorn, J. C., Down, E., Davey Smith, G., Neal, D. E., Hamdy, F. C., Group, C. A. P. Trial. Effect of a Low-Intensity PSA-Based Screening Intervention on Prostate Cancer Mortality: the CAP Randomized Clinical Trial. Jama. 2018. Vol.319:883-895p

100. Gulati, R., Albertsen, P. C.. Insights from the PLCO trial about prostate cancer screening. Cancer. 2016. #volume#:07191-8667

101. Taneja, S. S.. Re: lethal Prostate Cancer in the PLCO Cancer Screening Trial. Journal of urology. 2016. #volume#:#pages#

102. Ruffin, M. T.. Screening for prostate cancer. Journal of family practice. 1999. Vol.48:581-582p

103. Harwood, R. H., Catalona, W. J.. Screening for prostate cancer (multiple letters). British medical journal. 1997. Vol.315:186-187p

104. Richardson, A.. Prostate cancer screening: is it possible to explain diametrically opposed views?. New Zealand medical journal. 2005. Vol.118:U1289p

105. Donzelli, A., Castelluzzo, G., Giudicatti, G.. Re: jonas Hugosson, Monique J. Roobol, Marianne MÃ¥nsson, et al. A 16-yr Follow-up of the European Randomized Study of Screening for Prostate Cancer. Eur Urol 2019;76: 43-51: mortality in the Age Group â‰¥70â¯yr and the Case of Italy. European urology. 2019. #volume#:#pages#

106. Taneja, S. S.. Re: effect of a Low-Intensity PSA-Based Screening Intervention on Prostate Cancer Mortality: the CAP Randomized Clinical Trial. Journal of urology. 2018. Vol.200:699-p

107. Boer, R.. Quebec randomized controlled trial on prostate cancer screening shows no evidence for mortality reduction. Prostate. 1999. Vol.40:130-134p

108. Pinsky, P. F.. Results of a randomized controlled trail of prostate cancer screening. Prostate. 2004. Vol.61:371p

109. Catalona, W. J., D'Amico, A. V., Fitzgibbons, W. F., Kosoko-Lasaki, O., Leslie, S. W., Lynch, H. T., Moul, J. W., Rendell, M. S., Walsh, P. C.. What the U.S. preventive services task force missed in its prostate cancer screening recommendation. Annals of Internal Medicine. 2012. Vol.157:1-3p

110. Loeb, S., Trock, B. J.. Re: randomised prostate cancer screening trial: 20 year follow-up. European urology. 2011. Vol.60:1306-1307p

111. Zhu, X., Van Leeuwen, P. J., Bul, M., Otto, S. J., De Koning, H. J., Bangma, C. H., Schroder, F. H., Roobol, M. J.. Re: disease-specific survival of men with prostate cancer detected during the screening interval: results of the european randomized study of screening for prostate cancerrotterdam after 11 years of follow-up. Journal of urology. 2011. Vol.186:1874p

112. Linton, K. D., Catto, J. W. F.. Re: comorbidity and mortality results from a randomised prostate cancer screening trial. European urology. 2011. Vol.60:867p

113. Van Rij, S., Murphy, D. G.. Re: screening and prostate cancer mortality: results of the European randomised study of screening for prostate cancer (ERSPC) at 13 years of follow-up. European urology. 2014. Vol.66:1187-1188p

114. Kim, E. H., Andriole, G. L.. Prostate-specific antigen-based screening: controversy and guidelines. BMC medicine. 2015. 13:#pages#

115. Ilic, D., Philipp, D.. Randomised controlled trial: although the evidence is not clear, decreases in prostate cancer mortality in specific subgroups of men may be due to screening. Evidence-based medicine. 2015. Vol.20:102p

116. Walsh, P. C.. Re.: interval cancers in the Antwerp European Randomised Study of Screening for Prostate Cancer study, using a 6 year screening interval. Journal of urology. 2011. Vol.185:1707p

117. Kallidonis, P., Liatsikos, E.. Re: long-term Results of Active Surveillance in the Goteborg Randomized, Population-based Prostate Cancer Screening Trial. European urology. 2017. #volume#:#pages#

118. Pruthi, D. K., Ankerst, D. P., Liss, M. A.. Novel Definitions of Low-risk and High-risk Prostate Cancer: implications for the European Randomized Study of Screening for Prostate Cancer Risk Assessment Tool. European urology. 2017. #volume#:#pages#

119. Bolla, M., Van Poppel, H.. Prostate cancer. An insight into screening and local treatment. European journal of cancer (Oxford, England :. 1996. Vol.32A:1434-1435p

120. Bagcchi, S.. PSA testing beneficial for prostate cancer. The lancet. 2014. Oncology. Vol.15:e424p

121. Shoag, J., Halpern, J., Eisner, B., Lee, R., Mittal, S., Barbieri, C. E., Shoag, D.. Efficacy of Prostate-Specific Antigen Screening: use of Regression Discontinuity in the PLCO Cancer Screening Trial. JAMA oncology. 2015. Vol.1:984-986p

122. van Leeuwen, P. J.. Prostate cancer screening has no effect on prostate cancer specific mortality over 20 years of follow-up of Swedish men. Evidence based medicine. 2012. Vol.17:25-26p

123. Hoffman, R. M.. Periodic PSA-based screening in men 55 to 69 years of age reduced prostate cancer mortality. Annals of Internal Medicine. 2012. 157(2):JC2-4

124. Moore, C.. Prostate-specific membrane antigen PET-CT before radical treatment. The Lancet. 2020. 395(10231):1170-1172

125. Fletcher, R. H.. Guideline: Experts recommend against prostate cancer screening with prostate-specific antigen test. Annals of Internal Medicine. 2019. 170(2):JC2

126. Kim, S. J., Vickers, A. J., Hu, J. C.. Challenges in Adopting Level 1 Evidence for Multiparametric Magnetic Resonance Imaging as a Biomarker for Prostate Cancer Screening. JAMA Oncology. 2018. 4(12):1663-1664

127. Mikropoulos, C., Selkirk, C. G. H., Saya, S., Bancroft, E., Vertosick, E., Dadaev, T., Brendler, C., Page, E., Dias, A., Evans, D. G., Rothwell, J., Maehle, L., Axcrona, K., Richardson, K., Eccles, D., Jensen, T., Osther, P. J., Van Asperen, C. J., Vasen, H., Kiemeney, L. A., Ringelberg, J., Cybulski, C., Wokolorczyk, D., Hart, R., Glover, W., Lam, J., Taylor, L., Salinas, M., Feliubadalo, L., Oldenburg, R., Cremers, R., Verhaegh, G., Van Zelst-Stams, W. A., Oosterwijk, J. C., Cook, J., Rosario, D. J., Buys, S. S., Conner, T., Domchek, S., Powers, J., Ausems, M. G., Teixeira, M. R., Maia, S., Izatt, L., Schmutzler, R., Rhiem, K., Foulkes, W. D., Boshari, T., Davidson, R., Ruijs, M., Helderman-Van Den Enden, A. T., Andrews, L., Walker, L., Snape, K., Henderson, A., Jobson, I., Lindeman, G. J., Liljegren, A., Harris, M., Adank, M. A., Kirk, J., Taylor, A., Susman, R., Chen-Shtoyerman, R., Pachter, N., Spigelman, A., Side, L., Zgajnar, J., Mora, J., Brewer, C., Gadea, N., Brady, A. F., Gallagher, D., Van Os, T., Donaldson, A., Stefansdottir, V., Barwell, J., James, P. A., Murphy, D., Friedman, E., Nicolai, N., Greenhalgh, L., Obeid, E., Murthy, V., Copakova, L., McGrath, J., Teo, S. H., Strom, S., Kast, K., Leongamornlert, D. A., Chamberlain, A., Pope, J., Newlin, A. C., Aaronson, N., Ardern-Jones, A., Bangma, C., Castro, E., Dearnaley, D., Eyfjord, J., Falconer, A., Foster, C. S., Gronberg, H., Hamdy, F. C., Johannsson, O., Khoo, V., Lubinski, J., Grindedal, E. M., McKinley, J., Shackleton, K., Mitra, A. V., Moynihan, C., Rennert, G., Suri, M., Tricker, K., Moss, S., Kote-Jarai, Z., Vickers, A., Lilja, H., Helfand, B. T., Eeles, R. A.. Prostate-specific antigen velocity in a prospective prostate cancer screening study of men with genetic predisposition. British Journal of Cancer. 2018. 118(2):266-276

128. Stockler, M. R.. Pooled RCTs: Reanalysis accounting for screening intensity suggests that screening reduces prostate cancer mortality. Annals of Internal Medicine. 2018. 168(2):JC5

129. Hung, W. W.. Low-intensity PSA-based screening did not reduce prostate cancer mortality. Journal of Clinical Outcomes Management. 2018. 25:#pages#

130. Fenner, A.. Prostate cancer: ERSPC calculator recalibrated for China. Nature Reviews Urology. 2017. 14(2):66

131. Armstrong, B. K., Barry, M. J., Frydenberg, M., Gardiner, R. A., Haines, I., Carter, S. M.. PSA testing for men at average risk of prostate cancer. Public health research & practice. 2017. 27:#pages#

132. Shoag, J. E., Schlegel, P. N., Hu, J. C.. Prostate-specific antigen screening: Time to change the dominant forces on the pendulum. Journal of Clinical Oncology. 2016. 34(29):3499-3501

133. Auvinen, A.. Prostate cancer: Prudent practice optimizes screening outcomes. Nature Reviews Urology. 2016. 13(7):376-377

134. Haines, I. E., Ablin, R. J., Gabor, G. L.. Screening for prostate cancer: Time to put all the data on the table. BMJ (Online). 2016. 353 (no pagination):#pages#

135. Weiss, N. S.. Enhancing the validity and generalizability of randomized trials of cancer screening. Epidemiologic Methods. 2015. 4(1):29-31

136. Olver, I.. Highlights of PSA testing guidelines. Cancer Forum. 2015. 39(3):161-163

137. Tormey, W. P.. Prostate Cancer Risk Calculators Using ERSPC-derived Data Underestimate the Risk if the WHO IRP 96/670 Standard Is Used in Prostate-specific Antigen Analysis. European Urology. 2015. 68(3):541-542

138. Bill-Axelson, A., Bratt, O.. Re: Screening and prostate cancer mortality: Results of the European Randomised study of screening for prostate cancer (ERSPC) at 13 years of follow-up. European Urology. 2015. 67(1):175

139. Arai, Y.. IJU this issue. International Journal of Urology. 2014. 21(4):351

140. Black, A.. Cohort study: A targeted approach reduces prostate cancer-specific (PSA) screening harms while preserving benefits. Evidence-Based Medicine. 2014. 19(5):186

141. Roobol, M. J., Bokhorst, L. P.. The ProtecT trial: What can we expect?. The Lancet Oncology. 2014. 15(10):1046-1047

142. Taneja, S. S.. Re: Strategy for detection of prostate cancer based on relation between prostate specific antigen at age 40-55 and long term risk of metastasis: Case-control study. Journal of Urology. 2014. 191(1):75

143. Wilt, T. J., Scardino, P. T., Carlsson, S. V., Basch, E.. Prostate-specific antigen screening in prostate cancer: Perspectives on the evidence. Journal of the National Cancer Institute. 2014. 106:#pages#

144. Albers, P., Arsov, C.. Re: Strategy for detection of prostate cancer based on relation between prostate specific antigen at age 40-55 and long term risk of metastasis: Case-control study. European Urology. 2013. 64(4):681-682

145. Taneja, S. S.. Re: An updated prostate cancer staging nomogram (Partin Tables) based on cases from 2006 to 2011. Journal of Urology. 2013. 189(4):1320

146. Taneja, S. S.. Re: Outcome following active surveillance of men with screen-detected prostate cancer. Results from the Goteborg randomised population-based prostate cancer screening trial. Journal of Urology. 2013. 189(1):124-125

147. Anonymous. Screening for prostate cancer: Recommendation statement. American Family Physician. 2013. 87(4):1-7

148. Penson, D. F., Barocas, D. A., Fleshner, N., Sanda, M. G., Greenfield, S.. Outcomes session. Urologic Oncology: Seminars and Original Investigations. 2012. 30(6):952-955

149. Basch, E., Oliver, T. K., Vickers, A., Thompson, I., Kantoff, P., Parnes, H., Loblaw, D. A., Roth, B., Williams, J., Nam, R. K.. Screening for Prostate Cancer with Prostate-Specific Antigen Testing: American Society of Clinical Oncology Provisional Clinical Opinion. Journal of Clinical Oncology. 2012. 30(24):3020-3025

150. Walsh, P. C.. Re: Prostate cancer screening in the randomized prostate, lung, colorectal, and ovarian cancer screening trial: Mortality results after 13 years of follow-up. Journal of Urology. 2012. 188(2):429-430

151. Vickers, A.. Re: Prostate cancer screening in the randomized prostate, lung, colorectal, and ovarian cancer screening trial: Mortality results after 13 years of follow-up. European Urology. 2012. 62(2):353

152. Dahm, P.. Review: Prostate cancer screening using PSA does not decrease mortality. Annals of Internal Medicine. 2011. 154(2):JC1-2

153. Farley, S. J.. Prostate cancer: Does PSA screening do more harm than good?. Nature Reviews Urology. 2009. 6(5):237

154. Marsden, T., McCartan, N., Hadley, J., Tuck, S., Brown, L., Haire, A. J., Moss, C. L., Green, S., Van Hemelrijck, M., Coolen, T., Santaolalla, A., Isaac, E., Brembilla, G., Kopcke, D., Giganti, F., Sidhu, H., Punwani, S., Emberton, M., Moore, C. M.. Update from the ReIMAGINE Prostate Cancer Screening Study NCT04063566: Inviting Men for Prostate Cancer Screening Using Magnetic Resonance Imaging. European Urology Focus. 2021. 7(3):503-505

155. Auvinen, A., Rannikko, A., Taari, K., Kujala, P., Mirtti, T., Kentt, auml;mies, A., Rinta-Kiikka, I., Lehtim, auml;ki, T., Oksala, N., Pettersson, K., Tammela, T. L.. A randomized trial of early detection of clinically significant prostate cancer (ProScreen): study design and rationale. European journal of epidemiology. 2017. Vol.32:521-527p

156. . Predicting Prostate Cancer in Elderly Men. #journal#. 2019. #volume#:#pages#

157. . Screening for Prostate Cancer in Older Patients (PLCO Screening Trial). #journal#. 2004. #volume#:#pages#

158. . Early vs. delayed screening for prostate cancer. #journal#. 2020. #volume#:#pages#

159. . Prostate Cancer Secondary Screening in Sapienza and Policlinico Umberto I. #journal#. 2021. #volume#:#pages#

160. . Prostate Cancer Detection Using the Stockholm3 Test and MR/Fusion Biopsies. #journal#. 2017. #volume#:#pages#

161. . A Randomized Trial of Early Detection of Clinically Significant Prostate Cancer (ProScreen). #journal#. 2018. #volume#:#pages#

162. . MRI Versus PSA in Prostate Cancer Screening. #journal#. 2016. #volume#:#pages#

163. Schr, ouml;der, F. H.. Screening for prostate cancer: preliminary evidence from randomized studies and epidemiological data. European journal of cancer. 1999. Vol.35:S300p

164. Hugosson, J., Aus, G., Bergdahl, S., Frosing, R., Lodding, P., Nyberg, M., Pileblad, E., Pihl, C. G.. Differences between prostate cancer cases found at prostate-specific antigen screening and a control group. Scandinavian journal of urology and nephrology. 1998. Supplementum. Vol.33:17p

165. Labrie, F., Dupont, A., Candas, B., Cusan, L., Gomez, J. L., Diamond, P., Belanger, A., Brousseau, G., Levesque, J.. Decrease of prostate cancer death by screening: first data from the Quebec prospective and randomized study. Proceedings of the american society of clinical oncology. 1998. 17:#pages#

166. Labrie, F., Candas, B., Cusan, L., Gomez, J. L., eacute;langer, A., Brousseau, G., eacute;vesque, E.. Decrease of prostate cancer death by screening: update of the Quebec prospective randomized controlled trial at 10 years of follow-up. Proceedings of the american society of clinical oncology. 1326. 19:#pages#

167. Kumar, V., Wang, H., Denofrio, D., Kent, D.. The clinical implications of a positive prostate cancer screen in patients undergoing a cardiac transplant evaluation. Journal of Clinical and Translational Science. 2018. #volume#:48

168. Institute for Quality Efficiency in Health Care. Prostate cancer screening with a PSA test: IQWiG Reports – Commission No. S19-01 [Internet]. Institute for Quality and Efficiency in Health Care. 2020. 06:10

169. Grossman, D. C., Curry, S. J., Owens, D. K., Bibbins-Domingo, K., Caughey, A. B., Davidson, K. W., Doubeni, C. A., Ebell, M., Epling, J. W., Kemper, A. R., Krist, A. H.,

Kubik, M., Seth Landefeld, C., Mangione, C. M., Silverstein, M., Simon, M. A., Siu, A. L., Tseng, C. W.. Screening for prostate cancer USPreventive servicestaskforcerecommendation statement. JAMA - Journal of the American Medical Association. 2018. 319(18):1901-1913

170. Bell, N., Connor Gorber, S., Shane, A., Joffres, M., Singh, H., Dickinson, J., Shaw, E., Dunfield, L., Tonelli, M.. Recommendations on screening for prostate cancer with the prostate-specific antigen test. Cmaj. 2014. 186(16):1225-1234

171. Huynh-Le, M. P., Fan, C. C., Karunamuni, R., Walsh, E. I., Turner, E. L., Lane, J. A., Martin, R. M., Neal, D. E., Donovan, J. L., Hamdy, F. C., Parsons, J. K., Eeles, R. A., Easton, D. F., Kote-Jarai, Z., Amin Al Olama, A., Benlloch Garcia, S., Muir, K., Gronberg, H., Wiklund, F., Aly, M., Schleutker, J., Sipeky, C., Tammela, T. L., Nordestgaard, B. G., Key, T. J., Travis, R. C., Pharoah, P. D. P., Pashayan, N., Khaw, K. T., Thibodeau, S. N., McDonnell, S. K., Schaid, D. J., Maier, C., Vogel, W., Luedeke, M., Herkommer, K., Kibel, A. S., Cybulski, C., Wokolorczyk, D., Kluzniak, W., Cannon-Albright, L. A., Brenner, H., Schottker, B., Holleczek, B., Park, J. Y., Sellers, T. A., Lin, H. Y., Slavov, C. K., Kaneva, R. P., Mitev, V. I., Batra, J., Clements, J. A., Spurdle, A. B., Teixeira, M. R., Paulo, P., Maia, S., Pandha, H., Michael, A., Mills, I. G., Andreassen, O. A., Dale, A. M., Seibert, T. M., Australian Prostate Cancer, BioResource, Consortium, Practical. A Genetic Risk Score to Personalize Prostate Cancer Screening, Applied to Population Data. Cancer Epidemiology, Biomarkers & Prevention. 2020. 29:1731-1738

172. Karlsson, A. A., Hao, S., Jauhiainen, A., Elfstrom, K. M., Egevad, L., Nordstrom, T., Heintz, E., Clements, M. S.. The cost-effectiveness of prostate cancer screening using the Stockholm3 test. PLoS ONE [Electronic Resource]. 2021. 16:e0246674

173. Jen, H. H., Chang, W. J., Hsu, C. Y., Yen, A. M., Auvinen, A., Chen, T. H., Chen, S. L.. Sojourn-time-corrected receiver operating characteristic curve (ROC) for prostate specific antigen (PSA) test in population-based prostate cancer screening. Scientific Reports. 2020. 10:20665

174. Booth, N., Rissanen, P., Tammela, T. L. J., Kujala, P., Stenman, U. H., Taari, K., Talala, K., Auvinen, A.. Cost-effectiveness analysis of PSA-based mass screening: Evidence from a randomised controlled trial combined with register data. PLoS ONE [Electronic Resource]. 2019. 14:e0224479

175. Gulati, R., Morgan, T. M., A'Mar, T., Psutka, S. P., Tosoian, J. J., Etzioni, R.. Overdiagnosis and Lives Saved by Reflex Testing Men with Intermediate Prostate-Specific Antigen Levels. Journal of the National Cancer Institute. 2020. 112(4):384-390

176. Breza, J., Subin, F., Bernadic, M., Tomas, M., Pindak, D.. The use of European Randomized study of Screening for Prostate Cancer calculator as a diagnostic tool for prostate biopsy indication. Bratislavske lekarske listy. 2019. 120(5):331-335

177. Tomer, A., Rizopoulos, D., Nieboer, D., Drost, F. J., Roobol, M. J., Steyerberg, E. W.. Personalized Decision Making for Biopsies in Prostate Cancer Active Surveillance Programs. Medical decision making : an international journal of the Society for Medical Decision Making. 2019. 39(5):499-508

178. Draisma, G., Boer, R., Otto, S. J., van der Cruijsen, I. W., Damhuis, R. A., Schr, ouml;der, F. H., de Koning, H. J.. Lead times and overdetection due to prostate-specific antigen screening: estimates from the European Randomized Study of Screening for Prostate Cancer. Journal of the National Cancer Institute. 2003. Vol.95:868-878p

179. Vickers, A., Cronin, A., Roobol, M., Savage, C., Peltola, M., Pettersson, K., Scardino, P. T., Schr, ouml;der, F., Lilja, H.. Reducing unnecessary biopsy during prostate cancer screening using a four-kallikrein panel: an independent replication. Journal of clinical oncology. 2010. Vol.28:2493-2498p

180. Gulati, R., Tsodikov, A., Wever, E. M., Mariotto, A. B., Heijnsdijk, E. A., Katcher, J., de Koning, H. J., Etzioni, R.. The impact of PLCO control arm contamination on perceived PSA screening efficacy. Cancer causes & control. 2012. Vol.23:827-835p

181. Heijnsdijk, E. A. M., Denham, D., De Koning, H. J.. The Cost-Effectiveness of Prostate Cancer Detection with the Use of Prostate Health Index. Value in health. 2016. 19:153-157

182. Heijnsdijk, E. A. M., Adolfsson, J., Auvinen, A., Roobol, M. J., Hugosson, J., de Koning, H. J.. The Impact of Design and Performance in Prostate-Specific Antigen Screening: Differences Between ERSPC Centers(Figure presented.). European Urology. 2019. Vol.76:276-279p

183. Loeb, S., Vonesh, E. F., Metter, E. J., Carter, H. B., Gann, P. H., Catalona, W. J.. What is the true number needed to screen and treat to save a life with prostate-specific antigen testing?. Journal of clinical oncology. 2011. Vol.29:464-467p

184. Yen, A. M., Auvinen, A., Schleutker, J., Wu, Y. Y., Fann, J. C., Tammela, T., Chen, S. L., Chiu, S. Y., Chen, H. H.. Prostate cancer screening using risk stratification based on a multi-state model of genetic variants. Prostate. 2015. Vol.75:825-835p

185. Shoaibi, A., Rao, G. A., Cai, B., Rawl, J., Haddock, K. S., Hebert, J. R.. Prostate Specific Antigen-Growth Curve Model to Predict High-Risk Prostate Cancer. Prostate. 2017. Vol.77:173-184p

186. Roobol, M. J., Verbeek, J. F. M., van der Kwast, T., Kummerlin, I. P., Kweldam, C. F., van Leenders, Gjlh. Improving the Rotterdam European Randomized Study of Screening for Prostate Cancer Risk Calculator for Initial Prostate Biopsy by Incorporating the 2014

International Society of Urological Pathology Gleason Grading and Cribriform growth. European urology. 2017. #volume#:#pages#

187. Hakama, M., Aro, J., Auvinen, A., Juusela, H., auml, auml;tt, auml;nen, L., Stenman, U. H., Tammela, T. L.. Randomized screening trial for prostate cancer in Finland. European urology. 2001. 39:#pages#

188. Draisma, G., De Koning, H. J.. MISCAN: estimating lead-time and over-detection by simulation. BJU international. 2003. 92:106-111

189. Boniol, M., Boyle, P., Autier, P., Ruffion, A., Perrin, P.. Critical role of prostate biopsy mortality in the number of years of life gained and lost within a prostate cancer screening programme. BJU international. 2012. Vol.110:1648-1652p

190. Wever, E. M., Hugosson, J., Heijnsdijk, E. A., Bangma, C. H., Draisma, G., de Koning, H. J.. To be screened or not to be screened? Modeling the consequences of PSA screening for the individual. British journal of cancer. 2012. Vol.107:778-784p

191. Nevalainen, J., Stenman, U. H., Tammela, T. L., Roobol, M., Carlsson, S., Talala, K., Schr, ouml;der, F. H., Auvinen, A.. What explains the differences between centres in the European screening trial? A simulation study. Cancer epidemiology. 2017. 46:14-19

192. Verbeek, J. F. M., Nieboer, D., Parker, C., Kattan, M. W., Steyerberg, E. W., Roobol, M. J.. A tool for shared decision making on referral for prostate biopsy in the primary care setting: integrating risks of cancer with life expectancy. Journal of personalized medicine. 2019. 9:#pages#

193. Keller, A., Gericke, C., Whitty, J. A., Yaxley, J., Kua, B., Coughlin, G., Gianduzzo, T.. A Cost-Utility Analysis of Prostate Cancer Screening in Australia. Applied Health Economics & Health Policy. 2017. 15:95-111

194. Gulati, R., Cheng, H. H., Lange, P. H., Nelson, P. S., Etzioni, R.. Screening Men at Increased Risk for Prostate Cancer Diagnosis: Model Estimates of Benefits and Harms. Cancer Epidemiology, Biomarkers & Prevention. 2017. 26:222-227

195. Xia, J., Gulati, R., Au, M., Gore, J. L., Lin, D. W., Etzioni, R.. Effects of screening on radical prostatectomy efficacy: the prostate cancer intervention versus observation trial. Journal of the National Cancer Institute. 2013. 105:546-50

196. Wang, L., Zhou, X. H.. Comparing the accuracy of screening tests with verification of disease status restricted to test positives. Statistics in Medicine. 2022. 41(6):994-1008

197. Perera, M., Smith, L., Thompson, I., Breemer, G., Papa, N., Patel, M. I., Swindle, P., Smith, E.. Advancing Traditional Prostate-specific Antigen Kinetics in the Detection of Prostate Cancer: A Machine Learning Model. European Urology Focus.. 2021. #volume#:#pages#

198. Ferraro, S., Bussetti, M., Bassani, N., Rossi, R. S., Incarbone, G. P., Bianchi, F., Maggioni, M., Runza, L., Ceriotti, F., Panteghini, M.. Definition of outcome-based prostate-specific antigen (Psa) thresholds for advanced prostate cancer risk prediction. Cancers. 2021. 13(14) (no pagination):#pages#

199. Liang, G., Zhang, P., Zhao, Z., He, Z., Luo, F., Chen, Z., Yang, Z., Zhang, Z., Xia, T., Liu, X., Zhang, Y., Ye, W.. China county-based prostate specific antigen screening for prostate cancer and a cost-effective analysis. Translational Andrology and Urology. 2021. 10(10):3787-3799

200. Bergengren, O., Westerberg, M., Holmberg, L., Stattin, P., Bill-Axelson, A., Garmo, H.. Variation in Prostate-Specific Antigen Testing Rates and Prostate Cancer Treatments and Outcomes in a National 20-Year Cohort. JAMA Network Open. 2021. (no pagination):#pages#

201. Heijnsdijk, E. A. M., Gulati, R., Tsodikov, A., Lange, J. M., Mariotto, A. B., Vickers, A. J., Carlsson, S. V., Etzioni, R.. Lifetime benefits and harms of prostate-specific antigen-based risk-stratified screening for prostate cancer. Journal of the National Cancer Institute. 2020. 112(10) (no pagination):#pages#

202. de Koning, H. J., Gulati, R., Moss, S. M., Hugosson, J., Pinsky, P. F., Berg, C. D., Auvinen, A., Andriole, G. L., Roobol, M. J., Crawford, E. D., Nelen, V., Kwiatkowski, M., Zappa, M., Lujan, M., Villers, A., de Carvalho, T. M., Feuer, E. J., Tsodikov, A., Mariotto, A. B., Heijnsdijk, E. A. M., Etzioni, R.. The efficacy of prostate-specific antigen screening: Impact of key components in the ERSPC and PLCO trials. Cancer. 2018. 124(6):1197-1206

203. Carlsson, S. V., de Carvalho, T. M., Roobol, M. J., Hugosson, J., Auvinen, A., Kwiatkowski, M., Villers, A., Zappa, M., Nelen, V., Paez, A., Eastham, J. A., Lilja, H., de Koning, H. J., Vickers, A. J., Heijnsdijk, E. A. M.. Estimating the harms and benefits of prostate cancer screening as used in common practice versus recommended good practice: A microsimulation screening analysis. Cancer. 2016. 122(21):3386-3393

204. Simpkin, A. J., Donovan, J. L., Tilling, K., Athene Lane, J., Martin, R. M., Albertsen, P. C., Bill-Axelson, A., Ballentine Carter, H., Bosch, J. L. H. R., Ferrucci, L., Hamdy, F. C., Holmberg, L., Jeffrey Metter, E., Neal, D. E., Parker, C. C., Metcalfe, C.. Prostate-specific antigen patterns in US and European populations: comparison of six diverse cohorts. BJU International. 2016. 118(6):911-918

205. Palma, A., Lounsbury, D. W., Schlecht, N. F., Agalliu, I.. A system dynamics model of serum prostate-specific antigen screening for prostate cancer. American Journal of Epidemiology. 2016. 183(3):227-236

206. Roobol, M. J., Vedder, M. M., Nieboer, D., Houlgatte, A., Vincendeau, S., Lazzeri, M., Guazzoni, G., Stephan, C., Semjonow, A., Haese, A., Graefen, M., Steyerberg, E. W.. Comparison of Two Prostate Cancer Risk Calculators that Include the Prostate Health Index. European Urology Focus. 2015. 1(2):185-190

207. Pashayan, N., Duffy, S. W., Neal, D. E., Hamdy, F. C., Donovan, J. L., Martin, R. M., Harrington, P., Benlloch, S., Amin Al Olama, A., Shah, M., Kote-Jarai, Z., Easton, D. F., Eeles, R., Pharoah, P. D.. Implications of polygenic risk-stratified screening for prostate cancer on overdiagnosis. Genetics in Medicine. 2015. 17(10):789-795

208. Nordstrom, T., Vickers, A., Assel, M., Lilja, H., Gronberg, H., Eklund, M.. Comparison between the four-kallikrein panel and prostate health index for predicting prostate cancer. European Urology. 2015. 68(1):139-146

209. De Carvalho, T. M., Heijnsdijk, E. A. M., De Koning, H. J.. Screening for prostate cancer in the US? Reduce the harms and keep the benefit. International Journal of Cancer. 2015. 136(7):1600-1607

210. Vertosick, E. A., Poon, B. Y., Vickers, A. J.. Relative value of race, family history and prostate specific antigen as indications for early initiation of prostate cancer screening. Journal of Urology. 2014. 192(3):724-729

211. Lansdorp-Vogelaar, I., Gulati, R., Mariotto, A. B., Schechter, C. B., De Carvalho, T. M., Knudsen, A. B., Van Ravesteyn, N. T., Heijnsdijk, E. A. M., Pabiniak, C., Van Ballegooijen, M., Rutter, C. M., Kuntz, K. M., Feuer, E. J., Etzioni, R., De Koning, H. J., Zauber, A. G., Mandelblatt, J. S.. Personalizing age of cancer screening cessation based on comorbid conditions: Model estimates of harms and benefits. Annals of Internal Medicine. 2014. 161(2):104-112

212. Zhao, R., Huang, Y., Cheng, G., Liu, J., Shao, P., Qin, C., Hua, L., Yin, C.. Developing a follow-Up strategy for patients with PSA ranging from 4 to 10 ng/ml via a new model to reduce unnecessary prostate biopsies. PLoS ONE. 2014. 9(9) (no pagination):#pages#

213. Gulati, R., Inoue, L. Y. T., Gore, J. L., Katcher, J., Etzioni, R.. Individualized estimates of overdiagnosis in screen-detected prostate cancer. Journal of the National Cancer Institute. 2014. 106:#pages#

214. Gulati, R., Tsodikov, A., Etzioni, R., Hunter-Merrill, R. A., Gore, J. L., Mariotto, A. B., Cooperberg, M. R.. Expected population impacts of discontinued prostate-specific antigen screening. Cancer. 2014. 120(22):3519-3526

215. Martin, A. J., Lord, S. J., Verry, H. E., Stockler, M. R., Emery, J. D.. Risk assessment to guide prostate cancer screening decisions: A cost-effectiveness analysis. Medical Journal of Australia. 2013. 198(10):546-550

216. Wu, G. H. M., Auvinen, A., Maattanen, L., Tammela, T. L. J., Stenman, U. H., Hakama, M., Yen, A. M. F., Chen, H. H.. Number of screens for overdetection as an indicator of absolute risk of overdiagnosis in prostate cancer screening. International Journal of Cancer. 2012. 131(6):1367-1375

217. Etzioni, R., Mucci, L., Chen, S., Johansson, J. E., Fall, K., Adami, H. O.. Increasing use of radical prostatectomy for nonlethal prostate cancer in Sweden. Clinical Cancer Research. 2012. 18(24):6742-6747

218. Wu, G. H. M., Auvinen, A., Yen, A. M. F., Hakama, M., Walter, S. D., Chen, H. H.. A stochastic model for survival of early prostate cancer with adjustments for leadtime, length bias, and over-detection. Biometrical Journal. 2012. 54(1):20-44

219. Nichol, M. B., Wu, J., Huang, J., DenHam, D., Frencher, S. K., Jacobsen, S. J.. Cost-effectiveness of prostate health index for prostate cancer detection. BJU International. 2012. 110(3):353-362

220. Heijnsdijk, E. A. M., Wever, E. M., Auvinen, A., Hugosson, J., Ciatto, S., Nelen, V., Kwiatkowski, M., Villers, A., Paez, A., Moss, S. M., Zappa, M., Tammela, T. L. J., Makinen, T., Carlsson, S., Korfage, I. J., Essink-Bot, M. L., Otto, S. J., Draisma, G., Bangma, C. H., Roobol, M. J., Schroder, F. H., De Koning, H. J.. Quality-of-life effects of prostate-specific antigen screening. New England Journal of Medicine. 2012. 367(7):595-605

221. Gordon, L. A. N., Kam, J., Gilbourd, D., Haxhimolla, H.. Australian ultrasound-guided biopsy trends: a 17-year analysis of national data. Prostate International. 2019. 7:150-155

222. Luo, Q., Yu, X. Q., Kahn, C., Egger, S., Patel, M. I., Grogan, P. B., Caruana, M., Canfell, K., Smith, D. P., O'Connell, D. L.. Changes in prostate cancer incidence, mortality and survival in relation to prostate specific antigen testing in New South Wales, Australia. Cancer Epidemiology. 2022. 78 (no pagination):#pages#

223. Ko, Y. H., Roh, K. C., Kim, B. H.. The national-wide incidence of prostate-specific antigen testing trend for a decade in Korea by age group. Investigative and Clinical Urology. 2022. 63(2):184-191

224. Borregales, L. D., DeMeo, G., Gu, X., Cheng, E., Dudley, V., Schaeffer, E. M., Nagar, H., Carlsson, S., Vickers, A., Hu, J. C.. Grade Migration of Prostate Cancer in the United

States during the last decade. Journal of the National Cancer Institute.. 2022. 28:#pages#

225. Lima, C. A., Da Silva, B. E. B., Hora, E. C., Lima, M. S., De Abreu Costa Brito, E., De Oliveira Santos, M., Da Silva, A. M., Nunes, M. A. P., De Farias Brito, H. L., Lima, M. M. M.. Trends in prostate cancer incidence and mortality to monitor control policies in a northeastern Brazilian state. PLoS ONE. 2021. 16(3 March) (no pagination):#pages#

226. Jeong, S. H., Raman, J. D.. Impact of the evolving United States Preventative Services Task Force policy statements on incidence and distribution of prostate cancer over 15 years in a statewide cancer registry. Prostate International. 2021. 9(1):12-17

227. Sheng, I. Y., Wei, W., Chen, Y. W., Gilligan, T. D., Barata, P. C., Ornstein, M. C., Rini, B. I., Garcia, J. A.. Implications of the United States Preventive Services Task Force Recommendations on Prostate Cancer Stage Migration. Clinical Genitourinary Cancer. 2021. 19(1):e12-e16

228. Saito, E., Hori, M., Matsuda, T., Yoneoka, D., Ito, Y., Katanoda, K.. Long-term trends in prostate cancer incidence by stage at diagnosis in Japan using the multiple imputation approach, 1993-2014. Cancer Epidemiology Biomarkers and Prevention. 2020. 29(6):1222-1228

229. Negoita, S., Feuer, E. J., Mariotto, A., Cronin, K. A., Petkov, V. I., Hussey, S. K., Benard, V., Henley, S. J., Anderson, R. N., Fedewa, S., Sherman, R. L., Kohler, B. A., Dearmon, B. J., Lake, A. J., Ma, J., Richardson, L. C., Jemal, A., Penberthy, L.. Annual Report to the Nation on the Status of Cancer, part II: Recent changes in prostate cancer trends and disease characteristics. Cancer. 2018. 124(13):2801-2814

230. Kvale, R., Myklebust, T. A., Engholm, G., Heinavaara, S., Wist, E., Moller, B.. Prostate and breast cancer in four Nordic countries: A comparison of incidence and mortality trends across countries and age groups 1975-2013. International Journal of Cancer. 2017. 141(11):2228-2242

231. Winter, A., Sirri, E., Jansen, L., Wawroschek, F., Kieschke, J., Castro, F. A., Krilaviciute, A., Holleczek, B., Emrich, K., Waldmann, A., Brenner, H., Geiss, K., Meyer, M., Eberle, A., Luttmann, S., Stabenow, R., Hentschel, S., Nennecke, A., Kajuter, H., Mattauch, V., Eisemann, N., Kraywinkel, K.. Comparison of prostate cancer survival in Germany and the USA: can differences be attributed to differences in stage distributions?. BJU International. 2017. 119(4):550-559

232. Shen, X., Kumar, P.. Trade-off between treatment of early prostate cancer and incidence of advanced prostate cancer in the prostate screening era. Journal of Urology. 2016. 195(5):1397-1402

233. Hu, J. C., Williams, S. B., Carter, S. C., Eggener, S. E., Prasad, S., Chamie, K., Trinh, Q. D., Sun, M., Nguyen, P. L., Lipsitz, S. R.. Population-based assessment of prostate-specific antigen testing for prostate cancer in the elderly. Urologic Oncology: Seminars and Original Investigations. 2015. 33(2):e29-69

234. Ranasinghe, W. K. B., Kim, S. P., Lawrentschuk, N., Sengupta, S., Hounsome, L., Barber, J., Jones, R., Davis, P., Bolton, D., Persad, R.. Population-based analysis of prostate-specific antigen (PSA) screening in younger men (<55 years) in Australia. BJU International. 2014. 113(1):77-83

235. Smailyte, G., Aleknaviciene, B.. Incidence of prostate cancer in Lithuania after introduction of the Early Prostate Cancer Detection Programme. Public Health. 2012. 126(12):1075-1077

236. Talala, Kirsi, Walter, Stephen, Taari, Kimmo, Tammela, Teuvo L. J., Kujala, Paula, Auvinen, Anssi (2024). Screening history and risk of death from prostate cancer: a nested case-control study within the screening arm of the Finnish Randomized Study of Screening for Prostate Cancer (FinRSPC) Cancer causes & control : CCC, 35(4), 695-703

237. Kensler, K. H., Mao, J., Davuluri, M. (2024). Frequency of Guideline-Discordant Prostate Cancer Screening among Older Males JAMA Network Open, 7(4), E248487

238. Tesfai, Abel, Norori, Natalia, Harding, Thomas A., Wong, Yui Hang, Hobbs, Matthew David (2024). Variation in harms and benefits of prostate-specific antigen screening for prostate cancer by socio-clinical risk factors: A rapid review BJUI compass, 5(5), 417-432

239. Tesfai, Abel, Norori, Natalia, Harding, Thomas A., Wong, Yui Hang, Hobbs, Matthew David (2024). The impact of pre-biopsy MRI and additional testing on prostate cancer screening outcomes: A rapid review BJUI compass, 5(4), 426-438

240. Matsukawa, Akihiro, Yanagisawa, Takafumi, Bekku, Kensuke, Kardoust Parizi, Mehdi, Laukhtina, Ekaterina, Klemm, Jakob, Chiujdea, Sever, Mori, Keiichiro, Kimura, Shoji, Fazekas, Tamas, Miszczyk, Marcin, Miki, Jun, Kimura, Takahiro, Karakiewicz, Pierre I., Rajwa, Pawel, Shariat, Shahrokh F. (2024). Comparing the Performance of Digital Rectal Examination and Prostate-specific Antigen as a Screening Test for Prostate Cancer: A Systematic Review and Meta-analysis European urology oncology, 7(4), 697-704

241. Feng, Xiaoshuang, Zahed, Hana, Onwuka, Justina, Callister, Matthew E. J., Johansson, Mattias, Etzioni, Ruth, Robbins, Hilary A. (2024). Cancer Stage Compared With Mortality as End Points in Randomized Clinical Trials of Cancer Screening: A Systematic Review and Meta-Analysis JAMA, 331(22), 1910-1917

242. Fazekas, Tamas, Shim, Sung Ryul, Basile, Giuseppe, Baboudjian, Michael, Koi, Tamas, Przydacz, Mikolaj, Abufaraj, Mohammad, Ploussard, Guillaume, Kasivisvanathan, Veeru, Rivas, Juan Gomez, Gandaglia, Giorgio, Szarvas, Tibor, Schoots, Ivo G., van den Bergh, Roderick C. N., Leapman, Michael S., Nyirady, Peter, Shariat, Shahrokh F.,

Rajwa, Pawel (2024). Magnetic Resonance Imaging in Prostate Cancer Screening: A Systematic Review and Meta-Analysis JAMA oncology, 10(6), 745-754

243. Garraway, Isla P., Carlsson, Sigrid V., Nyame, Yaw A., Vassy, Jason L., Chilov, Marina, Fleming, Mark, Frencher, Stanley K., George, Daniel J., Kibel, Adam S., King, Sherita A., Kittles, Rick, Mahal, Brandon A., Pettaway, Curtis A., Rebbeck, Timothy, Rose, Brent, Vince, Randy, Winn, Robert A., Yamoah, Kosj, Oh, William K. (2024). Prostate Cancer Foundation Screening Guidelines for Black Men in the United States NEJM evidence, 3(5), EVIDoa2300289

244. Kensler, Kevin H., Johnson, Roman, Morley, Faith, Albrair, Mohamed, Dickerman, Barbra A., Gulati, Roman, Holt, Sarah K., Iyer, Hari S., Kibel, Adam S., Lee, Jenney R., Preston, Mark A., Vassy, Jason L., Wolff, Erika M., Nyame, Yaw A., Etzioni, Ruth, Rebbeck, Timothy R. (2024). Prostate cancer screening in African American men: a review of the evidence Journal of the National Cancer Institute, 116(1), 34-52

245. Bretthauer, Michael, Wieszczy, Paulina, Loberg, Magnus, Kaminski, Michal F., Werner, Tarjei Fiskergard, Helsingen, Lise M., Mori, Yuichi, Holme, Oyvind, Adami, Hans-Olov, Kalager, Mette (2023). Estimated Lifetime Gained With Cancer Screening Tests: A Meta-Analysis of Randomized Clinical Trials JAMA internal medicine, 183(11), 1196-1203

246. Lin, K., Lipsitz, R., Miller, T., Janakiraman, S., Force, U. S. Preventive Services Task (2008). Benefits and harms of prostate-specific antigen screening for prostate cancer: an evidence update for the U.S. Preventive Services Task Force Annals of internal medicine, 149(3), 192

247. Dave, Priya, Carlsson, Sigrid V., Watts, Kara (2024). Randomized trials of PSA screening Urologic oncology, #volume#(#issue#), #Pages#

248. Schroder, F. H. (1994). Screening for prostate cancer Lancet (london, england), 343(8910), 1438

249. Park, Hyerin, Chen, David C., Mazzone, Elio, Grummet, Jeremy, Perera, Marlon L., Murphy, Declan G. (2024). Re: Biomarker vs MRI-Enhanced Strategies for Prostate Cancer Screening. The STHLM3-MRI Randomized Clinical Trial European urology, #volume#(#issue#), #Pages#

250. Golijanin, Borivoj, Mega, Anthony, Golijanin, Dragan (2024). Re: Ivo I. de Vos II, Sebastiaan Remmers, Renee Hogenhout, Monique J. Roobol, ERSPC Rotterdam Study Group. Prostate Cancer Mortality Among Elderly Men After Discontinuing Organised Screening: Long-term Results from the European Randomized Study of Screening for Prostate Cancer Rotterdam. Eur Urol 2024;85:74-81 European urology, 85(5), e143-e144

251. Nguyen, Dong, Ho, Linh, Nguyen, Sang (2023). Understanding the study of 21-year follow-up results of the Rotterdam section of the European Randomized Study of Screening for Prostate Cancer World journal of urology, 42(1), 3

252. Bratt, Ola, Auvinen, Anssi (2023). First diagnostic results from Gothenburg-2 screening trial Scandinavian journal of urology, 58(#issue#), 2-3

253. Stone, A., Goldberg, H. (2024). Modifying and personalizing prostate cancer screening Translational Andrology and Urology, 13(5), 899-901

254. Hamdy, F. C. (2022). Prostate-Specific Antigen Testing for Prostate Cancer Screening - Is the Message Getting Through? JAMA Oncology, 8(1), 47-49

255. Hoffman, R. M. (2023). After PSA screening, MRI-targeted vs. systematic biopsy detected fewer clinically insignificant prostate cancers Annals of Internal Medicine, 176(4), JC44

256. Dasarathy, J., Rajesh, R. (2020). PSA cancer screening: A case for shared decision-making Journal of Family Practice, 69(1), 26-46

257. Scherer, T. P., Poyet, C. (2024). Prognostic properties of the baseline prostate-specific antigen value-insights from the European randomized study of screening for prostate cancer Translational andrology and urology, 13(3), 473

258. Schroder, F. H., Roobol, M. J. (2010). The REDUCE Trial European urology, 58(2), 253

259. Walsh, P. C. (2002). Large-scale randomized prostate cancer screening trials: program performance in the European Randomized Screening for Prostate Cancer trial and the Prostate, Lung, Colorectal and Ovary Cancer Trial Journal of urology, 168(4 Pt 1), 1639

260. Vellekoop, A., Loeb, S. (2013). Update of randomized trials for prostate cancer screening Reviews in urology, 15(1), 37

261. Elwood, M. (2004). A misleading paper on prostate cancer screening Prostate, 61(4), 372

262. Ilic, D., Dahm, P. (2015). Although the evidence is not clear, decreases in prostate cancer mortality in specific subgroups of men may be due to screening Evidence-based medicine, 20(3), 102

263. (2016). Correction: absolute Effect of Prostate Cancer Screening: balance of Benefits and Harms by Center within the European Randomized Study of Prostate Cancer Screening Clinical cancer research, 22(14), 3702

264. Barratt, A. L., Coates, A. S. (2004). Screening decreases prostate cancer death: first analysis of the 1988 Quebec Prospective Randomized Controlled Trial Medical journal of Australia, 181(4), 213

265. Harwood, R. H. (1997). Screening for prostate cancer. Randomised trials of treatment in early disease are important BMJ (Clinical research ed.), 315(7101), 186

266. Richard, F. (2009). Screening for prostate cancer: results from the first screening round in the French population of the ERSPC trial Dépistage du cancer de la prostate : les données du premier tour de dépistage de la population française participant à l'essai européen ERSPC, 19(7), 499

267. Farley, S. J. (2010). Prostate cancer: pSA-based screening in Sweden dramatically reduces disease-specific mortality Nature reviews urology, 7(8), 415

268. Walsh, P. C. (2011). Interval cancers in the antwerp European randomised study of screening for prostate cancer study, using a 6 year screening interval Journal of urology, 185(5), 1707

269. (2003). Annual PSA screening is unnecesary in some men South African medical journal, 93(7), 492

270. Parker, C., Dearnaley, D. (2002). Re: all-cause mortality in randomized trials of cancer screening Journal of the National Cancer Institute, 94(11), 861

271. Kirby, R. S., Eeles, R. A., Kote-Jarai, Z., Guy, M., Easton, D., Fitzpatrick, J. M. (2010). Screening for prostate cancer: the way ahead BJU international, 105(3), 295

272. Mian, B. M. (2010). Prostate cancer screening and mortality: comparison of recent randomized controlled clinical trials Urologic oncology, 28(3), 233

273. Ilic, D., Green, S. (2009). Prostate specific antigen for detecting early prostate cancer BMJ (Clinical research ed.), 339(#issue#), b3572

274. Schroder, F. H., Roobol, M. J. (2010). ERSPC and PLCO Prostate Cancer Screening Studies: what Are the Differences? European urology, 58(1), 46

275. Catalona, W. J. (1997). Screening for prostate cancer. Early screening is important despite lack of data from trials BMJ (Clinical research ed.), 315(7101), 187

276. Loeb, S., Partin, A. W. (2009). Randomized trials of prostate cancer screening Reviews in urology, 11(3), 179

277. Cusan, L. (1994). Prostate cancer screening with PSA, DRE and TRUS The Canadian journal of oncology, 4 Suppl 1(#issue#), 63

278. Prorok, P. (1994). The National Cancer Institute Multi-Screening Trial The Canadian journal of oncology, 4 Suppl 1(#issue#), 98

279. Gohagan, J. K., Kramer, B. S., Greenwald, P. (1994). "Screening for prostate cancer" American journal of preventive medicine, 10(4), 245

280. Gulati, Roman, Jiao, Boshen, Al-Faouri, Ra'ad, Sharma, Vidit, Kaul, Sumedh, Fleishman, Aaron, Wymer, Kevin, Boorjian, Stephen A., Olumi, Aria F., Etzioni, Ruth, Gershman, Boris (2024). Lifetime Health and Economic Outcomes of Biparametric Magnetic Resonance Imaging as First-Line Screening for Prostate Cancer : A Decision Model Analysis Annals of internal medicine, 177(7), 871-881

281. Remmers, Sebastiaan, Nieboer, Daan, Roobol, Monique J. (2023). The Patient Journey from Randomization to Detection of Prostate Cancer and Death: Results from ERSPC Rotterdam European urology open science, 51(#issue#), 1-6

282. Carlsson, Sigrid V., Arnsrud Godtman, Rebecka, Pihl, Carl-Gustav, Vickers, Andrew, Lilja, Hans, Hugosson, Jonas, Mansson, Marianne (2023). Young Age on Starting Prostate-specific Antigen Testing Is Associated with a Greater Reduction in Prostate Cancer Mortality: 24-Year Follow-up of the Goteborg Randomized Population-based Prostate Cancer Screening Trial European urology, 83(2), 103-109

283. Palsdottir, T., Soderback, H., Jaderling, F., Bergman, M., Vigneswaran, H., Gronberg, H. (2024). The Capio Prostate Cancer Center Model for Prostate Cancer Diagnostics-Real-world Evidence from 2018 to 2022 European Urology Open Science, 61(#issue#), 29-36

284. Stone, B. V., Labban, M., Beatrici, E., Filipas, D. K., D'Amico, A. V., Lipsitz, S. R., Choueiri, T. K., Kibel, A. S., Cole, A. P., Iyer, H. S., Trinh, Q. D. (2024). The Association of County-level Prostate-specific Antigen Screening with Metastatic Prostate Cancer and Prostate Cancer Mortality European Urology Oncology, 7(3), 563-569

285. (2005). European Randomized Study of Screening for Prostate Cancer #journal#, #volume#(#issue#), #Pages#

286. (2022). A prospective, randomized controlled clinical trial of prostate biopsy guided by prostate puncture positioning system #journal#, #volume#(#issue#), #Pages#

287. Schroder, F. H. (1994). The European Screening Study for Prostate Cancer The Canadian journal of oncology, 4 Suppl 1(#issue#), 102

288. BPAC. *Testing for Prostate Cancer: Helping Patients to Decide*.; 2020. www.bpac.org.nz/2020/prostate.aspx

289. BPAC. *Testing for Prostate Cancer in Primary Care*.; 2020. www.bpac.org.nz/report/psa.aspx

290. Franco GS, Hardie R, Li L, et al. Prostate-specific antigen testing of asymptomatic men in Australia: an observational study based on electronic general practice data. *Medical Journal of Australia*. 2021;215(5):228-229. doi:10.5694/mja2.51147

291. Spencer, K. *Screening for Prostate Cancer*. Prostate Cancer Foundation of Australia Accessed May 27, 2025. pcfa.org.au

RCT published prior to 2019 (n=154)

1. Yang, T. K., Chuang, P. C., Yen, A. M., Chen, H. H., Chen, S. L.. Gene-Prostate-Specific-Antigen-Guided Personalized Screening for Prostate Cancer. Genes. 2019. 10:24

2. Beemsterboer, P. M., Kranse, R., de Koning, H. J., Habbema, J. D., Schr, ouml;der, F. H.. Changing role of 3 screening modalities in the European randomized study of

screening for prostate cancer (Rotterdam). International journal of cancer. 1999. Vol.84:437-441p

3. Schr, ouml;der, F. H., van der Cruijsen-Koeter, I., de Koning, H. J., Vis, A. N., Hoedemaeker, R. F., Kranse, R.. Prostate cancer detection at low prostate specific antigen. Journal of urology. 2000. Vol.163:806-812p

4. Candas, B., Cusan, L., Gomez, J. L., Diamond, P., Suburu, R. E., eacute;vesque, J., Brousseau, G., eacute;langer, A., Labrie, F.. Evaluation of prostatic specific antigen and digital rectal examination as screening tests for prostate cancer. Prostate. 2000. Vol.45:19-35p

5. Vis, A. N., Hoedemaeker, R. F., Roobol, M., van der Kwast, T. H., Schr, ouml;der, F. H.. Tumor characteristics in screening for prostate cancer with and without rectal examination as an initial screening test at low PSA (0.0-3.9 ng/ml). Prostate. 2001. Vol.47:252-261p

6. auml;kinen, T., Tammela, T. L., Stenman, U. H., auml, auml;tt, auml;nen, L., Rannikko, S., Aro, J., Juusela, H., Hakama, M., Auvinen, A.. Family history and prostate cancer screening with prostate-specific antigen. Journal of clinical oncology. 2002. Vol.20:2658-2663p

7. Labrie, F., Candas, B., Cusan, L., Gomez, J. L., eacute;langer, A., Brousseau, G., Chevrette, E., eacute;vesque, J.. Screening decreases prostate cancer mortality: 11-year follow-up of the 1988 Quebec prospective randomized controlled trial. Prostate. 2004. Vol.59:311-318p

8. Finne, P., Finne, R., Bangma, C., Hugosson, J., Hakama, M., Auvinen, A., Stenman, U. H.. Algorithms based on prostate-specific antigen (PSA), free PSA, digital rectal examination and prostate volume reduce false-positive PSA results in prostate cancer screening. International journal of cancer. 2004. Vol.111:310-315p

9. Andriole, G. L., Levin, D. L., Crawford, E. D., Gelmann, E. P., Pinsky, P. F., Chia, D., Kramer, B. S., Reding, D., Church, T. R., Grubb, R. L., Izmirlian, G., Ragard, L. R., Clapp, J. D., Prorok, P. C., Gohagan, J. K., Team, Plco Project. Prostate Cancer Screening in the Prostate, Lung, Colorectal and Ovarian (PLCO) Cancer Screening Trial: findings from the initial screening round of a randomized trial. Journal of the National Cancer Institute. 2005. Vol.97:433-438p

10. Ankerst, D. P., Thompson, I. M.. Sensitivity and specificity of prostate-specific antigen for prostate cancer detection with high rates of biopsy verification. Archivio italiano di urologia, andrologia : organo ufficiale [di] societa italiana di ecografia urologica e nefrologica. 2006. Vol.78:125-129p

11. Gosselaar, C., Roobol, M. J., Roemeling, S., Wolters, T., van Leenders, G. J., Schr, ouml;der, F. H.. The value of an additional hypoechoic lesion-directed biopsy core for detecting prostate cancer. BJU international. 2008. Vol.101:685-690p

12. Rosario, D. J., Lane, J. A., Metcalfe, C., Catto, J. W., Dedman, D., Donovan, J. L., Neal, D. E., Hamdy, F. C., Protec, T. Study Group. Contribution of a single repeat PSA test to prostate cancer risk assessment: experience from the ProtecT study. European urology. 2008. Vol.53:777-784p

13. Gosselaar, C., Roobol, M. J., Roemeling, S., Schr, ouml;der, F. H.. The role of the digital rectal examination in subsequent screening visits in the European randomized study of screening for prostate cancer (ERSPC), Rotterdam. European urology. 2008. Vol.54:581-588p

14. Schr, ouml;der, F. H., Hugosson, J., Roobol, M. J., Tammela, T. L., Ciatto, S., Nelen, V., Kwiatkowski, M., Lujan, M., Lilja, H., Zappa, M., Denis, L. J., Recker, F., Berenguer, A., auml, auml;tt, auml;nen, L., Bangma, C. H., Aus, G., Villers, A., Rebillard, X., van der Kwast, T., Blijenberg, B. G., Moss, S. M., de Koning, H. J., Auvinen, A., Investigators, Erspc. Screening and prostate-cancer mortality in a randomized European study. New England journal of medicine. 2009. Vol.360:1320-1328p

15. Andriole, G. L., Crawford, E. D., Grubb, R. L., Buys, S. S., Chia, D., Church, T. R., Fouad, M. N., Gelmann, E. P., Kvale, P. A., Reding, D. J., Weissfeld, J. L., Yokochi, L. A., O'Brien, B., Clapp, J. D., Rathmell, J. M., Riley, T. L., Hayes, R. B., Kramer, B. S., Izmirlian, G., Miller, A. B., Pinsky, P. F., Prorok, P. C., Gohagan, J. K., Berg, C. D., Team, Plco Project. Mortality results from a randomized prostate-cancer screening trial. New England journal of medicine. 2009. Vol.360:1310-1319p

16. Moore, A. L., Dimitropoulou, P., Lane, A., Powell, P. H., Greenberg, D. C., Brown, C. H., Donovan, J. L., Hamdy, F. C., Martin, R. M., Neal, D. E.. Population-based prostate-specific antigen testing in the UK leads to a stage migration of prostate cancer. BJU international. 2009. Vol.104:1592-1598p

17. Finne, P., Fallah, M., Hakama, M., Ciatto, S., Hugosson, J., de Koning, H., Moss, S., Nelen, V., Auvinen, A.. Lead-time in the European Randomised Study of Screening for Prostate Cancer. European journal of cancer (Oxford, England :. 2010. Vol.46:3102-3108p

18. Lane, J. A., Hamdy, F. C., Martin, R. M., Turner, E. L., Neal, D. E., Donovan, J. L.. Latest results from the UK trials evaluating prostate cancer screening and treatment: the CAP and ProtecT studies. European journal of cancer (Oxford, England :. 2010. Vol.46:3095-3101p

19. Crawford, E. D., Grubb, R., Black, A., Andriole, G. L., Chen, M. H., Izmirlian, G., Berg, C. D., D'Amico, A. V.. Comorbidity and mortality results from a randomized prostate cancer screening trial. Journal of clinical oncology. 2011. Vol.29:355-361p

20. Rietbergen, J. B., Kranse, R., Kirkels, W. J., De Koning, H. J., Schr, ouml;der, F. H.. Evaluation of prostate-specific antigen, digital rectal examination and transrectal ultrasonography in population-based screening for prostate cancer: improving the efficiency of early detection. British journal of urology. 1997. 79:57-63

21. Kilpel, auml;inen, T. P., Tammela, T. L., Roobol, M., Hugosson, J., Ciatto, S., Nelen, V., Moss, S., auml, auml;tt, auml;nen, L., Auvinen, A.. False-positive screening results in the European randomized study of screening for prostate cancer. European journal of cancer (Oxford, England :. 2011. Vol.47:2698-2705p

22. Gosselaar, C., Roobol, M. J., van den Bergh, R. C., Wolters, T., Schr, ouml;der, F. H.. Digital rectal examination and the diagnosis of prostate cancer--a study based on 8 years and three screenings within the European Randomized Study of Screening for Prostate Cancer (ERSPC), Rotterdam. European urology. 2009. Vol.55:139-146p

23. Andriole, G. L., Crawford, E. D., Grubb, R. L., Buys, S. S., Chia, D., Church, T. R., Fouad, M. N., Isaacs, C., Kvale, P. A., Reding, D. J., Weissfeld, J. L., Yokochi, L. A., O'Brien, B., Ragard, L. R., Clapp, J. D., Rathmell, J. M., Riley, T. L., Hsing, A. W., Izmirlian, G., Pinsky, P. F., Kramer, B. S., Miller, A. B., Gohagan, J. K., Prorok, P. C., Team, Plco Project. Prostate cancer screening in the randomized Prostate, Lung, Colorectal, and Ovarian Cancer Screening Trial: mortality results after 13 years of follow-up. Journal of the National Cancer Institute. 2012. Vol.104:125-132p

24. Roobol, M. J., Kranse, R., Bangma, C. H., van Leenders, A. G., Blijenberg, B. G., van Schaik, R. H., Kirkels, W. J., Otto, S. J., van der Kwast, T. H., de Koning, H. J., Schr, ouml;der, F. H., Group, Erspc Rotterdam Study. Screening for prostate cancer: results of the Rotterdam section of the European randomized study of screening for prostate cancer. European urology. 2013. Vol.64:530-539p

25. Bokhorst, L. P., Zhu, X., Bul, M., Bangma, C. H., Schr, ouml;der, F. H., Roobol, M. J.. Positive predictive value of prostate biopsy indicated by prostate-specific-antigen-based prostate cancer screening: trends over time in a European randomized trial*. BJU international. 2012. Vol.110:1654-1660p

26. Carlsson, S., Maschino, A., Schr, ouml;der, F., Bangma, C., Steyerberg, E. W., van der Kwast, T., van Leenders, G., Vickers, A., Lilja, H., Roobol, M. J.. Predictive value of four kallikrein markers for pathologically insignificant compared with aggressive prostate cancer in radical prostatectomy specimens: results from the European Randomized Study of Screening for Prostate Cancer section Rotterdam. European urology. 2013. Vol.64:693-699p

27. Wei, J. T., Feng, Z., Partin, A. W., Brown, E., Thompson, I., Sokoll, L., Chan, D. W., Lotan, Y., Kibel, A. S., Busby, J. E., Bidair, M., Lin, D. W., Taneja, S. S., Viterbo, R., Joon, A. Y., Dahlgren, J., Kagan, J., Srivastava, S., Sanda, M. G.. Can urinary PCA3 supplement PSA in the early detection of prostate cancer?. Journal of clinical oncology. 2014. Vol.32:4066-4072p

28. Saarim, auml;ki, L., Tammela, T. L., auml, auml;tt, auml;nen, L., Taari, K., Kujala, P. M., Raitanen, J., Auvinen, A.. Family history in the Finnish Prostate Cancer Screening Trial. International journal of cancer. 2015. Vol.136:2172-2177p

29. Randazzo, M., uuml;ller, A., Carlsson, S., Eberli, D., Huber, A., Grobholz, R., Manka, L., Mortezavi, A., Sulser, T., Recker, F., Kwiatkowski, M.. A positive family history as a risk factor for prostate cancer in a population-based study with organised prostate-specific antigen screening: results of the Swiss European Randomised Study of Screening for Prostate Cancer (ERSPC, Aarau). BJU international. 2016. Vol.117:576-583p

30. Bokhorst, L. P., Venderbos, L. D. F., Schroder, F. H., Bangma, C. H., Steyerberg, E. W., Roobol, M. J.. Do Treatment Differences between Arms Affect the Main Outcome of ERSPC Rotterdam?. Journal of urology. 2015. 194:336-342

31. Pinsky, P. F., Prorok, P. C., Yu, K., Kramer, B. S., Black, A., Gohagan, J. K., Crawford, E. D., Grubb, R. L., Andriole, G. L.. Extended mortality results for prostate cancer screening in the PLCO trial with median follow-up of 15 years. Cancer. 2017. Vol.123:592-599p

32. Tsodikov, A., Gulati, R., Heijnsdijk, E. A. M., Pinsky, P. F., Moss, S. M., Qiu, S., De Carvalho, T. M., Hugosson, J., Berg, C. D., Auvinen, A., Andriole, G. L., Roobol, M. J., Crawford, E. D., Nelen, V., Kwiatkowski, M., Zappa, M., Lujan, M., Villers, A., Feuer, E. J., De Koning, H. J., Mariotto, A. B., Etzioni, R.. Reconciling the effects of screening on prostate cancer mortality in the ERSPC and PLCO trials. Annals of internal medicine. 2017. Vol.167:449-455p

33. Norming, U., Gustafsson, O., Nyman, C. R., Almgard, L. E., Fredriksson, A., Gustafsson, G., et al.. Digital rectal examination versus transrectal ultrasound in detection of prostate cancer. Preliminary results from a study of a randomly selected population. Acta oncologica (Stockholm, Sweden). 1991. Vol.30:277-279p

34. Rubio-Briones, J., Casanova, J., Martinez, F., Dominguez-Escrig, J. L., Fernandez-Serra, A., Dumont, R., Ramirez-Backhaus, M., Gomez-Ferrer, A., Collado, A., Rubio, L., Molina, A., Vanaclocha, M., Sala, D., Lopez-Guerrero, J. A.. PCA3 como biomarcador de segunda linea en un programa de screening oportunista prospectivo, aleatorizado y controlado, PCA3 as a second-line biomarker in a prospective controlled randomized opportunistic prostate cancer screening programme. Actas urologicas espanolas. 2017. Vol.41:300-308p

35. Franlund, M., Arnsrud Godtman, R., Carlsson, S. V., Lilja, H., Mansson, M., Stranne, J., Hugosson, J.. Prostate cancer risk assessment in men with an initial P.S.A. below 3 ng/mL: results from the Göteborg randomized population-based prostate cancer screening trial. Scandinavian Journal of Urology. 2018. Vol.52:256-262p

36. Essink Bot, M. L., de Koning, H. J., Nijs, H. G., Kirkels, W. J., van der Maas, P. J., Schroder, F. H.. Short-term effects of population-based screening for prostate cancer on health-related quality of life. J natl cancer inst. 1998. 90:925-931

37. Murtola, T. J., Virkku, A., Talala, K., Stenman, U. H., Taari, K., Tammela, T. L. J., Auvinen, A.. Outcomes of Prostate Cancer Screening by 5&agr;-Reductase Inhibitor Use. Journal of urology. 2017. Vol.198:305-309p

38. Miller, E. A., Pinsky, P. F., Black, A., Andriole, G. L., Pierre-Victor, D.. Secondary prostate cancer screening outcomes by race in the Prostate, Lung, Colorectal, and Ovarian (PLCO) Screening Trial. Prostate. 2018. Vol.78:830-838p

39. Isola, J., Auvinen, A., Poutiainen, M., Kakkola, L., auml;rvinen, T. A., auml, auml;tt, auml;nen, L., Stenman, U. H., Tammela, T., Hakama, M., Visakorpi, T.. Predictors of biological aggressiveness of prostate specific antigen screening detected prostate cancer. Journal of urology. 2001. Vol.165:1569-1574p

40. Beemsterboer, P. M., de Koning, H. J., Kranse, R., Trienekens, P. H., van der Maas, P. J., Schr, ouml;der, F. H.. Prostate specific antigen testing and digital rectal examination before and during a randomized trial of screening for prostate cancer: european randomized study of screening for prostate cancer, Rotterdam. Journal of urology. 2000. Vol.164:1216-1220p

41. Rietbergen, J. B. W., Hoedemaeker, R. F., Boeken Kruger, A. E., Kirkels, W. J., Schroder, F. H.. The changing pattern of prostate cancer at the time of diagnosis: characteristics of screen detected prostate cancer in a population based screening study. Journal of urology. 1999. Vol.161:1192-1198p

42. Hugosson, J.. Prostate specific antigen based biennial screening is sufficient to detect almost all prostate cancers while still curable. Journal of urology. 2003. Vol.169:1720-1723p

43. Makinen, T., Tammela, T. L. J., Hakama, M., Stenman, U. H., Rannikko, S., Aro, J., Juusela, H., Maattanen, L., Auvinen, A.. Tumor characteristics in a population-based prostate cancer screening trial with prostate-specific antigen. Clinical cancer research. 2003. Vol.9:2435-2439p

44. van der Cruijsen-Koeter, I. W., Vis, A. N., Roobol, M. J., Wildhagen, M. F., de Koning, H. J., van der Kwast, T. H., Schr, ouml;der, F. H.. Comparison of screen detected and

clinically diagnosed prostate cancer in the European randomized study of screening for prostate cancer, section rotterdam. Journal of urology. 2005. Vol.174:121-125p

45. Kwiatkowski, M., Huber, A., Moschopulos, M., Lehmann, K., Wernli, M., Hafeli, A., Recker, F.. Screening for prostate cancer: results of a prospective trial in Canton Aargau, Switzerland. Swiss medical weekly. 2004. Vol.134:580-585p

46. auml;kinen, T., Tammela, T. L., Stenman, U. H., auml, auml;tt, auml;nen, L., Aro, J., Juusela, H., Martikainen, P., Hakama, M., Auvinen, A.. Second round results of the Finnish population-based prostate cancer screening trial. Clinical cancer research. 2004. Vol.10:2231-2236p

47. Roemeling, S., Roobol, M. J., de Vries, S. H., Gosselaar, C., van der Kwast, T. H., Schr, ouml;der, F. H.. Prevalence, treatment modalities and prognosis of familial prostate cancer in a screened population. Journal of urology. 2006. Vol.175:1332-1336p

48. Gosselaar, C., Roobol, M. J., Roemeling, S., de Vries, S. H., Cruijsen-Koeter, Iv, van der Kwast, T. H., Schr, ouml;der, F. H., European Randomized Study of Screening for Prostate, Cancer. Screening for prostate cancer without digital rectal examination and transrectal ultrasound: results after four years in the European Randomized Study of Screening for Prostate Cancer (ERSPC), Rotterdam. Prostate. 2006. Vol.66:625-631p

49. Roemeling, S., Roobol, M. J., Gosselaar, C., Schr, ouml;der, F. H.. Biochemical progression rates in the screen arm compared to the control arm of the Rotterdam Section of the European Randomized Study of Screening for Prostate Cancer (ERSPC). Prostate. 2006. Vol.66:1076-1081p

50. Roobol, M. J., Zappa, M., auml, auml;tt, auml;nen, L., Ciatto, S.. The value of different screening tests in predicting prostate biopsy outcome in screening for prostate cancer data from a multicenter study (ERSPC). Prostate. 2007. Vol.67:439-446p

51. Thompson, I. M., Tangen, C. M., Goodman, P. J., Lucia, M. S., Parnes, H. L., Lippman, S. M., Coltman, C. A.. Finasteride improves the sensitivity of digital rectal examination for prostate cancer detection. Journal of urology. 2007. Vol.177:1749-1752p

52. Gosselaar, C., Kranse, R., Roobol, M. J., Roemeling, S., Schr, ouml;der, F. H.. The interobserver variability of digital rectal examination in a large randomized trial for the screening of prostate cancer. Prostate. 2008. Vol.68:985-993p

53. Chybowski, F. M., Bergstralh, E. J., Oesterling, J. E.. The effect of digital rectal examination on the serum prostate specific antigen concentration: results of a randomized study. Journal of urology. 1992. Vol.148:83-86p

54. Jegu, J., Tretarre, B., Grosclaude, P., Rebillard, X., Bataille, V., Malavaud, B., Iborra, F., Salama, G., Rischmann, P., Villers, A.. Results and participation factors to the European Randomized study of Screening for Prostate Cancer (ERSPC) with Prostate Specific Antigen: french departments of Tarn and Herault. Progres en urologie. 2009. Vol.19:487-498p

55. Hugosson, J., Carlsson, S., Aus, G., Bergdahl, S., Khatami, A., Lodding, P., Pihl, C. G., Stranne, J., Holmberg, E., Lilja, H.. Mortality results from the Goteborg randomised population-based prostate-cancer screening trial. Lancet oncology. 2010. Vol.11:725-732p

56. Kilpelainen, T. P., Auvinen, A., Maattanen, L., Kujala, P., Ruutu, M., Stenman, U. H., Tammela, T. L. J.. Results of the three rounds of the Finnish prostate cancer screening trial - The incidence of advanced cancer is decreased by screening. International journal of cancer. 2010. Vol.127:1699-1705p

57. Booth, N., Rissanen, P., Tammela, T. L. J., Maattanen, L., Taari, K., Auvinen, A.. Health-related quality of life in the finnish trial of screening for prostate cancer. European urology. 2014. Vol.65:39-47p

58. Luj, aacute;n, M., aacute;ez, Aacute, Berenguer, A., Rodr, iacute;guez, J. A.. Mortality due to prostate cancer in the Spanish arm of the European Randomized Study of Screening for Prostate Cancer (ERSPC). Results after a 15-year follow-up. Actas urologicas espanolas. 2012. Vol.36:403-409p

59. van Leeuwen, P. J., van den Bergh, R. C., Wolters, T., Zhu, X., Bul, M., Schr, ouml;der, F. H., Bangma, C. H., Roobol, M. J.. Critical assessment of prebiopsy parameters for predicting prostate cancer metastasis and mortality. Canadian journal of urology. 2011. Vol.18:6018-6024p

60. Sandblom, G., Varenhorst, E., Rosell, J., Lofman, O., Carlsson, P.. Random is ed prostate cancer screening trial: 20 Year follow-up. Bmj. 2011. 342:#pages#

61. Schroder, F. H., Hugosson, J., Roobol, M. J., Tammela, T. L. J., Ciatto, S., Nelen, V., Kwiatkowski, M., Lujan, M., Lilja, H., Zappa, M., Denis, L. J., Recker, F., Paez, A., Maattanen, L., Bangma, C. H., Aus, G., Carlsson, S., Villers, A., Rebillard, X., Van Der Kwast, T., Kujala, P. M., Blijenberg, B. G., Stenman, U. H., Huber, A., Taari, K., Hakama, M., Moss, S. M., De Koning, H. J., Auvinen, A.. Prostate-cancer mortality at 11 years of follow-up. New England journal of medicine. 2012. Vol.366:981-990p

62. Kilpelainen, T. P., Tammela, T. L. J., Malila, N., Hakama, M., Santti, H., Maattanen, L., Stenman, U. H., Kujala, P., Auvinen, A.. The Finnish prostate cancer screening trial: analyses on the screening failures. International journal of cancer. 2015. Vol.136:2437-2443p

63. Arnsrud Godtman, R., Holmberg, E., Lilja, H., Stranne, J., Hugosson, J.. Opportunistic Testing Versus Organized Prostate-specific Antigen Screening: outcome After 18 Years in the Goteborg Randomized Population-based Prostate Cancer Screening Trial. European urology. 2015. Vol.68:354-360p

64. Labrie, F., Candas, B., Dupont, A., Cusan, L., Gomez, J. L., Suburu, R. E., Diamond, P., eacute;vesque, J., Belanger, A.. Screening decreases prostate cancer death: first analysis of the 1988 Quebec prospective randomized controlled trial. Prostate. 1999. Vol.38:83-91p

65. Auvinen, A., Moss, S. M., Tammela, T. L. J., Taari, K., Roobol, M. J., Schroder, F. H., Bangma, C. H., Carlsson, S., Aus, G., Zappa, M., Puliti, D., Denis, L. J., Nelen, V., Kwiatkowski, M., Randazzo, M., Paez, A., Lujan, M., Hugosson, J.. Absolute effect of prostate cancer screening: balance of benefits and harms by center within the European Randomized Study of prostate cancer screening. Clinical cancer research. 2016. Vol.22:243-249p

66. Godtman, R. A., Carlsson, S., Holmberg, E., Stranne, J., Hugosson, J.. The Effect of Start and Stop Age at Screening on the Risk of Being Diagnosed with Prostate Cancer. Journal of urology. 2016. Vol.195:1390-1396p

67. Shoag, J., Mittal, S., Halpern, J. A., Scherr, D., Hu, J. C., Barbieri, C. E.. Lethal Prostate Cancer in the PLCO Cancer Screening Trial. European urology. 2016. Vol.70:2-5p

68. Halpern, J. A., Shoag, J. E., Mittal, S., Oromendia, C., Ballman, K. V., Hershman, D. L., Wright, J. D., Shih, Y. Ct, Nguyen, P. L., Hu, J. C.. Prognostic Significance of Digital Rectal Examination and Prostate Specific Antigen in the Prostate, Lung, Colorectal and Ovarian (PLCO) Cancer Screening Arm. Journal of urology. 2017. Vol.197:363-368p

69. Kilpelainen, T. P., Talala, K., Raitanen, J., Taari, K., Kujala, P., Tammela, T. L. J., Auvinen, A.. Prostate cancer and socioeconomic status in the finnish randomized study of screening for prostate cancer. American Journal of Epidemiology. 2016. Vol.184:720-731p

70. Murtola, T. J., Vettenranta, A. M., Talala, K., Taari, K., Stenman, U. H., Tammela, T. L. J., Auvinen, A.. Outcomes of Prostate-specific Antigen-based Prostate Cancer Screening Among Men Using Nonsteroidal Anti-inflammatory Drugs. European Urology Focus. 2018. Vol.4:851-857p

71. Labrie, F., Dupont, A., Suburu, R., Cusan, L., Gomez, J. L., Koutsilieris, M., Diamond, P., Emond, J., Lemay, M., Candas, B.. Optimized strategy for detection of early stage, curable prostate cancer: role of prescreening with prostate-specific antigen. Clinical and investigative medicine. 1993. Medecine clinique et experimentale. Vol.16:425-439p

72. Shr, ouml;der, F. H., Damhuis, R. A., Kirkels, W. J., De Koning, H. J., Kranse, R., Nus, H. G., Blijenberg, B. G.. European randomized study of screening for prostate cancer--the Rotterdam pilot studies. International journal of cancer. 1996. Vol.65:145-151p

73. Labrie, F., Candas, B., Cusan, L., Gomez, J. L., Diamond, P., Suburu, R., Lemay, M.. Diagnosis of advanced or noncurable prostate cancer can be practically eliminated by prostate-specific antigen. Urology. 1996. Vol.47:212-217p

74. Auvinen, A., Tammela, T., Stenman, U. H., Uusi, Erkkil, auml, I., Leinonen, J., Schr, ouml;der, F. H., Hakama, M.. Screening for prostate cancer using serum prostate-specific antigen: a randomised, population-based pilot study in Finland. British journal of cancer. 1996. Vol.74:568-572p

75. Schr, ouml;der, F. H., Bangma, C. H.. The European Randomized Study of Screening for Prostate Cancer (ERSPC). British journal of urology. 1997. 79:68-71

76. Schr, ouml;der, F. H., van der Maas, P., Beemsterboer, P., Kruger, A. B., Hoedemaeker, R., Rietbergen, J., Kranse, R.. Evaluation of the digital rectal examination as a screening test for prostate cancer. Rotterdam section of the European Randomized Study of Screening for Prostate Cancer. Journal of the National Cancer Institute. 1998. Vol.90:1817-1823p

77. auml, auml;tt, auml;nen, L., Auvinen, A., Stenman, U. H., Rannikko, S., Tammela, T., Aro, J., Juusela, H., Hakama, M.. European randomized study of prostate cancer screening: first-year results of the Finnish trial. British journal of cancer. 1999. Vol.79:1210-1214p

78. Schr, ouml;der, F. H., Roobol-Bouts, M., Vis, A. N., van der Kwast, T., Kranse, R.. Prostate-specific antigen-based early detection of prostate cancer--validation of screening without rectal examination. Urology. 2001. Vol.57:83-90p

79. Hoedemaeker, R. F., van der Kwast, T. H., Boer, R., de Koning, H. J., Roobol, M., Vis, A. N., Schr, ouml;der, F. H.. Pathologic features of prostate cancer found at population-based screening with a four-year interval. Journal of the National Cancer Institute. 2001. Vol.93:1153-1158p

80. de Koning, H. J., Auvinen, A., Berenguer Sanchez, A., Calais da Silva, F., Ciatto, S., Denis, L., Gohagan, J. K., Hakama, M., Hugosson, J., Kranse, R., Nelen, V., Prorok, P. C., Schr, ouml;der, F. H., European Randomized Screening for Prostate Cancer, Trial, International Prostate Cancer Screening Trials Evaluation, Group. Large-scale randomized prostate cancer screening trials: program performances in the European Randomized Screening for Prostate Cancer trial and the Prostate, Lung, Colorectal and Ovary cancer trial. International journal of cancer. 2002. Vol.97:237-244p

81. Vis, A. N., Kranse, R., Roobol, M., van der Kwast, T. H., Schr, ouml;der, F. H.. Serendipity in detecting disease in low prostate-specific antigen ranges. BJU international. 2002. Vol.89:384-389p

82. Finne, P., Auvinen, A., Aro, J., Juusela, H., auml, auml;tt, auml;nen, L., Rannikko, S., Hakama, M., Tammela, T. L., Stenman, U. H.. Estimation of prostate cancer risk on the basis of total and free prostate-specific antigen, prostate volume and digital rectal examination. European urology. 2002. Vol.41:619-26; discussion 626-7p

83. Roobol, M. J., Kirkels, W. J., Schr, ouml;der, F. H.. Features and preliminary results of the Dutch centre of the ERSPC (Rotterdam, the Netherlands). BJU international. 2003. 92:48-54

84. Kwiatkowski, M., Huber, A., Stamm, B., Lehmann, K., Wernli, M., auml;feli, A., Recker, F.. Features and preliminary results of prostate cancer screening in Canton Aargau, Switzerland. BJU international. 2003. 92:44-47

85. Hugosson, J., Aus, G., Bergdahl, S., Fernlund, P., Fr, ouml;sing, R., Lodding, P., Pihl, C. G., Lilja, H.. Population-based screening for prostate cancer by measuring free and total serum prostate-specific antigen in Sweden. BJU international. 2003. 92:39-43

86. Hugosson, J., Aus, G., Lilja, H., Lodding, P., Pihl, C. G.. Results of a randomized, population-based study of biennial screening using serum prostate-specific antigen measurement to detect prostate carcinoma. Cancer. 2004. Vol.100:1397-1405p

87. Postma, R., Roobol, M., Schr, ouml;der, F. H., van der Kwast, T. H.. Potentially advanced malignancies detected by screening for prostate carcinoma after an interval of 4 years. Cancer. 2004. Vol.100:968-975p

88. Auvinen, A., auml, auml;tt, auml;nen, L., Finne, P., Stenman, U. H., Aro, J., Juusela, H., Rannikko, S., Tammela, T. L., Hakama, M.. Test sensitivity of prostate-specific antigen in the Finnish randomised prostate cancer screening trial. International journal of cancer. 2004. Vol.111:940-943p

89. Otto, S. J., Schr, ouml;der, F. H., de Koning, H. J.. Low all-cause mortality in the volunteer-based Rotterdam section of the European randomised study of screening for prostate cancer: self-selection bias?. Journal of medical screening. 2004. Vol.11:89-92p

90. Sandblom, G., Varenhorst, E., ouml;fman, O., Rosell, J., Carlsson, P.. Clinical consequences of screening for prostate cancer: 15 years follow-up of a randomised controlled trial in Sweden. European urology. 2004. Vol.46:717-23; discussion 724p

91. Aus, G., Damber, J. E., Khatami, A., Lilja, H., Stranne, J., Hugosson, J.. Individualized screening interval for prostate cancer based on prostate-specific antigen level: results of a prospective, randomized, population-based study. Archives of internal medicine. 2005. Vol.165:1857-1861p

92. Pinsky, P. F., Kramer, B. S., Crawford, E. D., Grubb, R. L., Urban, D. A., Andriole, G. L., Chia, D., Levin, D. L., Gohagan, J. K.. Prostate volume and prostate-specific antigen levels in men enrolled in a large screening trial. Urology. 2006. Vol.68:352-356p

93. van der Cruijsen-Koeter, I. W., Roobol, M. J., Wildhagen, M. F., van der Kwast, T. H., Kirkels, W. J., Schr, ouml;der, F. H.. Tumor characteristics and prognostic factors in two subsequent screening rounds with four-year interval within prostate cancer screening trial, ERSPC Rotterdam. Urology. 2006. Vol.68:615-620p

94. Postma, R., van Leenders, A. G., Roobol, M. J., Schr, ouml;der, F. H., van der Kwast, T. H.. Tumour features in the control and screening arm of a randomized trial of prostate cancer. European urology. 2006. Vol.50:70-75p

95. auml, auml;tt, auml;nen, L., Hakama, M., Tammela, T. L., Ruutu, M., Ala-Opas, M., Juusela, H., Martikainen, P., Stenman, U. H., Auvinen, A.. Specificity of serum prostate-specific antigen determination in the Finnish prostate cancer screening trial. British journal of cancer. 2007. Vol.96:56-60p

96. Aus, G., Bergdahl, S., Lodding, P., Lilja, H., Hugosson, J.. Prostate cancer screening decreases the absolute risk of being diagnosed with advanced prostate cancer--results from a prospective, population-based randomized controlled trial. European urology. 2007. Vol.51:659-664p

97. Roobol, M. J., Grenabo, A., Schr, ouml;der, F. H., Hugosson, J.. Interval cancers in prostate cancer screening: comparing 2- and 4-year screening intervals in the European Randomized Study of Screening for Prostate Cancer, Gothenburg and Rotterdam. Journal of the National Cancer Institute. 2007. Vol.99:1296-1303p

98. Raaijmakers, R., de Vries, S. H., Blijenberg, B. G., Wildhagen, M. F., Postma, R., Bangma, C. H., Darte, C., Schr, ouml;der, F. H.. hK2 and free PSA, a prognostic combination in predicting minimal prostate cancer in screen-detected men within the PSA range 4-10 ng/ml. European urology. 2007. Vol.52:1358-1364p

99. Wilkinson, S., Warren, K., Ramsden, A., Matthews, A., Chodak, G.. Do "rapid" PSA assays reduce anxiety and stress of prostate cancer patients undergoing regular review? A prospective evaluation. Urology. 2008. Vol.71:567-572p

100. Romero, F. R., Romero, A. W., Brenny Filho, T., Bark, N. M., Yamazaki, D. S., de Oliveira, F. C.. Patients' perceptions of pain and discomfort during digital rectal exam for prostate cancer screening. Archivos espanoles de urologia. 2008. Vol.61:850-854p

101. Laurila, M., Tammela, T. L., Auvinen, A., Isola, J., Visakorpi, T., Luukkaala, T., auml, auml;tt, auml;nen, L., Ruutu, M., Ala-Opas, M., Mildh, M., Martikainen, P.. Biological aggressiveness of prostate cancer in the Finnish screening trial. International journal of cancer. 2009. Vol.124:547-552p

102. Grubb, R. L., Pinsky, P. F., Greenlee, R. T., Izmirlian, G., Miller, A. B., Hickey, T. P., Riley, T. L., Mabie, J. E., Levin, D. L., Chia, D., Kramer, B. S., Reding, D. J., Church, T. R., Yokochi, L. A., Kvale, P. A., Weissfeld, J. L., Urban, D. A., Buys, S. S., Gelmann, E. P., Ragard, L. R., Crawford, E. D., Prorok, P. C., Gohagan, J. K., Berg, C. D., Andriole, G. L.. Prostate cancer screening in the Prostate, Lung, Colorectal and Ovarian cancer screening trial: update on findings from the initial four rounds of screening in a randomized trial. BJU international. 2008. Vol.102:1524-1530p

103. Rietbergen, J. B., Kranse, R., Hoedemaeker, R. F., Kruger, A. E., Bangma, C. H., Kirkels, W. J., Schr, ouml;der, F. H.. Comparison of prostate-specific antigen corrected for total prostate volume and transition zone volume in a population-based screening study. Urology. 1998. Vol.52:237-246p

104. Croswell, J. M., Kramer, B. S., Kreimer, A. R., Prorok, P. C., Xu, J. L., Baker, S. G., Fagerstrom, R., Riley, T. L., Clapp, J. D., Berg, C. D., Gohagan, J. K., Andriole, G. L., Chia, D., Church, T. R., Crawford, E. D., Fouad, M. N., Gelmann, E. P., Lamerato, L., Reding, D. J., Schoen, R. E.. Cumulative incidence of false-positive results in repeated, multimodal cancer screening. Annals of family medicine. 2009. Vol.7:212-222p

105. Carlsson, S., Aus, G., Wessman, C., Hugosson, J.. Anxiety associated with prostate cancer screening with special reference to men with a positive screening test (elevated PSA) - Results from a prospective, population-based, randomised study. European journal of cancer (Oxford, England :. 2007. Vol.43:2109-2116p

106. Bergdahl, A. G., Aus, G., Lilja, H., Hugosson, J.. Risk of dying from prostate cancer in men randomized to screening: differences between attendees and nonattendees. Cancer. 2009. Vol.115:5672-5679p

107. Kilpel, auml;inen, T. P., Tammela, T. L., auml, auml;tt, auml;nen, L., Kujala, P., Stenman, U. H., Ala-Opas, M., Murtola, T. J., Auvinen, A.. False-positive screening results in the Finnish prostate cancer screening trial. British journal of cancer. 2010. Vol.102:469-474p

108. Kerkhof, M., Roobol, M. J., Cuzick, J., Sasieni, P., Roemeling, S., Schr, ouml;der, F. H., Steyerberg, E. W.. Effect of the correction for noncompliance and contamination on the

estimated reduction of metastatic prostate cancer within a randomized screening trial (ERSPC section Rotterdam). International journal of cancer. 2010. Vol.127:2639-2644p

109. van Leeuwen, P. J., Kranse, R., Hakulinen, T., Roobol, M. J., de Koning, H. J., Bangma, C. H., Schr, ouml;der, F. H.. Disease-specific mortality may underestimate the total effect of prostate cancer screening. Journal of medical screening. 2010. Vol.17:204-210p

110. Nelen, V., Thys, G., Hermans, A., D'Hooge, K., Dourcy-Belle-Rose, B., Coebergh, J. W., Roobol, M., Denis, L.. Interval cancers in the Antwerp European randomised study of screening for prostate cancer study, using a 6 year screening interval. European journal of cancer (Oxford, England :. 2010. Vol.46:3090-3094p

111. Boevee, S. J., Venderbos, L. D., Tammela, T. L., Nelen, V., Ciatto, S., Kwiatkowski, M., aacute;ez, A., Malavaud, B., Hugosson, J., Roobol, M. J.. Change of tumour characteristics and treatment over time in both arms of the European Randomized study of Screening for Prostate Cancer. European journal of cancer (Oxford, England :. 2010. Vol.46:3082-3089p

112. Carlsson, S., Aus, G., Bergdahl, S., Khatami, A., Lodding, P., Stranne, J., Hugosson, J.. The excess burden of side-effects from treatment in men allocated to screening for prostate cancer. The Göteborg randomised population-based prostate cancer screening trial. European journal of cancer (Oxford, England :. 2011. Vol.47:545-553p

113. Zhu, X., van Leeuwen, P. J., Bul, M., Otto, S. J., de Koning, H. J., Bangma, C. H., Schr, ouml;der, F. H., Roobol, M. J.. Disease-specific survival of men with prostate cancer detected during the screening interval: results of the European randomized study of screening for prostate cancer-Rotterdam after 11 years of follow-up. European urology. 2011. Vol.60:330-336p

114. Linder, S. K., Swank, P. R., Vernon, S. W., Morgan, R. O., Mullen, P. D., Volk, R. J.. Is a prostate cancer screening anxiety measure invariant across two different samples of age-appropriate men?. BMC medical informatics and decision making. 2012. 12:#pages#

115. Pinsky, P. F., Black, A., Parnes, H. L., Grubb, R., David Crawford, E., Miller, A., Reding, D., Andriole, G.. Prostate cancer specific survival in the Prostate, Lung, Colorectal, and Ovarian (PLCO) Cancer Screening Trial. Cancer epidemiology. 2012. Vol.36:e401-6p

116. Vasarainen, H., Malmi, H., auml, auml;tt, auml;nen, L., Ruutu, M., Tammela, T., Taari, K., Rannikko, A., Auvinen, A.. Effects of prostate cancer screening on health-related quality of life: results of the Finnish arm of the European randomized screening trial (ERSPC). Acta oncologica (Stockholm, Sweden). 2013. Vol.52:1615-1621p

117. Kilpel, auml;inen, T. P., Tammela, T. L., Malila, N., Hakama, M., Santti, H., auml, auml;tt, auml;nen, L., Stenman, U. H., Kujala, P., Auvinen, A.. Prostate cancer mortality in the Finnish randomized screening trial. Journal of the National Cancer Institute. 2013. Vol.105:719-725p

118. van Leeuwen, P. J., Roobol, M. J., Kranse, R., Zappa, M., Carlsson, S., Bul, M., Zhu, X., Bangma, C. H., Schr, ouml;der, F. H., Hugosson, J.. Towards an optimal interval for prostate cancer screening. European urology. 2012. Vol.61:171-176p

119. Arsov, C., Becker, N., Hadaschik, B. A., Hohenfellner, M., Herkommer, K., Gschwend, J. E., Imkamp, F., Kuczyk, M. A., Antoch, G., Kristiansen, G., Siener, R., Semjonow, A., Hamdy, F. C., Lilja, H., Vickers, A. J., Schr, ouml;der, F. H., Albers, P.. Prospective randomized evaluation of risk-adapted prostate-specific antigen screening in young men: the PROBASE trial. European urology. 2013. Vol.64:873-875p

120. Grenabo Bergdahl, A., Holmberg, E., Moss, S., Hugosson, J.. Incidence of prostate cancer after termination of screening in a population-based randomised screening trial. European urology. 2013. Vol.64:703-709p

121. Rubio-Briones, J., Casanova, J., Dumont, R., Rubio, L., Fernandez-Serra, A., Casanova-Salas, I., Dom, iacute;nguez-Escrig, J., Ram, iacute;rez-Backhaus, M., Collado, A., oacute;mez-Ferrer, A., Iborra, I., Monr, oacute;s, J. L., Ric, oacute;s, J. V., Solsona, E., Salas, D., Mart, iacute;nez, F., Lopez-Guerrero, J. A.. Optimizing prostate cancer screening; prospective randomized controlled study of the role of PSA and PCA3 testing in a sequential manner in an opportunistic screening program. Actas urologicas espanolas. 2014. Vol.38:217-223p

122. Bokhorst, L. P., Bangma, C. H., van Leenders, G. J., Lous, J. J., Moss, S. M., Schr, ouml;der, F. H., Roobol, M. J.. Prostate-specific antigen-based prostate cancer screening: reduction of prostate cancer mortality after correction for nonattendance and contamination in the Rotterdam section of the European Randomized Study of Screening for Prostate Cancer. European urology. 2014. Vol.65:329-336p

123. Kranse, R., van Leeuwen, P. J., Hakulinen, T., Hugosson, J., Tammela, T. L., Ciatto, S., Roobol, M. J., Zappa, M., Aus, G., Bangma, C. H., Moss, S. M., Auvinen, A., Schr, ouml;der, F. H.. Excess all-cause mortality in the evaluation of a screening trial to account for selective participation. Journal of medical screening. 2013. Vol.20:39-45p

124. Buzzoni, C., Auvinen, A., Roobol, M. J., Carlsson, S., Moss, S. M., Puliti, D., de Koning, H. J., Bangma, C. H., Denis, L. J., Kwiatkowski, M., Lujan, M., Nelen, V., Paez, A., Randazzo, M., Rebillard, X., Tammela, T. L., Villers, A., Hugosson, J., Schr, ouml;der, F. H., Zappa, M.. Metastatic Prostate Cancer Incidence and Prostate-specific Antigen Testing: new Insights from the European Randomized Study of Screening for Prostate Cancer. European urology. 2015. Vol.68:885-890p

125. Kilpel, auml;inen, T. P., auml;kinen, T., Karhunen, P. J., Aro, J., Lahtela, J., Taari, K., Talala, K., Tammela, T. L., Auvinen, A.. Estimating bias in causes of death ascertainment in the Finnish Randomized Study of Screening for Prostate Cancer. Cancer epidemiology. 2016. 45:1-5

126. Bokhorst, L. P., Kranse, R., Venderbos, L. D., Salman, J. W., van Leenders, G. J., Schr, ouml;der, F. H., Bangma, C. H., Roobol, M. J., Group, Erspc Rotterdam Study. Differences in Treatment and Outcome After Treatment with Curative Intent in the Screening and Control Arms of the ERSPC Rotterdam. European urology. 2015. Vol.68:179-182p

127. Luj, aacute;n, M., aacute;ez, Aacute, Angulo, J. C., Andr, eacute;s, G., Gimbernat, H., Redondo, C., Torres, G. M., Berenguer, A.. Update of the results of the Spanish branch of the European Randomized Study on Screening for Prostate Cancer (ERSPC). Actas urologicas espanolas. 2015. Vol.39:405-413p

128. Hakama, M., Moss, S. M., Stenman, U. H., Roobol, M. J., Zappa, M., Carlsson, S., Randazzo, M., Nelen, V., Hugosson, J.. Design-corrected variation by centre in mortality reduction in the ERSPC randomised prostate cancer screening trial. Journal of medical screening. 2017. Vol.24:98-103p

129. Grenabo Bergdahl, A., Wilder, auml;ng, U., Aus, G., Carlsson, S., Damber, J. E., Fr, aring;nlund, M., Geterud, K., Khatami, A., Socratous, A., Stranne, J., Hellstr, ouml;m, M., Hugosson, J.. Role of Magnetic Resonance Imaging in Prostate Cancer Screening: a Pilot Study Within the Göteborg Randomised Screening Trial. European urology. 2016. Vol.70:566-573p

130. van Leeuwen, P. J., Kranse, R., Hakulinen, T., Hugosson, J., Tammela, T. L., Ciatto, S., Roobol, M. J., Zappa, M., de Koning, H. J., Bangma, C. H., Moss, S. M., Auvinen, A., Schr, ouml;der, F. H.. Impacts of a population-based prostate cancer screening programme on excess total mortality rates in men with prostate cancer: a randomized controlled trial. Journal of medical screening. 2013. Vol.20:33-38p

131. Rubio-Briones, J., Casanova, J., Mart, iacute;nez, F., Dom, iacute;nguez-Escrig, J. L., Fern, aacute;ndez-Serra, A., Dumont, R., Ram, iacute;rez-Backhaus, M., oacute;mez-Ferrer, A., Collado, A., Rubio, L., Molina, A., Vanaclocha, M., Sala, D., Lopez-Guerrero, J. A.. PCA3 as a second-line biomarker in a prospective controlled randomized opportunistic prostate cancer screening programme. Actas urologicas espanolas. 2017. Vol.41:300-308p

132. Hugosson, J., Godtman, R. A., Carlsson, S. V., Aus, G., Grenabo Bergdahl, A., Lodding, P., Pihl, C. G., Stranne, J., Holmberg, E., Lilja, H.. Eighteen-year follow-up of the Göteborg Randomized Population-based Prostate Cancer Screening Trial: effect of sociodemographic variables on participation, prostate cancer incidence and mortality. Scandinavian journal of urology. 2018. Vol.52:27-37p

133. Prorok, P. C., Wright, P., Riley, T. R., Kramer, B. S., Berg, C. D., Gohagan, J. K.. Overall and Multiphasic Findings of the Prostate, Lung, Colorectal and Ovarian (PLCO) Randomized Cancer Screening Trial. Reviews on recent clinical trials. 2018. Vol.13:257-273p

134. Varenhorst, E., Carlsson, P., Capik, E., ouml;fman, O., Pedersen, K. V.. Repeated screening for carcinoma of the prostate by digital rectal examination in a randomly selected population. Acta oncologica (Stockholm, Sweden). 1992. Vol.31:815-821p

135. Saarimaki, L., Hugosson, J., Tammela, T. L., Carlsson, S., Talala, K., Auvinen, A.. Impact of Prostatic-specific Antigen Threshold and Screening Interval in Prostate Cancer Screening Outcomes: comparing the Swedish and Finnish European Randomised Study of Screening for Prostate Cancer Centres. European urology focus. 2019. Vol.5:186-191p

136. Lewicki, P., Shoag, J., Golombos, D. M., Oromendia, C., Ballman, K. V., Halpern, J. A., Stone, B. V., O'Malley, P., Barbieri, C. E., Scherr, D. S.. Prognostic Significance of a Negative Prostate Biopsy: An Analysis of Subjects Enrolled in a Prostate Cancer Screening Trial. Journal of Urology. 2017. 197:1014-1019

137. Grenabo Bergdahl, A., Wilderang, U., Aus, G., Carlsson, S., Damber, J. E., Franlund, M., Geterud, K., Khatami, A., Socratous, A., Stranne, J., Hellstrom, M., Hugosson, J.. Role of Magnetic Resonance Imaging in Prostate Cancer Screening: A Pilot Study Within the Goteborg Randomised Screening Trial. European Urology. 2016. 70:566-573

138. Schroder, F. H., Hugosson, J., Roobol, M. J., Tammela, T. L., Zappa, M., Nelen, V., Kwiatkowski, M., Lujan, M., Maattanen, L., Lilja, H., Denis, L. J., Recker, F., Paez, A., Bangma, C. H., Carlsson, S., Puliti, D., Villers, A., Rebillard, X., Hakama, M., Stenman, U. H., Kujala, P., Taari, K., Aus, G., Huber, A., van der Kwast, T. H., van Schaik, R. H., de Koning, H. J., Moss, S. M., Auvinen, A., Investigators, Erspc. Screening and prostate cancer mortality: results of the European Randomised Study of Screening for Prostate Cancer (ERSPC) at 13 years of follow-up. Lancet. 2014. 384:2027-35

139. Cui, T., Kovell, R. C., Terlecki, R. P.. Is it time to abandon the digital rectal examination? Lessons from the PLCO Cancer Screening Trial and peer-reviewed literature. Current Medical Research & Opinion. 2016. 32:1663-1669

140. Gilbert, R., Tilling, K., Martin, R. M., Lane, J. A., Davis, M., Hamdy, F. C., Neal, D. E., Donovan, J. L., Metcalfe, C.. Developing new age-specific prostate-specific antigen thresholds for testing for prostate cancer. Cancer Causes and Control. 2018. 29(3):383-388

141. Miller, E. A., Pinsky, P. F., Pierre-Victor, D.. The relationship between diabetes, prostate-specific antigen screening tests, and prostate cancer. Cancer Causes and Control. 2018. 29(10):907-914

142. Pakarainen, T., Raitanen, J., Talala, K., Taari, K., Kujala, P., Tammela, T. L., Auvinen, A.. Number of Screening Rounds and Postscreening Prostate Cancer Incidence: Results from the Finnish Section of the European Randomized Study of Screening for Prostate Cancer Study. European Urology. 2016. 70(3):499-505

143. Lujan, M., Paez, A., Angulo, J. C., Granados, R., Nevado, M., Torres, G. M., Berenguer, A.. Long-term prostate-specific antigen contamination in the Spanish arm of the European Randomized Study of Screening for Prostate Cancer (ERSPC). Actas urologicas espanolas. 2016. 40(3):164-172

144. Liss, M. A., Chen, H., Hemal, S., Krane, S., Kane, C. J., Xu, J., Kader, A. K.. Impact of Family History on Prostate Cancer Mortality in White Men Undergoing Prostate Specific Antigen Based Screening. Journal of Urology. 2015. 193(1):75-79

145. Chudgar, N. P., Bucciarelli, P. R., Jeffries, E. M., Rizk, N. P., Park, B. J., Adusumilli, P. S., Jones, D. R.. Results of the National Lung Cancer Screening Trial: Where Are We Now?. Thoracic Surgery Clinics. 2015. 25(2):145-153

146. Lujan, M., Paez, A., Angulo, J. C., Granados, R., Nevado, M., Torres, G. M., Berenguer, A.. Prostate cancer incidence and mortality in the Spanish section of the European Randomized Study of Screening For Prostate Cancer (ERSPC). Prostate Cancer and Prostatic Diseases. 2014. 17(2):187-191

147. Vedder, M. M., De Bekker-Grob, E. W., Lilja, H. G., Vickers, A. J., Van Leenders, G. J. L. H., Steyerberg, E. W., Roobol, M. J.. The added value of percentage of free to total prostate-specific antigen, PCA3, and a kallikrein panel to the erspc risk calculator for prostate cancer in prescreened men. European Urology. 2014. 66(6):1109-1115

148. Roobol, M. J., Zhu, X., Schroder, F. H., Van Leenders, G. J. L. H., Van Schaik, R. H., Bangma, C. H., Steyerberg, E. W.. A calculator for prostate cancer risk 4 years after an initially negative screen: Findings from erspc rotterdam. European Urology. 2013. 63(4):627-633

149. Schroder, F. H., Hugosson, J., Carlsson, S., Tammela, T., Maattanen, L., Auvinen, A., Kwiatkowski, M., Recker, F., Roobol, M. J.. Screening for prostate cancer decreases the risk of developing metastatic disease: Findings from the European Randomized Study of Screening for Prostate Cancer (ERSPC). European Urology. 2012. 62(5):745-752

150. Lujan, M., Paez, A., Berenguer, A., Rodriguez, J. A.. Mortality due to prostate cancer in the Spanish arm of the European Randomized Study of Screening for Prostate Cancer

(ERSPC). Results after a 15-year follow-up. [Spanish]. Actas Urologicas Espanolas. 2012. 36(7):403-409

151. Kilpel, auml, inen, T. P., Auvinen, A., auml, auml, tt, auml, nen, L., Kujala, P., Ruutu, M., Stenman, U. H., Tammela, T. L. (2010). Results of the three rounds of the Finnish Prostate Cancer Screening Trial--the incidence of advanced cancer is decreased by screening International journal of cancer, 127(7), 1699

152. Labrie, F., Cusan, L., Gomez, J., Levesque, J., Candas, B. (1999). Screening and treatment of localized prostate cancer decreases mortality: first analysis of the first prospective and randomized study on prostate cancer screening Aging male, 2(1), 33

153. Kwiatkowski, M., Huber, A., Moschopulos, M., Lehmann, K., Wernli, M., auml, feli, A., Recker, F. (2004). Prostate cancer screening: results of a prospective trial in Canton Aargau, Switzerland Swiss medical weekly, 134(39-40), 580

154. Roobol, M. J., Kerkhof, M., Schr, ouml, der, F. H., Cuzick, J., Sasieni, P., Hakama, M., Stenman, U. H., Ciatto, S., Nelen, V., Kwiatkowski, M., Lujan, M., Lilja, H., Zappa, M., Denis, L., Recker, F., Berenguer, A., Ruutu, M., Kujala, P., Bangma, C. H., Aus, G., Tammela, T. L., Villers, A., Rebillard, X., Moss, S. M., de Koning, H. J., Hugosson, J., Auvinen, A. (2009). Prostate Cancer Mortality Reduction by Prostate-Specific Antigen-Based Screening Adjusted for Nonattendance and Contamination in the European Randomised Study of Screening for Prostate Cancer (ERSPC) European urology, 56(4), 584

Ineligible outcomes (n=85)

1. Steuber, T., Heidegger, I., Kafka, M., Roeder, M. A., Chun, F., Preisser, F., Palisaar, R. J., Hanske, J., Budaeus, L., Schiess, R., Keller, T., Semjonow, A., Hammerer, P., Manka, L., Ecke, T., Schwentner, C., Ohlmann, C.. PROPOSe: A Real-life Prospective Study of Proclarix, a Novel Blood-based Test to Support Challenging Biopsy Decision-making in Prostate Cancer. European Urology Oncology. 2021. 06:06

2. Rannikko, A., Leht, M., Mirtti, T., Kenttamies, A., Tolonen, T., Rinta-Kiikka, I., Kilpelainen, T. P., Natunen, K., Lilja, H., Lehtimaki, T., Raitanen, J., Kujala, P., Ronkainen, J., Matikainen, M., Petas, A., Taari, K., Tammela, T., Auvinen, A.. Population-based randomized trial of screening for clinically significant prostate cancer ProScreen: a pilot study. BJU International. 2021. 27:27

3. Chiu, P. K., Shen, X., Wang, G., Ho, C. L., Leung, C. H., Ng, C. F., Choi, K. S., Teoh, J. Y.. Enhancement of prostate cancer diagnosis by machine learning techniques: an algorithm development and validation study. Prostate Cancer & Prostatic Diseases. 2021. 15:15

4. Nan, L., Guo, K., Li, M., Wu, Q., Huo, S.. Development and validation of a multi-parameter nomogram for predicting prostate cancer: a retrospective analysis from Handan Central Hospital in China. PeerJ. 2022. 10:e12912

5. Wang, C., Wang, Y. Y., Wang, S. Y., Ding, J. X., Ding, M., Ruan, Y., Wang, X. H., Jing, Y. F., Han, B. M., Xia, S. J., Jiang, C. Y., Zhao, F. J.. Peripheral zone PSA density: a predominant variable to improve prostate cancer detection efficiency in men with PSA higher than 4 ng ml⁻¹. Asian Journal of Andrology. 2021. 23:415-420

6. Kovac, E., Carlsson, S. V., Lilja, H., Hugosson, J., Kattan, M. W., Holmberg, E., Stephenson, A. J.. Association of Baseline Prostate-Specific Antigen Level With Long-term Diagnosis of Clinically Significant Prostate Cancer Among Patients Aged 55 to 60 Years: A Secondary Analysis of a Cohort in the Prostate, Lung, Colorectal, and Ovarian (PLCO) Cancer Screening Trial. JAMA Network Open. 2020. 3:e1919284

7. Halpern, J. A., Oromendia, C., Shoag, J. E., Mittal, S., Cosiano, M. F., Ballman, K. V., Vickers, A. J., Hu, J. C.. Use of Digital Rectal Examination as an Adjunct to Prostate Specific Antigen in the Detection of Clinically Significant Prostate Cancer. Journal of urology. 2018. Vol.199:947-953p

8. Ramos, C. G., Valdevenito, R., Vergara, I., Anabalon, P., Sanchez, C., Fulla, J.. PCA3 sensitivity and specificity for prostate cancer detection in patients with abnormal PSA and/or suspicious digital rectal examination. First Latin American experience. Urologic oncology: seminars and original investigations. 2013. Vol.31:1522-1526p

9. Gronberg, H., Adolfsson, J., Aly, M., Nordstrom, T., Wiklund, P., Brandberg, Y., Thompson, J., Wiklund, F., Lindberg, J., Clements, M., Egevad, L., Eklund, M.. Prostate cancer screening in men aged 50-69 years (STHLM3): a prospective population-based diagnostic study. Lancet oncology. 2015. Vol.16:1667-1676p

10. Pierre-Victor, D., Pinsky, P. F.. Association of Nonadherence to Cancer Screening Examinations With Mortality From Unrelated Causes: a Secondary Analysis of the PLCO Cancer Screening Trial. JAMA internal medicine. 2019. Vol.179:196-203p

11. Wallstrom, J., Geterud, K., Kohestani, K., Maier, S. E., Pihl, C. G., Socratous, A., Stranne, J., Arnsrud-Godtman, R., Mansson, M., Hellstrom, M., Hugosson, J.. Prostate Cancer Screening with Magnetic Resonance Imaging: results from the Second Round of the Gteborg Prostate Cancer Screening 2 Trial. European urology oncology. 2021. #volume#:#pages#

12. Verbeek, J. F. M., Bangma, C. H., Kweldam, C. F., van der Kwast, T. H., Kummerlin, I. P., van Leenders, Gjlh, Roobol, M. J.. Reducing unnecessary biopsies while detecting clinically significant prostate cancer including cribriform growth with the ERSPC Rotterdam risk calculator and 4Kscore. Urologic oncology. 2019. Vol.37:138-144p

13. Boniol, M., Autier, P., Perrin, P., Boyle, P.. Variation of Prostate-specific Antigen Value in Men and Risk of High-grade Prostate Cancer: Analysis of the Prostate, Lung, Colorectal, and Ovarian Cancer Screening Trial Study. Urology. 2015. 85:1117-1122

14. Shelton, J. B., Ochotorena, L., Bennett, C., Shekelle, P., Kwan, L., Skolarus, T., Goldzweig, C.. Reducing PSA-Based Prostate Cancer Screening in Men Aged 75 Years and Older with the Use of Highly Specific Computerized Clinical Decision Support. Journal of General Internal Medicine. 2015. 30:1133-9

15. Belbase, N. P., Agrawal, C. S., Pokharel, P. K., Agrawal, S., Lamsal, M., Shakya, V. C.. Prostate cancer screening in a healthy population cohort in eastern Nepal: an explanatory trial study. Asian Pacific Journal of Cancer Prevention: Apjcp. 2013. 14:2835-8

16. Jochems, S. H. J., Fritz, J., Haggstrom, C., Jarvholm, B., Stattin, P., Stocks, T.. Smoking and Risk of Prostate Cancer and Prostate Cancer Death: A Pooled Study. European Urology.. 2022. #volume#:#pages#

17. Majchrzak, N., Cieslinski, P., Milecki, T., Twardosz, K., Glyda, M., Karmelita-Katulska, K.. Analysis of the usefulness of magnetic resonance imaging and clinical parameters in the detection of prostate cancer in the first systematic biopsy combined with targeted cognitive biopsy. Central European Journal of Urology. 2021. 74(3):321-326

18. Alijaj, N., Pavlovic, B., Martel, P., Rakauskas, A., Cesson, V., Saba, K., Hermanns, T., Oechslin, P., Veit, M., Provenzano, M., Ruschoff, J. H., Brada, M. D., Rupp, N. J., Poyet, C., Derre, L., Valerio, M., Banzola, I., Eberli, D.. Identification of Urine Biomarkers to Improve Eligibility for Prostate Biopsy and Detect High-Grade Prostate Cancer. Cancers. 2022. 14(5) (no pagination):#pages#

19. Zhou, Z., Liang, Z., Zuo, Y., Zhou, Y., Yan, W., Wu, X., Ji, Z., Li, H., Hu, M., Ma, L.. Development of a nomogram combining multiparametric magnetic resonance imaging and PSA-related parameters to enhance the detection of clinically significant cancer across different region. Prostate. 2022. 82(5):556-565

20. Matti, B., Lyndon, M., Zargar-Shoshtari, K.. Ethnic and socio-economic disparities in prostate cancer screening: lessons from New Zealand. BJU International. 2021. 128(S3):11-17

21. Sung, J. J. Y., Luk, A. K. C., Ng, S. S. M., Ng, A. C. F., Chiu, P. K. F., Chan, E. Y. Y., Cheung, P. S. Y., Chu, W. C. W., Wong, S. H., Lam, T. Y. T., Wong, S. Y. S.. Effectiveness of One-Stop Screening for Colorectal, Breast, and Prostate Cancers: A Population-Based Feasibility Study. Frontiers in Oncology. 2021. 11 (no pagination):#pages#

22. Mello-Grand, M., Gregnanin, I., Sacchetto, L., Ostano, P., Zitella, A., Bottoni, G., Oderda, M., Marra, G., Munegato, S., Pardini, B., Naccarati, A., Gasparini, M., Gontero, P., Chiorino, G.. Circulating microRNAs combined with PSA for accurate and non-invasive prostate cancer detection. Carcinogenesis. 2019. 40(2):246-253

23. Ferraro, S., Bussetti, M., Rossi, R. S., Incarbone, G. P., Panteghini, M.. Is pre-biopsy serum prostate specific antigen retesting always justified? A study of the influence of individual and analytical factors on decision making for biopsy referral. Clinica Chimica Acta. 2021. 516:77-82

24. Rani, P., Bhatia, A. S., Singh, H.. Screening of prostate specific antigen (Psa) for the detection of prostate cancer and its prevalence in Jammu Region. European Journal of Molecular and Clinical Medicine. 2020. 7(7):3977-3985

25. Laddha, A., Thomas, A., Nair, D. C., Ravindran, G. C., Pooleri, G. K.. Outcomes of standard 12-core transrectal ultrasound-guided prostate biopsy in biopsy naive Indian men -single center experience. Indian Journal of Urology. 2020. 36(3):179-183

26. Zhu, J., Liang, Z., Song, Y., Yang, Y., Xu, Y., Lu, Y., Hu, R., Ou, N., Zhang, W., Liu, X.. Can the combination of biparametric magnetic resonance imaging and PSA-related indicators predict the prostate biopsy outcome?. Andrologia. 2020. 52(10) (no pagination):#pages#

27. Lin, C. H., Chang, C. W., Li, W. M., Wen, S. C., Huang, S. P., Li, C. C., Wu, W. J., Ke, H. L., Lee, Y. C., Jhan, J. H.. The value of prostate-specific antigen-age volume score in predicting prostate cancer in Taiwan. Urological Science. 2020. 31(4):163-169

28. Matti, B., Zargar-Shoshtari, K.. Opportunistic prostate cancer screening: A population-based analysis. Urologic Oncology: Seminars and Original Investigations. 2020. 38(5):393-400

29. Tuesley, K. M., Jordan, S. J., Siskind, D. J., Kendall, B. J., Kisely, S.. Colorectal, cervical and prostate cancer screening in Australians with severe mental illness: Retrospective nation-wide cohort study. Australian and New Zealand Journal of Psychiatry. 2019. 53(6):550-558

30. Oki, R., Ito, K., Suzuki, R., Fujizuka, Y., Arai, S., Miyazawa, Y., Sekine, Y., Koike, H., Matsui, H., Shibata, Y., Suzuki, K.. Long-term longitudinal changes in baseline PSA distribution and estimated prevalence of prostate cancer in male Japanese participants of population-based PSA screening. International Journal of Cancer. 2018. 143(7):1611-1619

31. Jyoti, S. K., Blacke, C., Patil, P., Amblihalli, V. P., Nicholson, A.. Prostate cancer screening by prostate-specific antigen (PSA); a relevant approach for the small population of the Cayman Islands. Cancer Causes and Control. 2018. 29(1):87-92

32. Hashimoto, T., Ohori, M., Shimodaira, K., Kaburaki, N., Hirasawa, Y., Satake, N., Gondo, T., Nakagami, Y., Namiki, K., Ohno, Y.. Prostate-specific antigen screening impacts on biochemical recurrence in patients with clinically localized prostate cancer. International Journal of Urology. 2018. 25(6):561-567

33. Nordstrom, T., Gronberg, H., Adolfsson, J., Egevad, L., Aly, M., Eklund, M.. Balancing Overdiagnosis and Early Detection of Prostate Cancer using the Stockholm-3 Model. European Urology Focus. 2018. 4(3):385-387

34. Patwardhan, S. K., Patil, B. P., Shelke, U. R., Singh, A. G.. An overview of serum prostatic surface antigen cut points for recommendation of prostatic biopsy. Urology Annals. 2018. 10(1):65-70

35. Pereira-Azevedo, N., Braga, I., Verbeek, J. F. M., Osorio, L., Cavadas, V., Fraga, A., Carrasquinho, E., Cardoso de Oliveira, E., Nieboer, D., Roobol, M. J.. Prospective evaluation on the effect of interobserver variability of digital rectal examination on the performance of the Rotterdam Prostate Cancer Risk Calculator. International Journal of Urology. 2017. 24(12):826-832

36. Jue, J. S., Barboza, M. P., Prakash, N. S., Venkatramani, V., Sinha, V. R., Pavan, N., Nahar, B., Kanabur, P., Ahdoot, M., Dong, Y., Satyanarayana, R., Parekh, D. J., Punnen, S.. Re-examining Prostate-specific Antigen (PSA) Density: Defining the Optimal PSA Range and Patients for Using PSA Density to Predict Prostate Cancer Using Extended Template Biopsy. Urology. 2017. 105:123-128

37. Tosoian, J. J., Alam, R., Gergis, C., Narang, A., Radwan, N., Robertson, S., McNutt, T., Ross, A. E., Song, D. Y., DeWeese, T. L., Tran, P. T., Walsh, P. C.. Unscreened older men diagnosed with prostate cancer are at increased risk of aggressive disease. Prostate Cancer and Prostatic Diseases. 2017. 20(2):193-196

38. Washino, S., Okochi, T., Saito, K., Konishi, T., Hirai, M., Kobayashi, Y., Miyagawa, T.. Combination of prostate imaging reporting and data system (PI-RADS) score and prostate-specific antigen (PSA) density predicts biopsy outcome in prostate biopsy naive patients. BJU International. 2017. 119(2):225-233

39. Salido-Guadarrama, A. I., Morales-Montor, J. G., Rangel-Escareno, C., Langley, E., Peralta-Zaragoza, O., Colin, J. L. C., Rodriguez-Dorantes, M.. Urinary microRNA-based signature improves accuracy of detection of clinically relevant prostate cancer within the prostate-specific antigen grey zone. Molecular Medicine Reports. 2016. 13(6):4549-4560

40. Park, S. Y., Haiman, C. A., Cheng, I., Park, S. L., Wilkens, L. R., Kolonel, L. N., Le Marchand, L., Henderson, B. E.. Racial/ethnic differences in lifestyle-related factors and prostate cancer risk: the Multiethnic Cohort Study. Cancer Causes and Control. 2015. 26(10):1507-1515

41. Rais-Bahrami, S., Siddiqui, M. M., Vourganti, S., Turkbey, B., Rastinehad, A. R., Stamatakis, L., Truong, H., Walton-Diaz, A., Hoang, A. N., Nix, J. W., Merino, M. J., Wood, B. J., Simon, R. M., Choyke, P. L., Pinto, P. A.. Diagnostic value of biparametric magnetic resonance imaging (MRI) as an adjunct to prostate-specific antigen (PSA)-based detection of prostate cancer in men without prior biopsies. BJU International. 2015. 115(3):381-388

42. Saltzman, A. F., Luo, S., Scherrer, J. F., Carson, K. D., Grubb, R. L., Hudson, M. A.. Earlier prostate-specific antigen testing in African American men-Clinical support for the recommendation. Urologic Oncology: Seminars and Original Investigations. 2015. 33(7):e9-330

43. Lin, Y. R., Wei, X. H., Uhlman, M., Lin, X. T., Wu, S. F., Diao, P. F., Xie, H. Q., Xie, K. J., Tang, P.. PSA density improves the rate of prostate cancer detection in Chinese men with a PSA between 2.5-10.0 ng ml⁻¹ and 10.1-20.0 ng ml⁻¹ : A multicenter study. Asian Journal of Andrology. 2015. 17(3):503-507

44. Nowroozi, M., Ayati, M., Jamshidian, H., Arbab, A., Ghorbani, H., Amini, E., Hakima, H., Salehi, S., Ghadian, A.. Transition zone prostate specific antigen density improves prostate cancer detection in iranian men. Nephro-Urology Monthly. 2015. 7(2) (no pagination):#pages#

45. Goodwin, J. S., Jaramillo, E., Yang, L., Kuo, Y. F., Tan, A.. Is anyone listening? Variation in PSA screening among providers for men 75+ before and after United States preventive services task force recommendations against it: A retrospective cohort study. PLoS ONE. 2014. 9(9) (no pagination):#pages#

46. Wallner, L. P., Frencher, S. K., Hsu, J. W. Y., Chao, C. R., Nichol, M. B., Loo, R. K., Jacobsen, S. J.. Changes in serum prostate-specific antigen levels and the identification of prostate cancer in a large managed care population. BJU International. 2013. 111(8):1245-1252

47. Salami, S. S., Schmidt, F., Laxman, B., Regan, M. M., Rickman, D. S., Scherr, D., Bueti, G., Siddiqui, J., Tomlins, S. A., Wei, J. T., Chinnaiyan, A. M., Rubin, M. A., Sanda, M. G.. Combining urinary detection of TMPRSS2: ERG and PCA3 with serum PSA to predict diagnosis of prostate cancer. Urologic Oncology: Seminars and Original Investigations. 2013. 31(5):566-571

48. Inman, B. A., Zhang, J., Shah, N. D., Denton, B. T.. An examination of the dynamic changes in prostate-specific antigen occurring in a population-based cohort of men over time. BJU International. 2012. 110(3):375-381

49. Faria, E. F., Carvalhal, G. F., Dos Reis, R. B., Tobias-Machado, M., Vieira, R. A. C., Reis, L. O., Nogueira, L., MacHado, R. D., Freitas Jr, C. H., Magnabosco, W. J., Mauad, E. C., Carvalho, A. L.. Use of low free to total PSA ratio in prostate cancer screening: Detection rates, clinical and pathological findings in Brazilian men with serum PSA levels < 4.0 ng/mL. BJU International. 2012. 110(11 B):E653-E657

50. Godtman, R. A., Kollberg, K. S., Pihl, C. G., Mansson, M., Hugosson, J.. "The Association Between Age, Prostate Cancer Risk, and Higher Gleason Score in a Long-term Screening Program: Results from the Goteborg-1 Prostate Cancer Screening Trial". European Urology 01 (2022): 01

51. Arsov, C., Albers, P., Herkommer, K., Gschwend, J., Imkamp, F., Peters, I., Kuczyk, M., Hadaschik, B., Kristiansen, G., Schimmoller, L., Antoch, G., Rummeny, E., Wacker, F., Schlemmer, H., Benner, A., Siener, R., Kaaks, R., Becker, N.. "A randomized trial of risk-adapted screening for prostate cancer in young men-Results of the first screening round of the PROBASE trial". International Journal of Cancer 150 (2022): 1861-1869

52. Lundgren, P. O., Kjellman, A., Norming, U., Gustafsson, O.. "Long-Term Outcome of a Single Intervention Population Based Prostate Cancer Screening Study". Journal of urology Vol.200 (2018): 82-88p

53. Carlsson, S., Assel, M., Ulmert, D., Gerdtsson, A., Hugosson, J., Vickers, A., Lilja, H.. "Screening for Prostate Cancer Starting at Age 50-54 Years. A Population-based Cohort Study". European urology Vol.71 (2017): 46-52p

54. Nirei, T., Tabei, T., Sakai, N., Koh, H., Yoshida, M., Fujikawa, A., Ito, H., Tsutsumi, S., Furuhata, S., Noguchi, S., Taguri, M., Kobayashi, K.. "Real-world data in elderly men from Yokosuka City 15 years after introducing prostate-specific antigen-based population screening". Molecular and Clinical Oncology 16(2) (no pagination) (2022): #pages#

55. Patasius, A., Smailyte, G.. "All-cause mortality risk in national prostate cancer cohort: An impact of population-based prostate cancer screening". Journal of Clinical Medicine 10(11) (no pagination) (2021): #pages#

56. Schoenborn, N. L., Sheehan, O. C., Roth, D. L., Cidav, T., Huang, J., Chung, S. E., Zhang, T., Lee, S., Xue, Q. L., Boyd, C. M.. "Association between Receipt of Cancer Screening and All-Cause Mortality in Older Adults". JAMA Network Open (no pagination) (2021): #pages#

57. Tabei, T., Taguri, M., Sakai, N., Koh, H., Yosida, M., Fujikawa, A., Nirei, T., Tsutsumi, S., Ito, H., Furuhata, S., Kawahara, T., Miyoshi, Y., Noguchi, S., Uemura, H., Kobayashi, K.. "Does screening for prostate cancer improve cancer-specific mortality in Asian men? Real-world data in Yokosuka City 15 years after introducing PSA-based population screening". Prostate 80(11) (2020): 824-830

58. Alpert, P. F.. "New Evidence for the Benefit of Prostate-specific Antigen Screening: Data From 400,887 Kaiser Permanente Patients". Urology 118 (2018): 119-126
59. Xu, L., Wang, J., Guo, B., Zhang, H., Wang, K., Wang, D., Dai, C., Zhang, L., Zhao, X.. "Comparison of clinical and survival characteristics between prostate cancer patients of PSA-based screening and clinical diagnosis in China". Oncotarget 9(1) (2018): 428-441

60. Weight, C. J., Narayan, V. M., Smith, D., Kim, S. P., Karnes, R. J.. "The Effects of Population-based Prostate-specific Antigen Screening Beginning at Age 40". Urology 110 (2017): 127-133

61. Goodwin, J. S., Sheffield, K., Li, S., Tan, A.. "Receipt of Cancer Screening Is a Predictor of Life Expectancy". Journal of General Internal Medicine 31(11) (2016): 1308-1314

62. Lavallee, L. T., Binette, A., Witiuk, K., Cnossen, S., Mallick, R., Fergusson, D. A., Momoli, F., Morash, C., Cagiannos, I., Breau, R. H.. "Reducing the Harm of Prostate Cancer Screening: Repeated Prostate-Specific Antigen Testing". Mayo Clinic Proceedings 91(1) (2016): 17-22

63. Sakai, N., Taguri, M., Kobayashi, K., Noguchi, S., Ikeda, S., Koh, H., Satomi, Y., Furuhata, A.. "Clinical outcomes of prostate cancer patients in Yokosuka City, Japan: A comparative study between cases detected by prostate-specific antigen-based screening in Yokosuka and those detected by other means". International Journal of Urology 22(8) (2015): 747-752

64. Mok, Y., Kimm, H., Shin, S. Y., Jee, S. H., Platz, E. A.. "Screening Prostate-specific Antigen Concentration and Prostate Cancer Mortality: The Korean Heart Study". Urology 85(5) (2015): 1111-1116

65. Carlsson, S., Assel, M., Sjoberg, D., Ulmert, D., Hugosson Prof, J., Lilja, H., Vickers, A.. "Influence of blood prostate specific antigen levels at age 60 on benefits and harms of prostate cancer screening: Population based cohort study". BMJ (Online) 348 (no pagination) (2014): #pages#

66. Ciezki, J. P., Reddy, C. A., Kupelian, P. A., Klein, E. A.. "Effect of prostate-specific antigen screening on metastatic disease burden 10 years after diagnosis". Urology 80(2) (2012): 367-373

67. Wallstrom, J., Geterud, K., Kohestani, K., Maier, S. E., Pihl, C. G., Socratous, A., Stranne, J., Arnsrud-Godtman, R., Mansson, M., Hellstrom, M., Hugosson, J.. "Prostate Cancer Screening with Magnetic Resonance Imaging: Results from the Second Round of the Goteborg Prostate Cancer Screening 2 Trial". European Urology Oncology 5 (2022): 54-60

68. Kohestani, K., Mansson, M., Arnsrud Godtman, R., Stranne, J., Wallstrom, J., Carlsson, S., Hellstrom, M., Hugosson, J.. "The GOTEBORG prostate cancer screening 2 trial: a prospective, randomised, population-based prostate cancer screening trial with prostate-specific antigen testing followed by magnetic resonance imaging of the prostate". Scandinavian Journal of Urology 55 (2021): 116-124

69. Nordstrom, T., Discacciati, A., Bergman, M., Clements, M., Aly, M., Annerstedt, M., Glaessgen, A., Carlsson, S., Jaderling, F., Eklund, M., Gronberg, H., group, Sthlm study. "Prostate cancer screening using a combination of risk-prediction, MRI, and targeted prostate biopsies (STHLM3-MRI): a prospective, population-based, randomised, open-label, non-inferiority trial". Lancet Oncology 22 (2021): 1240-1249

70. Josefsson, Andreas, Mansson, Marianne, Kohestani, Kimia, Spyratou, Vasiliki, Wallstrom, Jonas, Hellstrom, Mikael, Lilja, Hans, Vickers, Andrew, Carlsson, Sigrid V., Godtman, Rebecka, Hugosson, Jonas (2024). Performance of 4Kscore as a Reflex Test to Prostate-specific Antigen in the GOTEBORG-2 Prostate Cancer Screening Trial European urology, #volume#(#issue#), #Pages#

71. Krilaviciute, Agne, Kaaks, Rudolf, Seibold, Petra, de Vrieze, Maxime, Lakes, Jale, Radtke, Jan Philipp, Kuczyk, Markus, Harke, Nina N., Debus, Jurgen, Fink, Christoph A., Herkommer, Kathleen, Gschwend, Jurgen E., Meissner, Valentin H., Benner, Axel, Kristiansen, Glen, Hadaschik, Boris, Arsov, Christian, Schimmoller, Lars, Antoch, Gerald, Giesel, Frederik L., Makowski, Marcus, Wacker, Frank, Schlemmer, Heinz-Peter, Becker, Nikolaus, Albers, Peter (2024). Risk-adjusted Screening for Prostate Cancer-Defining the Low-risk Group by Data from the PROBASE Trial European urology, #volume#(#issue#), #Pages#

72. Moller, Fredrik, Mansson, Marianne, Wallstrom, Jonas, Hellstrom, Mikael, Hugosson, Jonas, Arnsrud Godtman, Rebecka (2024). Prostate Cancers in the Prostate-specific Antigen Interval of 1.8-3 ng/ml: Results from the Goteborg-2 Prostate Cancer Screening Trial European urology, #volume#(#issue#), #Pages#

73. Chiarelli, Giuseppe, Davis, Matthew, Stephens, Alex, Cirulli, Giuseppe Ottone, Finati, Marco, Corsi, Nicholas J., Sood, Akshay, Tinsley, Shane, Carrieri, Giuseppe, Briganti, Alberto, Montorsi, Francesco, Lughezzani, Giovanni, Buffi, Nicolo, Rogers, Craig, Abdollah, Firas (2024). Comparison of patient background between a real-world North American cohort and the Goteborg-2 trial International journal of urology : official journal of the Japanese Urological Association, 31(5), 562-567

74. Bjornebo, Lars, Discacciati, Andrea, Falagario, Ugo, Vigneswaran, Hari T., Jaderling, Fredrik, Gronberg, Henrik, Eklund, Martin, Nordstrom, Tobias, Lantz, Anna (2024). Biomarker vs MRI-Enhanced Strategies for Prostate Cancer Screening: The STHLM3-MRI Randomized Clinical Trial JAMA network open, 7(4), e247131

75. Boschheidgen, Matthias, Albers, Peter, Schlemmer, Heinz-Peter, Hellms, Susanne, Bonekamp, David, Sauter, Andreas, Hadaschik, Boris, Krilaviciute, Agne, Radtke, Jan Philipp, Seibold, Petra, Lakes, Jale, Arsov, Christian, Gschwend, Jurgen E., Herkommer, Kathleen, Makowski, Marcus, Kuczyk, Markus A., Wacker, Frank, Harke, Nina, Debus, Jurgen, Korber, Stefan A., Benner, Axel, Kristiansen, Glen, Giesel, Frederik L., Antoch, Gerald, Kaaks, Rudolf, Becker, Nikolaus, Schimmoller, Lars (2024). Multiparametric Magnetic Resonance Imaging in Prostate Cancer Screening at the Age of 45 Years: Results from the First Screening Round of the PROBASE Trial European urology, 85(2), 105-111

76. Krilaviciute, Agne, Becker, Nikolaus, Lakes, Jale, Radtke, Jan Philipp, Kuczyk, Markus, Peters, Inga, Harke, Nina N., Debus, Jurgen, Koerber, Stefan A., Herkommer, Kathleen, Gschwend, Jurgen E., Meissner, Valentin H., Benner, Axel, Seibold, Petra, Kristiansen,

Glen, Hadaschik, Boris, Arsov, Christian, Schimmoller, Lars, Giesel, Frederik Lars, Antoch, Gerald, Makowski, Marcus, Wacker, Frank, Schlemmer, Heinz-Peter, Kaaks, Rudolf, Albers, Peter (2023). Digital Rectal Examination Is Not a Useful Screening Test for Prostate Cancer European urology oncology, 6(6), 566-573

77. Krilaviciute, Agne, Albers, Peter, Lakes, Jale, Radtke, Jan Philipp, Herkommer, Kathleen, Gschwend, Jurgen, Peters, Inga, Kuczyk, Markus, Koerber, Stefan A., Debus, Jurgen, Kristiansen, Glen, Schimmoller, Lars, Antoch, Gerald, Makowski, Marcus, Wacker, Frank, Schlemmer, Heinz, Benner, Axel, Giesel, Frederik, Siener, Roswitha, Arsov, Christian, Hadaschik, Boris, Becker, Nikolaus, Kaaks, Rudolf (2023). Adherence to a risk-adapted screening strategy for prostate cancer: First results of the PROBASE trial International journal of cancer, 152(5), 854-864

78. Hugosson, Jonas, Mansson, Marianne, Wallstrom, Jonas, Axcrona, Ulrika, Carlsson, Sigrid V., Egevad, Lars, Geterud, Kjell, Khatami, Ali, Kohestani, Kimia, Pihl, Carl-Gustaf, Socratous, Andreas, Stranne, Johan, Godtman, Rebecka Arnsrud, Hellstrom, Mikael (2022). Prostate Cancer Screening with PSA and MRI Followed by Targeted Biopsy Only The New England journal of medicine, 387(23), 2126-2137

79. Nam, Robert, Patel, Chirag, Milot, Laurent, Hird, Amanda, Wallis, Christopher, Macinnis, Patrick, Singh, Mala, Emmenegger, Urban, Sherman, Christopher, Haider, Masoom A. (2022). Prostate MRI versus PSA screening for prostate cancer detection (the MVP Study): a randomised clinical trial BMJ open, 12(11), e059482

80. Stinesen Kollberg, K., Holmberg, E., Josefsson, A., Hugosson, J., Arnsrud Godtman, R. (2022). Prostate Specific Antigen and Biopsy Contamination in the Goteborg-1 Randomized, Population-Based, Prostate Cancer Screening Trial The Journal of urology, 208(5), 1018-1027

81. Davik, P., Elschot, M., Frost Bathen, T., Bertilsson, H. (2024). Repeat Prostate-specific Antigen Testing Improves Risk-based Selection of Men for Prostate Biopsy After Magnetic Resonance Imaging European Urology Open Science, 65(#issue#), 21-28

82. Eldred-Evans, D., Tam, H., Sokhi, H., Padhani, A. R., Connor, M., Price, D., Gammon, M., Klimowska-Nassar, N., Burak, P., Day, E., Winkler, M., Fiorentino, F., Ahmed, H. U. (2023). An Evaluation of Screening Pathways Using a Combination of Magnetic Resonance Imaging and Prostate-specific Antigen: Results from the IP1-PROSTAGRAM Study European Urology Oncology, 6(3), 295-302

83. Kageyama, S., Okinaka, Y., Nishizawa, K., Ishitoya, S., Shichiri, Y., Kim, C. J., Iwata, T., Yokokawa, R., Arai, Y., Nishikawa, Z., Soga, H., Ushida, H., Sakano, Y., Naya, Y., Wada, A., Nagasawa, M., Yoshida, T., Narita, M., Kawauchi, A. (2023). Population-based prostate-specific antigen screening for prostate cancer may have an indirect effect on early detection through opportunistic testing in Kusatsu City, Shiga, Japan Molecular and Clinical Oncology, 18(1), 3

84. Auvinen A, Tammela TLJ, Mirtti T, Lilja H, Tolonen T, Kenttämies A, Rinta-Kiikka I, Lehtimäki T, Natunen K, Nevalainen J, Raitanen J, Ronkainen J, van der Kwast T, Riikonen J, Pétas A, Matikainen M, Taari K, Kilpeläinen T, Rannikko AS; ProScreen Trial Investigators. Prostate Cancer Screening With PSA, Kallikrein Panel, and MRI: The

ProScreen Randomized Trial. JAMA. 2024 May 7;331(17):1452-1459. doi: 10.1001/jama.2024.3841.

85. Fredsøe J, Sandahl M, Vedsted P, et al. Results from the PRIMA Trial: Comparison of the STHLM3 Test and Prostate-specific Antigen in General Practice for Detection of Prostate Cancer in a Biopsy-naïve Population. European Urology Oncology. 2023;6(5):484-492. doi:10.1016/j.euo.2023.07.006

PSA or DRE was not used as the screening method (n=36)

1. Pan, J. F., Su, R., Cao, J. Z., Zhao, Z. Y., Ren, D. W., Ye, S. Z., Huang, R. D., Tao, Z. L., Yu, C. L., Jiang, J. H., Ma, Q.. Modified Predictive Model and Nomogram by Incorporating Prebiopsy Biparametric Magnetic Resonance Imaging With Clinical Indicators for Prostate Biopsy Decision Making. Frontiers in Oncology. 2021. 11:740868

2. Falagario, U. G., Silecchia, G., Bruno, S. M., Di Nauta, M., Auciello, M., Sanguedolce, F., Milillo, P., Macarini, L., Selvaggio, O., Carrieri, G., Cormio, L.. Does Multiparametric Magnetic Resonance of Prostate Outperform Risk Calculators in Predicting Prostate Cancer in Biopsy Naive Patients?. Frontiers in Oncology. 2020. 10:603384

3. Ding, Z., Wu, H., Song, D., Tian, H., Ye, X., Liang, W., Jiao, Y., Hu, J., Xu, J., Dong, F.. Development and validation of a nomogram for predicting prostate cancer in men with prostate-specific antigen grey zone based on retrospective analysis of clinical and multi-parameter magnetic resonance imaging/transrectal ultrasound fusion-derived data. Translational Andrology & Urology. 2020. 9:2179-2191

4. Porcaro, A. B., Tafuri, A., Sebben, M., Shakir, A., Novella, G., Pirozzi, M., Processali, T., Rizzetto, R., Amigoni, N., Tiso, L., Cerrato, C., Brunelli, M., Cerruto, M. A., Migliorini, F., Siracusano, S., Artibani, W.. Prostate volume index and prostatic chronic inflammation have an effect on tumor load at baseline random biopsies in patients with normal DRE and PSA values less than 10 ng/ml: results of 564 consecutive cases. Therapeutic Advances in Urology. 2019. 11:1756287219868604

5. van Riel, Lamjg, Jager, A., Meijer, D., Postema, A. W., Smit, R. S., Vis, A. N., de Reijke, T. M., Beerlage, H. P., Oddens, J. R.. Predictors of clinically significant prostate cancer in biopsy-naive and prior negative biopsy men with a negative prostate MRI: improving MRI-based screening with a novel risk calculator. Therapeutic Advances in Urology. 2022. 14:17562872221088536

6. Kinnaird, A., Brisbane, W., Kwan, L., Priester, A., Chuang, R., Barsa, D. E., Delfin, M., Sisk, A., Margolis, D., Felker, E., Hu, J., Marks, L. S.. A prostate cancer risk calculator: Use of clinical and magnetic resonance imaging data to predict biopsy outcome in North American men. Canadian Urological Association Journal. 2022. 16:E161-E166

7. Pye, H., Singh, S., Norris, J. M., Carmona Echeverria, L. M., Stavrinides, V., Grey, A., Dinneen, E., Pilavachi, E., Clemente, J., Heavey, S., Stopka-Farooqui, U., Simpson, B. S., Bonet-Carne, E., Patel, D., Barker, P., Burling, K., Stevens, N., Ng, T., Panagiotaki, E., Hawkes, D., Alexander, D. C., Rodriguez-Justo, M., Haider, A., Freeman, A., Kirkham, A., Atkinson, D., Allen, C., Shaw, G., Beeston, T., Brizmohun Appayya, M., Latifoltojar, A., Johnston, E. W., Emberton, M., Moore, C. M., Ahmed, H. U., Punwani, S., Whitaker, H. C.. Evaluation of PSA and PSA Density in a Multiparametric Magnetic Resonance Imaging-Directed Diagnostic Pathway for Suspected Prostate Cancer: The INNOVATE Trial. Cancers. 2021. 13:20

8. de Moraes, R. P., Pimenta, R., Mori, F. N. C., Dos Santos, G. A., Viana, N. I., Guimaraes, V. R., de Camargo, J. A., Leite, K. R. M., Srougi, M., Nahas, W. C., Reis, S. T.. Tissue expression of MMP-9, TIMP-1, RECK, and miR338-3p in prostate gland: can it predict cancer?. Molecular Biology Research Communications. 2021. 10:149-156

9. Wenzel, M., Welte, M. N., Grossmann, L., Preisser, F., Theissen, L. H., Humke, C., Deuker, M., Bernatz, S., Gild, P., Ahyai, S., Karakiewicz, P. I., Bodelle, B., Kluth, L. A., Chun, F. K. H., Mandel, P., Becker, A.. Multiparametric MRI may Help to Identify Patients With Prostate Cancer in a Contemporary Cohort of Patients With Clinical Bladder Outlet Obstruction Scheduled for Holmium Laser Enucleation of the Prostate (HoLEP). Frontiers in Surgery. 2021. 8:633196

10. Cindolo, L., Bertolo, R., Minervini, A., Sessa, F., Muto, G., Bove, P., Vittori, M., Bozzini, G., Castellan, P., Mugavero, F., Falsaperla, M., Schips, L., Celia, A., Bada, M., Porreca, A., Pastore, A., Al Salhi, Y., Giampaoli, M., Novella, G., Rizzetto, R., Trabacchin, N., Mantica, G., Pini, G., Lombardo, R., Tubaro, A., Antonelli, A., De Nunzio, C.. External validation of Cormio nomogram for predicting all prostate cancers and clinically significant prostate cancers. World journal of urology. 2020. 38(10):2555-2561

11. Zhang, W., Ren, S. C., Shi, X. L., Liu, Y. W., Zhu, Y. S., Jing, T. L., Wang, F. B., Chen, R., Xu, C. L., Wang, H. Q., Wang, H. F., Wang, Y., Liu, B., Li, Y. M., Fang, Z. Y., Guo, F., Lu, X., Shen, D., Gao, X., Hou, J. G., Sun, Y. H.. A novel urinary long non-coding RNA transcript improves diagnostic accuracy in patients undergoing prostate biopsy. Prostate. 2015. Vol.75:653-661p

12. Gershman, B., Van Houten, H. K., Herrin, J., Moreira, D. M., Kim, S. P., Shah, N. D., Karnes, R. J.. Impact of Prostate-specific Antigen (PSA) Screening Trials and Revised PSA Screening Guidelines on Rates of Prostate Biopsy and Postbiopsy Complications. European urology. 2017. Vol.71:55-65p

13. Tam, A. W., Khusid, J., Inoyatov, I., Becerra, A. Z., Davila, J., Chouhan, J. D., Weiss, J. P., Hyacinthe, L. M., McNeil, B. K., Winer, A. G.. Changes observed in prostate biopsy practices in an inner city hospital with a high risk patient population following the 2012 uspstf psa screening recommendations. International Braz J Urol. 2018. 44:697-703

14. Seikkula, H. A., Kaipia, A. J., Rantanen, M. E., Pitkaniemi, J. M., Malila, N. K., Bostrom, P. J.. Stage-specific mortality and survival trends of prostate cancer patients in Finland before and after introduction of PSA. Acta Oncologica. 2017. 56:971-977

15. Shah, N., Ioffe, V., Cherone, S.. Prostate Biopsy Features: A Comparison Between the Pre- and Post-2012 United States Preventive Services Task Force Prostate Cancer Screening Guidelines With Emphasis on African American and Septuagenarian Men. Reviews in Urology. 2019. 21:1-7

16. Shah, N., Ioffe, V., Huebner, T., Hristova, I.. Prostate Biopsy Characteristics: A Comparison Between the Pre- and Post-2012 United States Preventive Services Task Force (USPSTF) Prostate Cancer Screening Guidelines. Reviews in Urology. 2018. 20:77-83

17. Hansen, M., Hamieh, N. M., Markt, S. C., Vaselkiv, J. B., Pernar, C. H., Gonzalez-Feliciano, A. G., Peisch, S., Chowdhury-Paulino, I. M., Rencsok, E. M., Rebbeck, T. R., Platz, E. A., Giovannucci, E. L., Wilson, K. M., Mucci, L. A.. Racial Disparities in Prostate Cancer: Evaluation of Diet, Lifestyle, Family History, and Screening Patterns. Cancer Epidemiology Biomarkers and Prevention. 2022. 31(5):982-990

18. Morote, J., Campistol, M., Celma, A., Regis, L., de Torres, I., Semidey, M. E., Roche, S., Mast, R., Santamaria, A., Planas, J., Trilla, E.. The Efficacy of Proclarix to Select Appropriate Candidates for Magnetic Resonance Imaging and Derived Prostate Biopsies in Men with Suspected Prostate Cancer. World Journal of Men's Health. 2022. 40:#pages#

19. Desai, M. M., Cacciamani, G. E., Gill, K., Zhang, J., Liu, L., Abreu, A., Gill, I. S.. Trends in Incidence of Metastatic Prostate Cancer in the US. JAMA Network Open.. 2022. #volume#:#pages#

20. Omri, N., Alex, S., Jacob, B., Ofer, N.. The additive value of mpMRI on prostate cancer detection: Comparison between patients with and without a suspicious digital rectal examination (DRE). Urologic Oncology: Seminars and Original Investigations. 2021. 39(10):728.e7-728.e11

21. Chiu, S. T., Cheng, Y. T., Pu, Y. S., Lu, Y. C., Hong, J. H., Chung, S. D., Chiang, C. H., Huang, C. Y.. Prostate Health Index Density Outperforms Prostate Health Index in Clinically Significant Prostate Cancer Detection. Frontiers in Oncology. 2021. 11 (no pagination):#pages#

22. Wagaskar, V. G., Sobotka, S., Ratnani, P., Young, J., Lantz, A., Parekh, S., Falagario, U. G., Li, L., Lewis, S., Haines, K., Punnen, S., Wiklund, P., Tewari, A.. A 4K score/MRI-based nomogram for predicting prostate cancer, clinically significant prostate cancer, and unfavorable prostate cancer. Cancer Reports. 2021. 4(4) (no pagination):#pages#

23. Ajami, T., Durruty, J., Mercader, C., Rodriguez, L., Ribal, M. J., Alcaraz, A., Vilaseca, A.. Impact on prostate cancer clinical presentation after non-screening policies at a tertiary-care medical center- a retrospective study. BMC Urology. 2021. 21(1) (no pagination):#pages#

24. Enblad, A. P., Bergengren, O., Andren, O., Larsson, A., Fall, K., Johansson, E., Garmo, H., Bill-Axelson, A.. PSA testing patterns in a large Swedish cohort before the implementation of organized PSA testing. Scandinavian Journal of Urology. 2020. 54(5):376-381

25. Patasius, A., Kaceniene, A., Ulys, A., Stukas, R., Smailyte, G.. Suicide risk among prostate cancer patients before and after the implementation of prostate-specific antigen-based prostate screening in Lithuania in 2006. European Journal of Cancer Prevention. 2020. #volume#:103-107

26. Presti, J., Alexeeff, S., Horton, B., Prausnitz, S., Avins, A. L.. Changes in Prostate Cancer Presentation Following the 2012 USPSTF Screening Statement: Observational Study in a Multispecialty Group Practice. Journal of General Internal Medicine. 2020. 35(5):1368-1374

27. Boesen, L., Norgaard, N., Logager, V., Balslev, I., Bisbjerg, R., Thestrup, K. C., Jakobsen, H., Thomsen, H. S.. Prebiopsy Biparametric Magnetic Resonance Imaging Combined with Prostate-specific Antigen Density in Detecting and Ruling out Gleason 7-10 Prostate Cancer in Biopsy-naive Men. European Urology Oncology. 2019. 2(3):311-319

28. Akarasakul, D., Viriyasiripong, S.. Prostate cancer diagnosis-what to expect in the thai population?. Journal of the Medical Association of Thailand. 2019. 102(7 Supplement 6):58-61

29. Loeb, S., Shin, S. S., Broyles, D. L., Wei, J. T., Sanda, M., Klee, G., Partin, A. W., Sokoll, L., Chan, D. W., Bangma, C. H., van Schaik, R. H. N., Slawin, K. M., Marks, L. S., Catalona, W. J.. Prostate Health Index improves multivariable risk prediction of aggressive prostate cancer. BJU International. 2017. 120(1):61-68

30. Banerji, J. S., Wolff, E. M., Massman, J. D., Odem-Davis, K., Porter, C. R., Corman, J. M.. Prostate Needle Biopsy Outcomes in the Era of the U.S. Preventive Services Task Force Recommendation against Prostate Specific Antigen Based Screening. Journal of Urology. 2016. 195(1):66-73

31. Kanao, K., Komori, O., Nakashima, J., Ohigashi, T., Kikuchi, E., Miyajima, A., Nakagawa, K., Eguchi, S., Oya, M.. Individualized prostate-specific antigen threshold

values to avoid overdiagnosis of prostate cancer and reduce unnecessary biopsy in elderly men. Japanese Journal of Clinical Oncology. 2014. 44(9):852-859

32. Paterson, A. L., Sut, M. K., Khan, A. R., Sharma, H. K.. Prostatic biopsies in selected men aged 75 years and older guide key clinical management decisions. International Urology and Nephrology. 2013. 45(6):1539-1544

33. Numao, N., Yoshida, S., Komai, Y., Ishii, C., Kagawa, M., Kijima, T., Yokoyama, M., Ishioka, J., Matsuoka, Y., Koga, F., Saito, K., Masuda, H., Fujii, Y., Kawakami, S., Kihara, K.. Usefulness of pre-biopsy multiparametric magnetic resonance imaging and clinical variables to reduce initial prostate biopsy in men with suspected clinically localized prostate cancer. Journal of Urology. 2013. 190(2):502-508

34. Rashid, M. M., Alam, A. K. M. K., Habib, A. K. M. K., Rahman, H., Hossain, A. K. M. S., Salam, M. A., Rahman, S.. Efficacy of lower cut off value of serum prostate specific antigen in diagnosis of prostate cancer. Bangladesh Medical Research Council Bulletin. 2012. 38(3):90-93

35. Javali, T. D., Dwivedi, D. K., Kumar, R., Jagannathan, N. R., Thulkar, S., Dinda, A. K.. "Magnetic resonance spectroscopy imaging-directed transrectal ultrasound biopsy increases prostate cancer detection in men with prostate-specific antigen between 4-10 ng/mL and normal digital rectal examination". International Journal of Urology 21 (2014): 257-62

36. Klotz, Laurence, Chin, Joseph, Black, Peter C., Finelli, Antonio, Anidjar, Maurice, Machado, Ashley, Levental, Mark, Ghai, Sangeet, Chang, Silvia D., Patel, Chirag, Kassam, Zahra, Loblaw, Andrew, Kebabdjian, Marlene, Pond, Greg, Haider, Masoom A. (2024). Magnetic Resonance Imaging-Targeted Versus Systematic Prostate Biopsies: 2-year Follow-up of a Prospective Randomized Trial (PRECISE) European urology oncology, 7(3), 456-461

Cohort study published prior to 2012 (n=25)

1. Vickers, A. J., Cronin, A. M., Aus, G., Pihl, C. G., Becker, C., Pettersson, K., Scardino, P. T., Hugosson, J., Lilja, H.. A panel of kallikrein markers can reduce unnecessary biopsy for prostate cancer: data from the European Randomized Study of Prostate Cancer Screening in Göteborg, Sweden. BMC medicine. 2008. 6:#pages#

2. Ito, K., Ichinose, Y., Kubota, Y., Imai, K., Yamanaka, H.. Clinicopathological features of prostate cancer detected by transrectal ultrasonography-guided systematic six-sextant biopsy. International journal of urology. 1997. Vol.4:474-479p

3. Crawford, E. D., Pinsky, P. F., Chia, D., Kramer, B. S., Fagerstrom, R. M., Andriole, G., Reding, D., Gelmann, E. P., Levin, D. L., Gohagan, J. K.. Prostate specific antigen changes as related to the initial prostate specific antigen: data from the prostate, lung,

colorectal and ovarian cancer screening trial. Journal of urology. 2006. Vol.175:1286-90; discussion 1290p

4. Hosseini, S. Y., Moharramzadeh, M., Ghadian, A. R., Hooshyar, H., Lashay, A. R., Safarinejad, M. R.. Population-based screening for prostate cancer by measuring total serum prostate-specific antigen in Iran. International journal of urology. 2007. Vol.14:406-411p

5. Ciatto, S., Bonardi, R., Mazzotta, A., Lombardi, C., Santoni, R., Cardini, S., Zappa, M.. Comparison between 2 techniques of screening for prostatic carcinoma. Rectal exploration and transrectal ultrasonography vs. prostate specific antigen. Radiologia medica. 1994. Vol.88:453-457p

6. Catalona, W. J., Richie, J. P., Ahmann, F. R., Hudson, M. A., Scardino, P. T., Flanigan, R. C., DeKernion, J. B., Ratliff, T. L., Kavoussi, L. R., Dalkin, B. L., Waters, W. B., MacFarlane, M. T., Southwick, P. C.. Comparison of Digital Rectal Examination and Serum Prostate Specific Antigen in the Early Detection of Prostate Cancer: results of a Multicenter Clinical Trial of 6,630 Men. Journal of urology. 2017. Vol.197:S200-S207p

7. Filella, X., Molina, R., Ballesta, A. M., Gil, M. J., Allepuz, C., Rioja, L. A.. Value of PSA (prostate-specific antigen) in the detection of prostate cancer in patients with urological symptoms. Results of a multicentre study. European journal of cancer (Oxford, England :. 1996. Vol.32A:1125-1128p

8. Smith, D. S., Humphrey, P. A., Catalona, W. J.. The early detection of prostate carcinoma with prostate specific antigen: the Washington University experience. Cancer. 1997. Vol.80:1852-1856p

9. Gion, M., Mione, R., Barioli, P., Barichello, M., Zattoni, F., Prayer-Galetti, T., Plebani, M., Aimo, G., Terrone, C., Manferrari, F., Madeddu, G., Caberlotto, L., Fandella, A., Pianon, C., Vianello, L., Amoroso, B.. Clinical evaluation of percent free prostate-specific antigen using the AxSYM system in the best analytical scenario. European urology. 2000. Vol.37:460-469p

10. Ciatto, S., Bonardi, R., Mazzotta, A., Lombardi, C., Santoni, R., Cardini, S., Zappa, M.. Comparing two modalities of screening for prostate cancer: digital rectal examination + transrectal ultrasonography vs. prostate-specific antigen. Tumori. 1995. Vol.81:225-229p

11. aacute;ez, A., Luj, aacute;n, M., Llanes, L., Romero, I., de la Cal, M. A., Miravalles, E., Berenguer, A.. PSA-use in a Spanish industrial area. European urology. 2002. Vol.41:162-166p

12. Lujan, M., Paez, A., Miravalles, E., Fernandez, I., Llanes, L., Berenguer, A.. Prostate cancer detection is also relevant in low prostate specific antigen ranges. European urology. 2004. Vol.45:155-159p

13. van den Bergh, R. C., Roobol, M. J., Wolters, T., van Leeuwen, P. J., Schr, ouml;der, F. H.. The Prostate Cancer Prevention Trial and European Randomized Study of Screening for Prostate Cancer risk calculators indicating a positive prostate biopsy: a comparison. BJU international. 2008. Vol.102:1068-1073p

14. Pashayan, N., Pharoah, P., Neal, D. E., Hamdy, F., Donovan, J., Martin, R. M., Greenberg, D., Duffy, S. W.. Stage shift in PSA-detected prostate cancers - effect modification by Gleason score. Journal of medical screening. 2009. Vol.16:98-101p

15. Wolters, T., Roobol, M. J., Bangma, C. H., Schr, ouml;der, F. H.. Is prostate-specific antigen velocity selective for clinically significant prostate cancer in screening? European Randomized Study of Screening for Prostate Cancer (Rotterdam). European urology. 2009. Vol.55:385-392p

16. Turner, E. L., Lane, J. A., Metcalfe, C., Down, L., Donovan, J. L., Hamdy, F., Neal, D., Vedhara, K.. Psychological distress and prostate specific antigen levels in men with and without prostate cancer. Brain, behavior, and immunity. 2009. Vol.23:1073-1078p

17. van Leeuwen, P. J., Connolly, D., Gavin, A., Roobol, M. J., Black, A., Bangma, C. H., Schr, ouml;der, F. H.. Prostate cancer mortality in screen and clinically detected prostate cancer: estimating the screening benefit. European journal of cancer (Oxford, England :. 2010. Vol.46:377-383p

18. Cavadas, V., Os, oacute;rio, L., Sabell, F., Teves, F., Branco, F., Silva-Ramos, M.. Prostate cancer prevention trial and European randomized study of screening for prostate cancer risk calculators: a performance comparison in a contemporary screened cohort. European urology. 2010. Vol.58:551-558p

19. Roobol, M. J., Schr, ouml;der, F. H., van Leeuwen, P., Wolters, T., van den Bergh, R. C., van Leenders, G. J., Hessels, D.. Performance of the prostate cancer antigen 3 (PCA3) gene and prostate-specific antigen in prescreened men: exploring the value of PCA3 for a first-line diagnostic test. European urology. 2010. Vol.58:475-481p

20. Pedersen, K. V., Carlsson, P., Varenhorst, E., ouml;fman, O., Berglund, K.. Screening for carcinoma of the prostate by digital rectal examination in a randomly selected population. BMJ (Clinical research ed.). 1990. Vol.300:1041-1044p

21. Chadwick, D. J., Kemple, T., Astley, J. P., MacIver, A. G., Gillatt, D. A., Abrams, P., Gingell, J. C.. Pilot study of screening for prostate cancer in general practice. Lancet (london, england). 1991. Vol.338:613-616p

22. Van Leeuwen, P. J., Connolly, D., Tammela, T. L. J., Auvinen, A., Kranse, R., Roobol, M. J., Schroder, F. H., Gavin, A.. Balancing the harms and benefits of early detection of prostate cancer. Cancer. 2010. 116(20):4857-4865

23. Pashayan, N., Duffy, S. W., Pharoah, P., Greenberg, D., Donovan, J., Martin, R. M., Hamdy, F., Neal, D. E. (2009). Mean sojourn time, overdiagnosis, and reduction in advanced stage prostate cancer due to screening with PSA: implications of sojourn time on screening British journal of cancer, 100(7), 1198

24. Hugosson, J., Aus, G., Becker, C., Carlsson, S., Eriksson, H., Lilja, H., Lodding, P., Tibblin, G. (2000). Would prostate cancer detected by screening with prostate-specific antigen develop into clinical cancer if left undiagnosed? A comparison of two population-based studies in Sweden BJU international, 85(9), 1078

25. Horninger, W., Berger, A., Pelzer, A., Klocker, H., Oberaigner, W., Schonitzer, D., Severi, G., Robertson, C., Boyle, P., Bartsch, G. (2004). Screening for prostate cancer: Updated experience from the Tyrol study Current Urology Reports, 5(3), 220-225

Language other than English or French (n=21)

1. Al-Monajjed, R., Arsov, C., Albers, P.. [Risk-adapted prostate cancer screening-update 2021]. Urologe (Ausg. A). 2021. 60:592-601

2. Zeissig, S. R., Arndt, V., Kraywinkel, K.. Contribution of epidemiology to secondary prevention of cancer. [German]. Onkologe. 2020. 26(5):393-401

3. Albers, P., Arsov, C., Hiester, A., Quentin, M., Schimoller, L., Antoch, G., Rabenalt, R.. PSA-based early detection of prostate cancer. Onkologe. 2016. 22:558-561

4. Terrone, C., De Luca, S., Chiapello, G.. Screening of prostatic carcinoma: pros and cons. Recenti progressi in medicina. 2000. Vol.91:559-561p

5. Marzo Castillejo, M., Cierco Peguera, P., Bonfill Cosp, X.. Prostate cancer screening. Atencion primaria / sociedad española de medicina de familia y comunitaria. 2001. Vol.28:468-471p

6. Nakagawa, S.. Evaluation of mass screening for prostatic cancer. Nippon rinsho. Japanese journal of clinical medicine. 2002. 60:289-293

7. Al-Monajjed, R., Arsov, C., Albers, P.. Risk-adapted prostate cancer screening—update 2021. Der Urologe. 2021. Vol.60:592-601p

8. Torres Zambrano, G., Lujan, Gal, aacute;n, M., Pascual Mateo, C., Garc, iacute;a Tello, A., Rodr, iacute;guez, N., Berenguer, S., aacute;nchez, A.. Preliminary data of the Spanish contribution to the European Randomized Study on Screening of Prostate Cancer (ERSPC). Archivos espanoles de urologia. 2007. Vol.60:737-743p

9. Delgado, Rodr, iacute;guez, M.. Prostate cancer screening and mortality in a European Community trial. Enfermeria clinica. 2010. Vol.20:64-65p

10. Mestrinho, B. V., Gomes, L., de Almeida, J. L., de Almeida, J. C., de Oliveira, R. V.. Does clarifyng the digital rectal examination to the elderly reduce the discomfort in its first execution?. Revista do Colegio Brasileiro de Cirurgioes. 2011. Vol.38:407-411p

11. Schroder, F. H., Roobol, M. J., Bangma, C. H.. Early detection of prostate cancer - Recommendations after 13 years of follow-up in the European randomised study. Nederlands tijdschrift voor geneeskunde. 2015. 159:#pages#

12. Setton, J.. PROBASE study - optimizing PSA screening. Aktuelle Urologie. 2013. Vol.44:431p

13. Nakagawa, S., Nakamura, T., Watanabe, H.. A case-control study on the efficacy and the optimum interval of mass screening for prostatic cancer. Nihon Hinyokika Gakkai zasshi [Japanese journal of urology]. 1998. Vol.89:894-898p

14. Luboldt, H. J., Altwein, J. E., Bichler, K. H., Czaja, D., uuml;sing, J., Fornara, P., ouml;ckel, K. H., uuml;bben, G., Schalkh, auml;user, K., Weissbach, L., Wirth, M., uuml;bben, H.. Early recognition of prostate carcinoma. Initial results of a prospective multicenter study in Germany. Project Group for Early Detection DGU-BDU Laboratory diagnosis Professional Circle. Der Urologe. 1999. Ausg. A. Vol.38:114-123p

15. Tan, S. J., Xu, L. W., Xu, Z., Wu, J. P., Liang, K., Jia, R. P.. [The value of PHI/PCA3 in the early diagnosis of prostate cancer]. Chung-Hua i Hsueh Tsa Chih [Chinese Medical Journal]. 2016. 96:100-3

16. Gion, M.. PSA, prostate cancer screening, clinical practice guidelines and the "wise judge". PSA appeared since its earliest. [Italian]. Biochimica Clinica. 2018. 42(4):321-326

17. Jalon Monzon, A., Escaf Barmadah, S., Vina Alonso, L. M., Jalon Monzon, M.. Current aspects of prostate cancer screening. [Spanish]. Semergen. 2017. 43(5):387-393

18. Moharamzadeh, M., Afandiyev, A. M., Guliyev, A. E.. Evaluation of Prostate Specific Antigen (PSA), for early diagnosis of prostate cancer. Azerbaijan Pharmaceutical and Pharmacotherapy Journal. 2012. 12(1):44-47

19. Radtke, Jan Philipp, Handke, Analena Elisa, Haidl, Friederike, Albers, Peter (2024). [ERSPC trial-prostate-specific antigen (PSA)-based prostate cancer screening in older men] ERSPC-Studie - PSA(prostataspezifisches Antigen)-basiertes Prostatakarzinom-Screening bei alteren Patienten., #volume#(#issue#), #Pages#

20. Lorenz, J. (2023). PSA screening in Sweden: Considerably lower prostate cancer mortality Tumor Diagnostik und Therapie, 44(3), 176

21. Belialova, N. S., Belialov, F. I. (2005). Screening of cancer of the most frequent localizations Klinicheskaia meditsina, 83(10), 4

Patients have previous history or pre-existing prostate cancer (n=14)

1. Sonni, I., Felker, E. R., Lenis, A. T., Sisk, A. E., Bahri, S., Allen-Auerbach, M. S., Armstrong, W. R., Suvannarerg, V., Tubtawee, T., Grogan, T., Elashoff, D., Eiber, M., Raman, S. S., Czernin, J., Reiter, R., Calais, J.. Head-to-head comparison of ⁶⁸Ga-PSMA-11 PET/CT and mpMRI with histopathology gold-standard in the detection, intra-prostatic localization and local extension of primary prostate cancer: results from a prospective single-center imaging trial. Journal of Nuclear Medicine. 2021. 14:14

2. Morote, J., Campistol, M., Triquell, M., Celma, A., Regis, L., de Torres, I., Semidey, M. E., Mast, R., Santamaria, A., Planas, J., Trilla, E.. Improving the Early Detection of Clinically Significant Prostate Cancer in Men in the Challenging Prostate Imaging-Reporting and Data System 3 Category. European Urology Open Science. 2022. 37:38-44

3. Dantanarayana, N. D., Hossack, T., Cozzi, P., Brooks, A., Lau, H., Delprado, W., Patel, M. I.. Men under the age of 55 years with screen detected prostate cancer do not have less significant disease compared to older men in a population of patients in Australia. BMC urology. 2015. 15:#pages#

4. Qiao, E. M., Lynch, J. A., Lee, K. M., Kotha, N. V., Nalawade, V., Voora, R. S., Qian, A. S., Nelson, T. J., Yamoah, K., Garraway, I. P., Stewart, T. F., Parsons, J. K., Rose, B. S.. Evaluating Prostate-Specific Antigen Screening for Young African American Men With Cancer. Journal of the National Cancer Institute. 2022. 114(4):592-599

5. Ozdemir, S., Ersay, A. R., Koc Ozturk, F., Ozdemir, B. S.. Predictive value of standard serum markers for bone metastases in prostate cancer. African Journal of Urology. 2021. 27(1) (no pagination):#pages#

6. Schmanke, K., Okut, H., Ablah, E.. Trends for Stage and Grade Group of Prostate Cancer in the US (2010-2016). Urology. 2021. 149:110-116

7. Kumar, A., Riviere, P., Luterstein, E., Nalawade, V., Vitzthum, L., Sarkar, R. R., Bryant, A. K., Einck, J. P., Mundt, A. J., Murphy, J. D., Rose, B. S.. Associations among statins, preventive care, and prostate cancer mortality. Prostate Cancer and Prostatic Diseases. 2020. 23(3):475-485

8. Ried, K., Tamanna, T., Matthews, S., Eng, P., Sali, A.. New Screening Test Improves Detection of Prostate Cancer Using Circulating Tumor Cells and Prostate-Specific Markers. Frontiers in Oncology. 2020. 10 (no pagination):#pages#

9. Xu, Y., Yu, Q., Liu, Y.. Serum relaxin-2 as a novel biomarker for prostate cancer. British Journal of Biomedical Science. 2018. 75(3):145-148

10. Leung, A. K., Hugar, L., Patil, D., Wong, L., Carthon, B., Carney, K. J., Birdsong, G., Moses, K. A., Master, V. A.. The Clinical Course of Patients With Prostate-Specific Antigen >=100ng/ml: Insight Into a Potential Population for Targeted Prostate-Specific Antigen Screening. Urology. 2018. 117:101-107

11. Fujita, K., Hayashi, T., Matsuzaki, K., Nakata, W., Masuda, M., Kawashima, A., Ujike, T., Nagahara, A., Tsuchiya, M., Kobayashi, Y., Nojima, S., Uemura, M., Morii, E., Miyoshi, E., Nonomura, N.. Decreased fucosylated PSA as a urinary marker for high Gleason score prostate cancer. Oncotarget. 2016. 7(35):56643-56649

12. Riedel, D. J., Cox, E. R., Stafford, K. A., Gilliam, B. L.. Clinical presentation and outcomes of prostate cancer in an urban cohort of predominantly African American, human immunodeficiency virus-infected patients. Urology. 2015. 85(2):415-422

13. Leidinger, P., Keller, A., Milchram, L., Harz, C., Hart, M., Werth, A., Lenhof, H. P., Weinhausel, A., Keck, B., Wullich, B., Ludwig, N., Meese, E.. Combination of autoantibody signature with PSA level enables a highly accurate blood-based differentiation of prostate cancer patients from patients with benign prostatic hyperplasia. PLoS ONE. 2015. 10(6) (no pagination):#pages#

14. Pashtan, I., Chen, M. H., D'Amico, A. V.. The impact of PSA and digital rectal examination on the risk of prostate cancer specific mortality in men with a PSA level <2.5ng/ml. Cancer Epidemiology. 2014. 38(5):613-618

Patients were selected based on known condition or risk factor (n=8)

1. Abdel-Rahman, O.. Prostate Cancer Incidence and Mortality in Relationship to Family History of Prostate Cancer; Findings From The PLCO Trial. Clinical Genitourinary Cancer. 2019. 17:e837-e844

2. Sessine, M. S., Das, S., Park, B., Salami, S. S., Kaffenberger, S. D., Kasputis, A., Solorzano, M., Luke, M., Vince, R. A., Kaye, D. R., Borza, T., Stoffel, E. M., Cobain, E.,

Merajver, S. D., Jacobs, M. F., Milliron, K. J., Caba, L., van Neste, L., Mondul, A. M., Morgan, T. M.. Initial Findings from a High Genetic Risk Prostate Cancer Clinic. Urology. 2021. 156:96-103

3. Bancroft, E. K., Saya, S., Page, E. C., Myhill, K., Thomas, S., Pope, J., Chamberlain, A., Hart, R., Glover, W., Cook, J., Rosario, D. J., Helfand, B. T., Hutten Selkirk, C., Davidson, R., Longmuir, M., Eccles, D. M., Gadea, N., Brewer, C., Barwell, J., Salinas, M., Greenhalgh, L., Tischkowitz, M., Henderson, A., Evans, D. G., Buys, S. S., Eeles, R. A., Aaronson, N. K.. Psychosocial impact of undergoing prostate cancer screening for men with BRCA1 or BRCA2 mutations. BJU International. 2019. 123(2):284-292

4. Page, E. C., Bancroft, E. K., Brook, M. N., Assel, M., Hassan Al Battat, M., Thomas, S., Taylor, N., Chamberlain, A., Pope, J., Raghallaigh, H. N., Evans, D. G., Rothwell, J., Maehle, L., Grindedal, E. M., James, P., Mascarenhas, L., McKinley, J., Side, L., Thomas, T., van Asperen, C., Vasen, H., Kiemeney, L. A., Ringelberg, J., Jensen, T. D., Osther, P. J. S., Helfand, B. T., Genova, E., Oldenburg, R. A., Cybulski, C., Wokolorczyk, D., Ong, K. R., Huber, C., Lam, J., Taylor, L., Salinas, M., Feliubadalo, L., Oosterwijk, J. C., van Zelst-Stams, W., Cook, J., Rosario, D. J., Domchek, S., Powers, J., Buys, S., O'Toole, K., Ausems, M. G. E. M., Schmutzler, R. K., Rhiem, K., Izatt, L., Tripathi, V., Teixeira, M. R., Cardoso, M., Foulkes, W. D., Aprikian, A., van Randeraad, H., Davidson, R., Longmuir, M., Ruijs, M. W. G., Helderman van den Enden, A. T. J. M., Adank, M., Williams, R., Andrews, L., Murphy, D. G., Halliday, D., Walker, L., Liljegren, A., Carlsson, S., Azzabi, A., Jobson, I., Morton, C., Shackleton, K., Snape, K., Hanson, H., Harris, M., Tischkowitz, M., Taylor, A., Kirk, J., Susman, R., Chen-Shtoyerman, R., Spigelman, A., Pachter, N., Ahmed, M., Ramon y Cajal, T., Zgajnar, J., Brewer, C., Gadea, N., Brady, A. F., van Os, T., Gallagher, D., Johannsson, O., Donaldson, A., Barwell, J., Nicolai, N., Friedman, E., Obeid, E., Greenhalgh, L., Murthy, V., Copakova, L., Saya, S., McGrath, J., Cooke, P., Ronlund, K., Richardson, K., Henderson, A., Teo, S. H., Arun, B., Kast, K., Dias, A., Aaronson, N. K., Ardern-Jones, A., Bangma, C. H., Castro, E., Dearnaley, D., Eccles, D. M., Tricker, K., Eyfjord, J., Falconer, A., Foster, C., Gronberg, H., Hamdy, F. C., Stefansdottir, V., Khoo, V., Lindeman, G. J., Lubinski, J., Axcrona, K., Mikropoulos, C., Mitra, A., Moynihan, C., Rennert, G., Suri, M., Wilson, P., Dudderidge, T., Offman, J., Kote-Jarai, Z., Vickers, A., Lilja, H., Eeles, R. A.. Interim Results from the IMPACT Study: Evidence for Prostate-specific Antigen Screening in BRCA2 Mutation Carriers. European Urology. 2019. 76(6):831-842

5. Vettenranta, A., Murtola, T. J., Raitanen, J., Raittinen, P., Talala, K., Taari, K., Stenman, U. H., Tammela, T. L. J., Auvinen, A.. "Outcomes of Screening for Prostate Cancer Among Men Who Use Statins". JAMA Oncology 8 (2022): 61-68

6. Vettenranta, A., Murtola, T. J., Talala, K., Taari, K., Stenman, U. H., Tammela, T. L. J., Auvinen, A.. "Outcomes of prostate cancer screening among men using antidiabetic medication". Scientific Reports 11 (2021): 7363

7. Pinsky, P. F., Miller, E. A., Zhu, C. S., Prorok, P. C.. "Overall mortality in men and women in the randomized Prostate, Lung, Colorectal, and Ovarian Cancer Screening Trial". Journal of medical screening 26(3) (2019): 127-134

8. Radtke, J. P., Wiesenfarth, M., Kesch, C., Freitag, M. T., Alt, C. D., Celik, K., Distler, F., Roth, W., Wieczorek, K., Stock, C., Duensing, S., Roethke, M. C., Teber, D., Schlemmer, H. P., Hohenfellner, M., Bonekamp, D., Hadaschik, B. A.. "Combined Clinical Parameters and Multiparametric Magnetic Resonance Imaging for Advanced Risk Modeling of Prostate Cancer-Patient-tailored Risk Stratification Can Reduce Unnecessary Biopsies". European urology #volume# (2017): #pages#

Ineligible comparator group (n=5)

1. Pierre-Victor, D., Parnes, H. L., Andriole, G. L., Pinsky, P. F.. "Prostate Cancer Incidence and Mortality Following a Negative Biopsy in a Population Undergoing PSA Screening". Urology 155 (2021): 62-69

2. Landy, R., Houghton, L. C., Berg, C. D., Grubb, R. L., 3rd, Katki, H. A., Black, A.. "Risk of Prostate Cancer-related Death Following a Low PSA Level in the PLCO Trial". Cancer Prevention Research 13 (2020): 367-376

3. Matsugasumi, T., Okihara, K., Tsujimoto, M., Sato, O., Imura, T., Yamada, Y., Fujihara, A., Shiraishi, T., Hongo, F., Ukimura, O.. "Impact of prostate-specific antigen screening on tumor size in patients with prostate cancer in a super-aging district in Kyoto, Japan". International Journal of Clinical Oncology 26(12) (2021): 2303-2309

4. Kobayashi, M., Nukui, A., Kamai, T.. "Psychological impact of serial prostate-specific antigen tests in Japanese men waiting for prostate biopsy". International Journal of Clinical Oncology 22(1) (2017): 174-180

5. Remmers S, Bangma CH, Godtman RA, Carlsson SV, Auvinen A, Tammela TLJ, Denis LJ, Nelen V, Villers A, Rebillard X, Kwiatkowski M, Recker F, Wyler S, Zappa M, Puliti D, Gorini G, Paez A, Lujan M, Nieboer D, Schröder FH, Roobol MJ. Relationship Between Baseline Prostate-specific Antigen on Cancer Detection and Prostate Cancer Death: Long-term Follow-up from the European Randomized Study of Screening for Prostate Cancer. Eur Urol. 2023 Nov;84(5):503-509. doi: 10.1016/j.eururo.2023.03.031. Epub 2023 Apr 21.

Duplicate of already included study (n=1)

1. Hugosson J, Roobol MJ, Månsson M, Tammela TLJ, Zappa M, Nelen V, Kwiatkowski M, Lujan M, Carlsson SV, Talala KM, Lilja H, Denis LJ, Recker F, Paez A, Puliti D, Villers A, Rebillard X, Kilpeläinen TP, Stenman UH, Godtman RA, Stinesen Kollberg K, Moss SM, Kujala P, Taari K, Huber A, van der Kwast T, Heijnsdijk EA, Bangma C, De Koning HJ, Schröder FH, Auvinen A; ERSPC investigators. A 16-yr Follow-up of the European Randomized study of Screening for Prostate Cancer. Eur Urol. 2019 Jul;76(1):43-51. doi: 10.1016/j.eururo.2019.02.009. Epub 2019 Feb 26.

Supplementary File S7: Study characteristics table

Table S1. Overall summary of RCTs

Trial name (country)<br><br>Type of RCT	Cohort	Screening dates	Number invited to screen (intervention)	Number not invited to screen (control)	Age of screening	Screening interval	PSA threshold	Outcomes of interest reported	Risk of bias score
CAP (UK) [1]<br><br>Cluster randomized	N/A	2001 to 2009	189,386	219,439	50-69 years	Single screen	3.0 ng/ml	Prostate cancer-specific mortality, All-cause mortality	Some concerns
ERSPC (Europe)<br><br>Individual randomized	Finland (FinRSPC) [2–5]	1996 to 1999	31,964	48,402	50-74 years 55-69 years (core group)	4 years (3 rounds)	3.0 – 3.9 ng/ml	Overdiagnosis, metastatic cancer, all-cause mortality, prostate cancer mortality, quality of life	High
	France [6]	2001 to 2003	38,474	38,161	55-69 years	4-6 years<br><br>(2 rounds)	3.0 ng/ml	Prostate cancer mortality, all-cause mortality	High
	Netherlands (Rotterdam) [7–9]	1993 to 2000	17,442	17,389	55-69 years	4 years (3 rounds)	3.0 ng/ml	Prostate cancer-specific mortality, metastatic (M+) disease, prostate cancer diagnoses, overdiagnosis.	Some concerns
	Spain [10]	1996 to 1999	2,415	1,861	45-70 years	4 years (2 rounds)	3.0 ng/ml	Prostate cancer-specific mortality, all-cause mortality, advanced prostate cancer	Some concerns
	Sweden (Goteborg) [11]	1995 to 1999	9,945	9,949	50-64 years	2 years	3.4 or 2.9 or 2.5 ng/ml	All-cause mortality, prostate cancer incidence and mortality.	Some concerns
PLCO (USA) [12]<br><br>Individual randomized	N/A	1993 to 2001	38,340	38,343	55-74 years	1 year (6 rounds)	4.0 ng/ml	Prostate cancer-specific mortality, overall mortality, metastatic cancer	High
STHLM3-MRI trial (Sweden) [13,14]<br><br>Individual randomized	N/A	2018-2020	929	603	50-74 years	Single screen	≥ 3.0 ng/ml (PSA) or ≥11% Stockholm3 scores	Clinically significant cancers, Clinically insignificant cancers (overdiagnosis), Biopsy complications (infection, hospitalization)	High

Abbreviations: CAP: Cluster Randomized Trial of PSA Testing for Prostate Cancer

Table S2 Detailed summary of included articles

Study details	Participants and Study Design	Intervention	Control	Outcomes
**Franlund 2022** [11], **Sweden**<br><br>**Trial:** ERSPC (Goteborg)	**Participants:** Men born between January 1, 1930, and December 31, 1944, living in the city of Goteborg, Sweden.<br><br>**Study design**: RCT<br><br>**Follow-up:** 22 years	n=9,945<br>Participants in the screening group were invited every second year until they reached the stop age (median 69 years, range 67-71). Participants with PSA above the corrected WHO cut-off level (3.4 ng/ml in 1995-1998, 2.9 ng/ml 1999-2004 and 2.5 ng/ml after 2004) were further invited to a clinical assessment including prostate biopsy. Men with PSA below the cut-off level or a benign biopsy were re-invited after 2 years.	n=9,949<br>Men in the CG were not invited for PSA screening but were subject to opportunistic PSA testing.	All-cause mortality, prostate cancer incidence and mortality.
**Walter 2021** [2], **Finland**<br><br>**Trial:** ERSPC (FinRSPC)	**Participants:** Men born in 1929–1944 (aged 55, 59, 63, or 67 years at entry) living in the metropolitan areas of Helsinki and Tampere, Finland.<br><br>**Study design**: RCT<br><br>**Follow-up:** 17 years	n=31,964<br>A PSA level 4.0 ng/ml was used as the indication for biopsy. For men with PSA between 3.0 ng/ml and 3.99 ng/ml a digital rectal examination was initially offered as a supplementary test in 1996–1998, and since 1999, free/total PSA ratio was used (with a cut-off of 0.16).	n=48,402<br>No screening tests.	Overdiagnosis<br><br>The “catch-up method” was used to identify when the difference in the cumulative incidence of prostate cancer between the screening and control groups had stabilized, implying that the screening has no further effect. The overdiagnosis rate is define as the relative excess cumulative incidence in the screened group at the point of stabilization.
**Talala 2020** [3]**Ta, Finland**<br><br>**Trial:** ERSPC (FinRSPC)	**Participants:** Men born in 1929–1944 (aged 55, 59, 63, or 67 years at entry) living in the metropolitan areas of Helsinki and Tampere, Finland.<br><br>**Study design**: RCT<br><br>**Follow-up:** 15 years	n=624<br>Men in the screening arm diagnosed with prostate cancer who have completed a HRQOL assessment.	n=411<br>Men in the control arm diagnosed with prostate cancer who have completed a HRQOL assessment.	Health related quality of life using the UCLA Prostate Cancer Index (PCI) and the RAND 36-item health survey.

**Villers 2020** [6], **France**<br><br>**Trial:** ERSPC (French section)	**Participants:** Men between 55 and 69 years and identified through health insurance databases.<br><br>**Study design**: RCT<br><br>**Follow-up:** 9.5 years	n=38,474<br>Men were invited by mail to be screened by PSA testing with two rounds at 4–6-year intervals. Biopsy was recommended if PSA > = 3.0 ng/mL	n=38,161<br>Men in the control group were not provided specific information on PSA testing but could decide independently, in consultation with their physicians to undergo PSA testing.	Prostate cancer incidence, Prostate cancer mortality, all-cause mortality, Contamination (defined as receipt of PSA testing in a random sample of 243 men in the control arm).
**Lujan Galan 2020** [10], **Spain**<br><br>**Trial:** ERSPC	**Participants:** Men between 45 and 70 years of age, registered in the municipalities of Getafe and Parla.<br><br>**Study design**: RCT<br><br>**Follow-up:** 19 years	n=2,415<br>Serum PSA-based screening.	n=1,861<br>Follow-up without the intervention.	Prostate cancer-specific and all-cause mortality rates. Advanced prostate cancer is defined as M + or serum PSA > 100 ng/mL.
**Osses 2019** [7], **Netherlands**<br><br>**Trial:** ERSPC	**Participants:** Men aged 55–74 years old selected from the population registry of Rotterdam, without a previous diagnosis of PC, randomized between 1991-1992.<br><br>**Study design**: RCT<br><br>**Follow-up:** 19 years	n=553<br>The screening protocol consisted of PSA, digital rectal examination, and transrectal ultrasound and was offered to all men with a 4-yr interval and applying the upper age limit of 74 yr. (maximum of five consecutive screening rounds).	n=581<br>No screening.	Prostate cancer-specific mortality and metastatic (M+) disease defined as N1 and/or M1 and/or PSA >100 ng/ml, including M+ disease at diagnosis and during follow-up.
**Hugosson 2019** [15], **Europe (multiple)**<br><br>**Trial:** ERSPC	**Participants:** Men aged 55–69 years old from eight European countries.<br><br>**Study design**: RCT<br><br>**Follow-up:** 16 years	n=72,890<br>A uniform PSA screening methods was chosen. Additional screening tools were employed in some centres, such as DRE. Since 1996, most centres used a PSA level of 3.0 ng/ml as the definition of a positive screening test. Men with a	n=89,351<br>No screening.	Prostate cancer mortality, adjusted for nonparticipation and the number of screening rounds attended.

		positive screening test were recommended DRE, transrectal ultrasound of the prostate, and systematic prostate biopsies.		
**Pakarainen 2019** [5], **Finland**<br><br>**Trial:** ERSPC (FinRSPC)	**Participants:** Men born in 1929–1944 (aged 55, 59, 63, or 67 years at entry) living in the metropolitan areas of Helsinki and Tampere, Finland.<br><br>**Study design**: RCT<br><br>**Follow-up:** 17 years	n=31,967<br>Repeated PSA-based screening. The men in the screening arm were divided into time-dependent states according to the number of screening rounds attended (screened once, twice, three times, or not at all).	n=48,282<br>No screening.	Prostate cancer–related deaths and overall mortality.
**Lindberg 2019** [4], **Finland**<br><br>**Trial:** ERSPC (FinRSPC)	**Participants:** Men born in 1929–1944 (aged 55, 59, 63, or 67 years at entry) living in the metropolitan areas of Helsinki and Tampere, Finland.<br><br>**Study design**: RCT<br><br>**Follow-up:** 15 years	n=31,867<br>The screening interval was 4 years, and all men were invited regardless of prior attendance. Screening continued up to age 71 years, with men invited three times, except those aged 67 at entry only twice.	n=48,282<br>No screening.	Prostate cancer mortality was examined by age group. PC cases were grouped by Gleason score and risk groups based on TNM stage, Gleason score and PSA values.
**Pinsky 2019** [12], **U.S**<br><br>**Trial:** PLCO	**Participants:** Men aged 55 – 74 at 10 U.S. screening centers, without a history of PLCO cancer and having more than one PSA blood test in the past 3 years.<br><br>**Study design**: RCT<br><br>**Follow-up:** 17 years	n=38,340<br>Men received PSA tests at baseline and annually for 5 more years, and digital rectal exams (DRE) at baseline and annually for 3 more years.	n=38,343<br>Usual care.	Prostate cancer-specific mortality, overall mortality, prostate cancer incidence and prostate cancer characteristics (Gleason score, metastatic vs non-metastatic).
**Hogenhout 2024** [8], **Netherlands**<br><br>**Trial:** ERSPC	**Participants:** Men in the core age group of 55-69 were included.	n=17,442<br>Screening consisted of PSA testing with a 4-yr interval and an upper age limit of 74 yr (maximum of five	n=17,389<br>No screening.	PSA-based screening on the number of prostate cancer diagnoses, tumour characteristics, treatments,

(Rotterdam)	**Study design**: RCT<br><br>**Follow-up:** 10 years	consecutive screening rounds). In general, a PSA level of ≥3.0 ng/ml triggered transrectal ultrasonography–guided biopsy.		cumulative incidence of disease progression, and overdiagnosis.
**Martin 2024** [1], **England and Wales**<br><br>**Trial:** Cluster Randomized trial of PSA Testing for Prostate Cancer (CAP)	**Participants:** Men aged 50 to 69 years in each participating randomized general practice were included.<br><br>**Study design**: Cluster RCT<br><br>**Follow-up:** 15 years	n=189,326<br>Men in practices randomized to the intervention received a single invitation for a PSA test after counseling. If the resulting PSA level was 3.0 to 19.9 ng/mL, they were offered 10-core transrectal ultrasonography-guided biopsies.	n=219,395<br>Men in the control arm did not receive a formal invitation for PSA testing as part of this study.	Prostate cancer-specific mortality, All-cause mortality.
**de Vos 2023** [16], **Netherlands and Belgium**<br><br>**Trial:** ERSPC (Rotterdam)	**Participants:** Men aged 55 to 74 years were recruited from the population registry.<br><br>**Study design**: RCT<br><br>**Follow-up:** 21 years	n=38,426<br>Men in the screening arm underwent PSA testing with a 4-yr interval (i.e., maximum of five consecutive screening rounds). Also, a PSA level of 3.0 ng/ml triggered transrectal ultrasonography–guided biopsy.	n=38,305<br>No screening.	Prostate cancer-specific mortality, Incidence of M+ (Metastasis).
**Vickers 2014** [17], **USA**<br><br>The Surveillance, Epidemiology and End Results (SEER) database	**Participants:** Men aged 60-years old were followed until the age of 85 years.<br><br>**Study design**: Cohort study<br><br>**Follow-up:** 8 years	n=Not reported<br>This group included men from the SEER database during the period when PSA screening was widely adopted (1987-1995), and participants in the PCPT and ERSPC trials who underwent PSA testing and subsequent biopsy if indicated.	n= Not reported<br>No screening. Includes the incidence data from SEER before the widespread adoption of PSA screening (1973-1986), and the cohort from the Malmö Preventive Program (MPP), which had very low rates of PSA screening.	Overdiagnosis
**Pasanen 2024** [9], **Finland, Netherlands**<br><br>**Trial:** the European Randomised Study of	**Participants:** This study included men from the three largest ERSPC centres: Finland, the Netherlands and Sweden. Eligible men were	n=55,209<br>The primary screening test was serum PSA determination. The screening interval was 4 years in Finland (3 rounds) and the	n=71,618<br>Men in the control group were not contacted to screen.	Prostate cancer mortality

Screening for Prostate Cancer (ERSPC) centres, Finland, the Netherlands and Sweden.	aged 55, 59, 63 or 67 years at baseline in Finland, 55–74 years in the Netherlands and 50–69 years in Sweden<br><br>**Study design**: RCT<br><br>**Follow-up:** 16 years	Netherlands (4 rounds), and 2 years (8 rounds) in Sweden.		
**Eklund 2021** [14], **Sweden**<br><br>**Trial:** STHLM3-MRI trial	**Participants:** men aged 50 to 74 years from the general population with PSA level of 3 ng per milliliter or greater.<br><br>**Study design**: RCT<br><br>**Follow-up**: All participants were followed for a minimum of 200 days after receiving PSA test results. Men who underwent biopsy were followed for at least 30 days after the biopsy	n = 929<br>Participants in the experimental group underwent MRI first, and only those with suspicious MRI findings (PI-RADS score ≥ 3) had both MRI-targeted biopsy and standard biopsy. If no clinically significant lesions were identified, biopsies were not performed except in cases of Stockholm3 test scores of 25% or greater (i.e., indicative of a high risk of clinically significant cancer).	n = 603<br>Participants in the standard biopsy group underwent standard transrectal ultrasonography–guided prostate biopsies to obtain 10 to 12 biopsy cores from the peripheral zone of the prostate (apical, midgland, and base)	Clinically significant cancers, clinically insignificant cancers (overdiagnosis), Biopsy complications (infection, hospitalization)
**Nordstrom 2021** [13], **Sweden**<br><br>**Trial:** STHLM3-MRI trial	**Participants:** men aged 50 to 74 years from the general population with PSA level of 3 ng per milliliter or greater or a Stockholm3 of 0.11 or higher.<br><br>**Study design**: RCT<br><br>**Follow-up**: Men who underwent biopsy were followed for at least 30 days after the biopsy	n = 1372<br>Participants allocated to the experimental group received MRI using a 1.5-Tesla (T) Magnetom (Siemens Healthcare, Erlangen, Germany) or a 3T Signa scanner (GE Healthcare, Chicago, IL, USA), without the use of an endorectal coil. In patients undergoing MRI-targeted biopsies, a 10–12 core systematic biopsy procedure was also done in the same session	n = 921<br>Participants allocated to the standard group were referred for systematic prostate biopsy, which was done by experienced urologists using a template of 10–12 biopsy cores taken from the peripheral zone of the prostate (apical, mid gland, and base), according to national guidelines	Clinically significant cancers, clinically insignificant cancers, Biopsy complications (infection)

Supplementary File S8: Risk of Bias Summary

Table S1. Risk of bias summary for included randomized controlled trials

Trial	Randomization process	Deviations from intended interventions	Missing outcome data	Measurement of the outcome	Selection of the reported results	Overall bias
ERSPC Finland	Some concerns	High	Low	Low	Low	High
ERSPC France	Some concerns	High	Low	Low	Low	High
ERSPC Netherlands	Some concerns	Some concerns	Low	Low	Low	Some concerns
ERSPC Spain	Some concerns	Some concerns	Low	Low	Low	Some concerns
ERSPC Sweden	Some concerns	Some concerns	Low	Low	Low	Some concerns
PLCO	Low	High	Low	Low	Low	High
CAP	Low	Some concerns	Low	Low	Low	Some concerns
STHLM3-MRI Trial	Some concerns	High	Low	Low	Low	High

Table S2. Risk of bias summary for included non-randomized study

Checklist Item[1]	Vickers 2014
Were the two groups similar and recruited from the same population?	no
Were the exposures measured similarly to assign people to both exposed and unexposed groups?	no
Was the exposure measured in a valid and reliable way?	yes
Were confounding factors identified?	no
Were strategies to deal with confounding factors stated?	yes
Were the groups/participants free of the outcome at the start of the study (or at the moment of exposure)?	yes
Were the outcomes measured in a valid and reliable way?	yes
Was the follow up time reported and sufficient to be long enough for outcomes to occur? (at least 10-15 years)	yes
Was follow up complete, and if not, were the reasons to loss to follow up described and explored?	unclear
Were strategies to address incomplete follow up utilized?	unclear
Was appropriate statistical analysis used?	yes
Overall risk of bias	

[1]Joanna Briggs Institute (JBI) Critical Appraisal Checklist for Cohort Studies

Supplementary File S9: GRADE Evidence Profile Tables and Forest Plots

Table S1: KQ1 Prostate cancer mortality (RCTs stratified by age)

Invited to screen vs not invited to screen

Bibliography: de Vos et al., 2023, Franlund et al., 2022, Hugosson 2019 et al., 2019, Martin et al., 2024, Pakaraine et al., 2019, Pinsky et al., 2019

Certainty assessment							№ of patients		Effect		Certainty
**№ of studies**	**Study design**	**Risk of bias**	**Inconsistency**	**Indirectness**	**Imprecision**	**Other considerations**	**screening**	**no screening**	**Relative (95% CI)**	**Absolute (95% CI)**	
**All ages (follow-up: range 9.5 years to 22 years)**											
7	randomised trials	very serious[a]	not serious	not serious	not serious	none	2754/401826 (0.7%)	3553/464397 (0.8%)	**RR 0.88** (0.81 to 0.95)	**0.96 fewer per 1,000** (from 1.52 fewer to 0.40 fewer)	⊕⊕◯◯ Low[a]
**Subgroup: 50-59 years (follow-up: range 9.5 years to 16 years)**											
4	randomised trials	very serious[b]	not serious	not serious	very serious[c]	none	162/42481 (0.4%)	255/52172 (0.5%)	**RR 0.75** (0.51 to 1.10)	**1.25 fewer per 1,000** (from 2.45 fewer to 0.5 more)	⊕◯◯◯ Very low[b,c]
**Subgroup: 60-69 years (follow-up: range 9.5 years to 16 years)**											
4	randomised trials	very serious[a]	not serious	not serious	not serious	none	350/51202 (0.7%)	506/57606 (0.9%)	**RR 0.86** (0.70 to 1.04)	**1.26 fewer per 1,000** (from 2.70 fewer to 0.36 more)	⊕⊕◯◯ Low[a]

Certainty assessment							№ of patients		Effect		Certainty
№ of studies	Study design	Risk of bias	Inconsistency	Indirectness	Imprecision	Other considerations	screening	no screening	Relative (95% CI)	Absolute (95% CI)	
**Subgroup: ≥70 years (follow-up: mean 16 years)**											
1	randomised trials	very serious[d]	not serious	not serious	serious[e]	none	1.68 rate per 1,000 person-years	1.59 rate per 1,000 person-years	**Rate ratio 1.06** (0.80 to 1.41)	**0.09 more per 1,000 person-years**	⨁◯◯◯ Very low[d,e]

CI: confidence interval; **RR:** risk ratio

Explanations

a. Blinding and allocation concealment were not possible. Baseline characteristics were not provided for the ERSPC trial, so the effectiveness of the randomization process could not be evaluated. There were very serious concerns with contamination bias and low participation rate in the ERSPC and PLCO trials, therefore we downgraded twice.

b. Blinding and allocation concealment were not possible. Baseline characteristics were not provided for the ERSPC trial, so the effectiveness of the randomization process could not be evaluated. There were very serious concerns with contamination bias and low participation rate in the ERSPC trial, therefore we downgraded twice.

c. The OIS criterion is met, however the 95% CI overlaps no effect (i.e. CI includes RR of 1.0) and includes an appreciable harm (increase of 25% or more) and an appreciable benefit (decrease of 25% or more). We rated down twice.

d. Blinding and allocation concealment were not possible. Baseline characteristics were not provided for the ERSPC trial, so the effectiveness of the randomization process could not be evaluated. There were very serious concerns with contamination bias and the low participation rate in the ERSPC trials, therefore we downgraded twice.

e. The OIS criterion is met, however the 95% CI overlaps no effect (i.e. CI includes RR of 1.0) and includes an appreciable harm (increase of 25% or more). We rated down by one.

Figure S1. KQ1 Prostate cancer mortality (RCTs) - All ages (follow-up: range 9.5 years to 22 years)

Study or Subgroup	Screening Events	Screening Total	No screening Events	No screening Total	Weight	Risk Ratio M-H, Random, 95% CI
Martin 2024 (CAP)	1199	189326	1451	219395	23.9%	0.96 [0.89, 1.03]
de Vos 2023 (ERSPC Netherlands)	283	20984	341	20916	14.3%	0.83 [0.71, 0.97]
Franlund 2022 (ERSPC Sweden)	112	9945	158	9949	8.3%	0.71 [0.56, 0.90]
Villers 2020 (ERSPC France)	78	38474	75	38161	5.4%	1.03 [0.75, 1.42]
Hugosson 2019 (ERSPC Multiple)	520	72890	793	89351	19.5%	0.80 [0.72, 0.90]
Pakaraine 2019 (ERSPC Finland)	229	31867	383	48282	13.6%	0.91 [0.77, 1.07]
Pinsky 2019 (PLCO)	333	38340	352	38343	15.0%	0.95 [0.81, 1.10]
**Total (95% CI)**		**401826**		**464397**	**100.0%**	**0.88 [0.81, 0.95]**
Total events	2754		3553			

Heterogeneity: Tau² = 0.01; Chi² = 12.57, df = 6 (P = 0.05); I² = 52%

Test for overall effect: Z = 3.10 (P = 0.002)

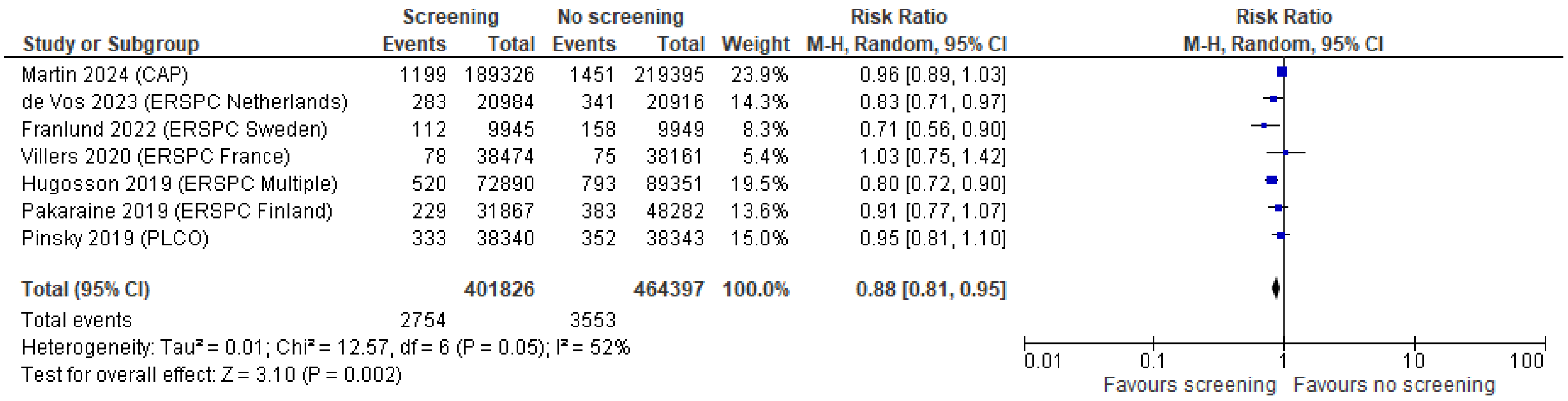

Table S2: KQ1 Prostate cancer mortality (RCTs stratified by screening rounds attended)

Screened cohort vs unscreened cohort

Bibliography: Pakaraine et al., 2019

Certainty assessment							№ of patients‡		Effect‡		Certainty
№ of studies	Study design	Risk of bias	Inconsistency	Indirectness	Imprecision	Other considerations	screening	no screening	Relative* (95% CI)	Absolute (95% CI)	
**0 screening rounds attended (follow-up: 15 years)**											
1	randomised trials	very serious[a]	not serious	not serious	not serious	none	85/30660 (0.3%)<br>**Rate:** 0.73 per 1,000 person-years	383/48282 (0.8%)<br>**Rate:** 0.61 per 1,000 person-years	**HR 1.58** (1.25 to 2.00)	**4.64 more per 1,000** (from 2 more to 8 more)<br>**Rate:** 0.12 more per 1,000 person-years	⨁⨁◯◯ Low[a]
**1 screening round attended (follow-up: 15 years)**											
1	randomised trials	very serious[a]	not serious	not serious	not serious	none	90/22557 (0.4%)<br>**Rate:** 0.70 per 1,000 person-years	383/48282 (0.8%)<br>**Rate:** 0.61 per 1,000 person-years	**HR 1.68** (1.33 to 2.12)	**5.44 more per 1,000** (from 2.64 more to 8.96 more)<br>**Rate:** 0.09 more per 1,000 person-years	⨁⨁◯◯ Low[a]
**2 screening rounds attended (follow-up: 15 years)**											

Certainty assessment							№ of patients‡		Effect‡		Certainty
**№ of studies**	**Study design**	**Risk of bias**	**Inconsistency**	**Indirectness**	**Imprecision**	**Other considerations**	**screening**	**no screening**	**Relative* (95% CI)**	**Absolute (95% CI)**	
1	randomised trials	very serious[a]	not serious	not serious	not serious	none	45/18035 (0.2%)<br>**Rate:** 0.43 per 1,000 person-years	383/48282 (0.8%)<br>**Rate:** 0.61 per 1,000 person-years	**HR 0.48** (0.35 to 0.66)	**4.16 fewer per 1,000** (from 5.20 fewer to 2.72 fewer)<br>**Rate:** 0.18 fewer per 1,000 person-years	⊕⊕◯◯ Low[a]
**3 screening rounds attended (follow-up: 15 years)**											
1	randomised trials	very serious[a]	not serious	not serious	not serious	none	9/10315 (0.1%)<br>**Rate:** 0.14 per 1,000 person-years	383/48282 (0.8%)<br>**Rate:** 0.61 per 1,000 person-years	**HR 0.17** (0.09 to 0.33)	**6.64 fewer per 1,000** (from 7.28 fewer to 5.36 fewer)<br>**Rate:** 0.47 fewer per 1,000 person-years	⊕⊕◯◯ Low[a]

‡Both prevalence and rates are presented as reported by study authors. The absolute effects were calculated in these GRADE tables using the age-adjusted hazard ratios presented by study authors.
*Age-adjusted
CI: confidence interval; **HR:** hazard ratio

Explanations

a. Blinding and allocation concealment were not possible. Baseline characteristics were not provided for the ERSPC trial, so the effectiveness of the randomization process could not be evaluated. There were very serious concerns with contamination bias and low participation rate in the ERSPC trial, therefore we downgraded twice

Table S3: KQ1 All-cause mortality (RCTs)

Invited to screen vs not invited to screen

Bibliography: Franlund et al., 2022, Hugosson 2019 et al., 2019, Lujan Galan et al., 2020, Martin et al., 2024, Villers et al., 2020

Certainty assessment							№ of patients		Effect		Certainty
№ of studies	Study design	Risk of bias	Inconsistency	Indirectness	Imprecision	Other considerations	screening	no screening	Relative (95% CI)	Absolute (95% CI)	
**All ages (follow-up: range 9.5 years to 22 years)**											
5	randomised trials	very serious[a]	not serious	not serious	not serious	none	75035/313050 (24.0%)	84914/358717 (23.7%)	**RR 1.01** (0.98 to 1.05)	**2.37 more per 1,000** (from 4.74 fewer to 11.85 more)	⨁⨁◯◯ Low[a]
**Subgroup: 50-59 years (follow-up: median 9.5 years)**											
1	randomised trials	very serious[a]	not serious	not serious	not serious	none	1383/13690 (10.1%)	1318/13715 (9.6%)	**Rate ratio 1.05** (0.98 to 1.13)	**0.5 more per 1,000 person-years** (from 0.2 fewer to 1.3 more)	⨁⨁◯◯ Low[a]
**Subgroup: 60-69 years (follow-up: range 9.5 years to 16 years)**											

Certainty assessment							№ of patients		Effect		Certainty
№ of studies	Study design	Risk of bias	Inconsistency	Indirectness	Imprecision	Other considerations	screening	no screening	Relative (95% CI)	Absolute (95% CI)	
2	randomised trials	very serious[a]	not serious	not serious	not serious	none	24218/97737 (24.8%)	29075/113881 (25.5%)	**RR 1.00** (0.96 to 1.04)	**0 fewer per 1,000** (from 10 fewer to 10 more)	⊕⊕◯◯ Low[a]

CI: confidence interval; **RR:** risk ratio

Explanations

a. Blinding and allocation concealment were not possible. Baseline characteristics were not provided for the ERSPC trial, so the effectiveness of the randomization process could not be evaluated. There were very serious concerns with contamination bias and low participation rate in the ERSPC trial, therefore we downgraded twice.

Figure S2: KQ1 All-cause mortality (RCTs) - All ages (follow-up: range 9.5 years to 22 years)

	Experimental		Control			Risk Ratio
Study or Subgroup	Events	Total	Events	Total	Weight	M-H, Random, 95% CI
Martin 2024 (CAP)	45084	189326	50336	219395	26.6%	1.04 [1.03, 1.05]
Franlund 2022 (ERSPC Sweden)	3806	9945	3735	9949	20.6%	1.02 [0.98, 1.06]
Lujan Galan 2020 (ERSPC Spain)	544	2415	450	1861	6.6%	0.93 [0.84, 1.04]
Villers 2020 (ERSPC France)	5249	38474	5028	38161	20.5%	1.04 [1.00, 1.07]
Hugosson 2019 (ERSPC Multiple)	20352	72890	25365	89351	25.8%	0.98 [0.97, 1.00]
**Total (95% CI)**		**313050**		**358717**	**100.0%**	**1.01 [0.98, 1.05]**
Total events	75035		84914			

Heterogeneity: Tau² = 0.00; Chi² = 33.54, df = 4 (P < 0.00001); I² = 88%

Test for overall effect: Z = 0.73 (P = 0.47)

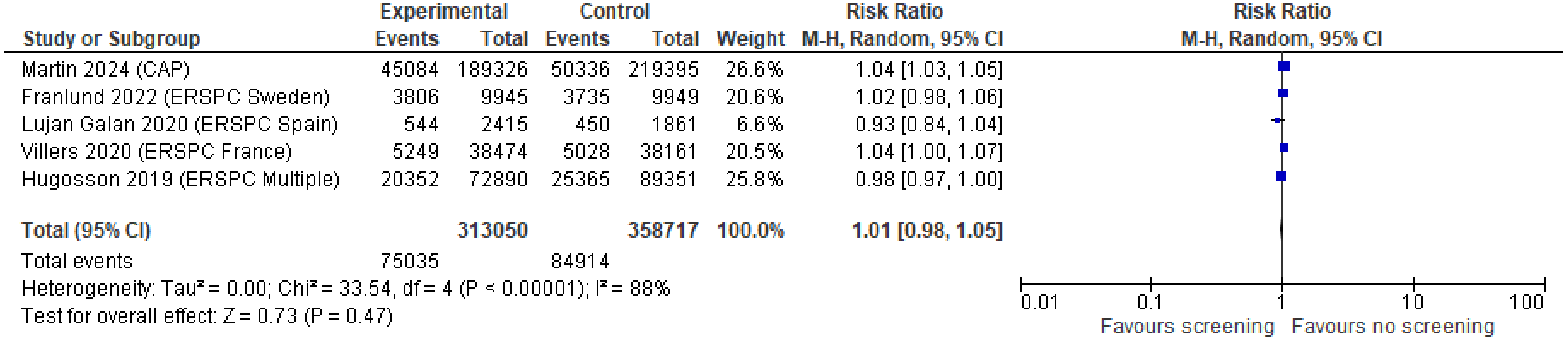

Figure S3: KQ1 All-cause mortality (RCTs) - Subgroup: 60-69 years (follow-up: range 9.5 years to 16 years)

Study or Subgroup	Screening Events	Screening Total	No screening Events	No screening Total	Weight	Risk Ratio M-H, Random, 95% CI
Hugosson 2019 (ERSPC Multiple)	20352	72953	25365	89436	59.9%	0.98 [0.97, 1.00]
Villers 2020 (ERSPC France)	3866	24784	3710	24445	40.1%	1.03 [0.99, 1.07]
**Total (95% CI)**		**97737**		**113881**	**100.0%**	**1.00 [0.96, 1.04]**
Total events	24218		29075			

Heterogeneity: Tau² = 0.00; Chi² = 3.79, df = 1 (P = 0.05); I² = 74%

Test for overall effect: Z = 0.05 (P = 0.96)

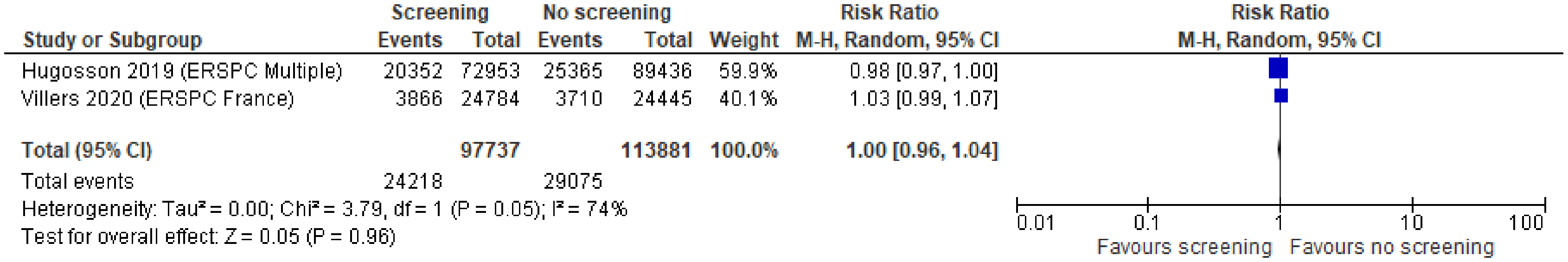

Table S4: KQ1 Metastatic cancer (RCTs)

Invited to screen vs not invited to screen

Bibliography: Lindberg et al., 2019, Lujan Galan et al., 2020, Pinsky et al., 2019, Osses et al., 2018

Certainty assessment							№ of patients		Effect		Certainty
№ of studies	Study design	Risk of bias	Inconsistency	Indirectness	Imprecision	Other considerations	screening	no screening	Relative (95% CI)	Absolute (95% CI)	
**All ages (follow-up: range 15 years to 21 years)**											
4	randomised trials	very serious[a]	serious[b]	not serious	not serious	none	189/55365 (0.3%)	334/63693 (0.5%)	**RR 0.58** (0.35 to 0.98)	**2.1 fewer per 1,000** (from 3.25 fewer to 0.1 fewer)	⨁◯◯◯ Very low[a,b]

CI: confidence interval; **RR:** risk ratio

Explanations

a. Blinding and allocation concealment were not possible. Baseline characteristics were not provided for the ERSPC trial, so the effectiveness of the randomization process could not be evaluated. There were very serious concerns with contamination bias and low participation rate in the ERSPC and PLCO trials, therefore we downgraded twice.

b. I-squared value is 78%, suggesting considerable heterogeneity across studies and variability in RR across studies. We downgraded by one.

Figure S4: KQ1 Metastatic cancer (RCTs) - All ages (follow-up: range 15 years to 21 years)

	Screening		Control			Risk Ratio
**Study or Subgroup**	**Events**	**Total**	**Events**	**Total**	**Weight**	**M-H, Random, 95% CI**
Lujan Galan 2020 (ERSPC Spain)	7	188	5	97	13.7%	0.72 [0.24, 2.22]
Lindberg 2019 (ERSPC Finland)	41	16284	155	24672	32.7%	0.40 [0.28, 0.57]
Pinsky 2019 (PLCO)	134	38340	158	38343	35.5%	0.85 [0.67, 1.07]
Ossess 2018 (ERSPC Netherlands)	7	553	16	581	18.2%	0.46 [0.19, 1.11]
**Total (95% CI)**		**55365**		**63693**	**100.0%**	**0.58 [0.35, 0.98]**
Total events	189		334			

Heterogeneity: $Tau^2 = 0.18$; $Chi^2 = 13.44$, df = 3 (P = 0.004); $I^2 = 78\%$

Test for overall effect: Z = 2.05 (P = 0.04)

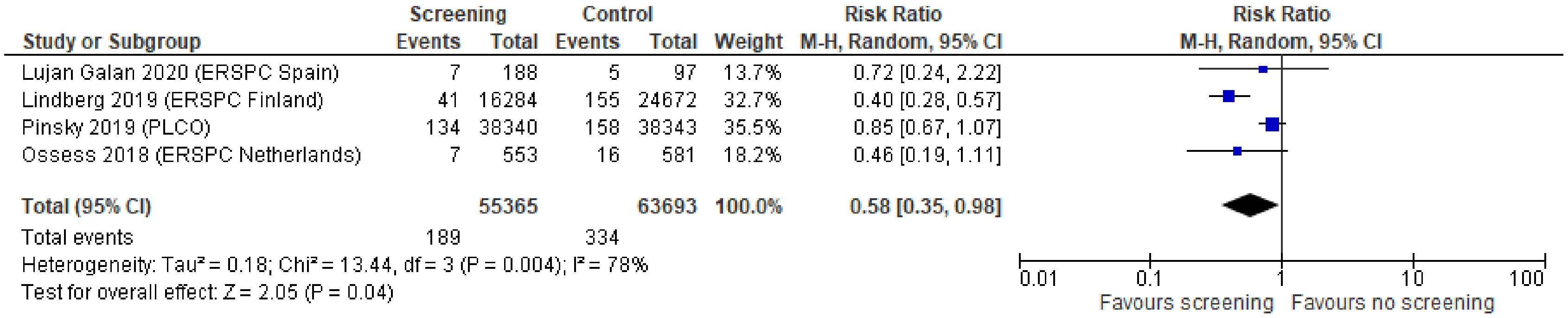

Table S5: KQ1 Overdiagnosis (RCTs stratified by birth cohort)

Invited to screen vs not invited to screen

Bibliography: Walter et al., 2021[2]

Certainty assessment							№ of patients		Effect		Certainty
№ of studies	Study design	Risk of bias	Inconsistency	Indirectness	Imprecision	Other considerations	Screening	No screening	Estimated relative overdiagnosis rate	Cumulative incidence excess of prostate cancer (95% CI)	
**Overdiagnosis[c] (Subgroup: Birth Cohort 1929-1932, 14-year follow-up)**											
1[2]	randomised trials	very serious[a]	not serious	not serious	very serious[b]	none	NR/5816	NR/8604	2.3%	**4 more per 1,000** (from 11 fewer to 19 more)	⨁◯◯◯ Very low[a,b]
**Overdiagnosis[c] (Subgroup: Birth Cohort 1933-1936, 10-year follow-up)**											
1[2]	randomised trials	very serious[a]	not serious	not serious	very serious[b]	none	NR/6555	NR/9765	15.4%	**26 more per 1,000** (from 13 more to 39 more)	⨁◯◯◯ Very low[a,b]
**Overdiagnosis[c] (Subgroup: Birth Cohort 1937-1940, 10-year follow-up)**											

Certainty assessment							№ of patients		Effect		Certainty
**№ of studies**	**Study design**	**Risk of bias**	**Inconsistency**	**Indirectness**	**Imprecision**	**Other considerations**	**Screening**	**No screening**	**Estimated relative overdiagnosis rate**	**Cumulative incidence excess of prostate cancer (95% CI)**	
1[2]	randomised trials	very serious[a]	not serious	not serious	very serious[b]	none	NR/7915	NR/11934	11.4%	**15 more per 1,000** (from 4 more to 26 more)	⊕◯◯◯ Very low[a,b]
**Overdiagnosis[c] (Subgroup: Birth Cohort 1941-1944, 10-year follow-up)**											
1[2]	randomised trials	very serious[a]	not serious	not serious	very serious[b]	none	NR/9911	NR/15008	10.3%	**10 more per 1,000** (from 2 more to 18 more)	⊕◯◯◯ Very low[a,b]

CI: confidence interval

Explanations

a. Blinding and allocation concealment were not possible. Baseline characteristics were not provided for the ERSPC trial, so the effectiveness of the randomization process could not be evaluated. There were very serious concerns with contamination bias and low participation rate in the ERSPC trial, therefore we downgraded it twice.

b. The OIS is met, but the 95% CI crosses both appreciable benefit and harm. We therefore downrated twice.

c. In this study, overdiagnosis was calculated using the “catch-up” method, which identifies the point during follow-up when the cumulative incidence of prostate cancer in screened and control groups stabilizes, indicating that the screening effect has ended. Overdiagnosis was defined as the relative excess cumulative incidence in the screened group at this stabilization point. The relative overdiagnosis rate was calculated as the cumulative difference in incidence at that point, divided by the cumulative incidence in the screened group.

Table S6: KQ1 Overdiagnosis (non-randomized study stratified by age)

Screened cohort vs unscreened cohort

Bibliography: Vickers et al., 2014[17]

Certainty assessment							Impact	Certainty
№ of studies	Study design	Risk of bias	Inconsistency	Indirectness	Imprecision	Other considerations		
**Overdiagnosis[b] (45 to 49 years) (follow-up: 8 years)**								
1[17]	non-randomised studies	very serious[a]	not serious	not serious	not serious	none	One study noted an observed incidence of 10,232 prostate cancer cases with screening and a predicted incidence of 4,277 cases without screening, resulting in 5,955 excess cases (95% CI: 5,803 to 6,106).	⨁◯◯◯ Very low[a]
**Overdiagnosis[b] (50 to 59 years) (follow-up: 8 years)**								
1[17]	non-randomised studies	very serious[a]	not serious	not serious	not serious	none	One study noted an observed incidence of 37,389 prostate cancer cases with screening and a predicted incidence of 17,633 cases without screening for 50 to 54 years, resulting in 19,756 excess cases (95% CI 19,480 to 20,031). For 55 to 59 years, the study noted an observed incidence of 88,783 prostate cancer cases with screening and a predicted incidence of 47,407 cases without screening, resulting in 41,376 excess cases (95% CI 40,977 to 41,774).	⨁◯◯◯ Very low[a]
**Overdiagnosis[b] (60 to 69 years) (follow-up: 8 years)**								

Certainty assessment							Impact	Certainty
№ of studies	Study design	Risk of bias	Inconsistency	Indirectness	Imprecision	Other considerations		
1[17]	non-randomised studies	very serious[a]	not serious	not serious	not serious	none	One study noted an observed incidence of 188,018 prostate cancer cases with screening and a predicted incidence of 111,004 cases without screening for 60 to 64 years, resulting in 77,014 excess cases (95% CI 76,471 to 77,558). For 65 to 69 years, the study noted an observed incidence of 311,865 prostate cancer cases with screening and a predicted incidence of 195,602 cases without screening, resulting in 116,263 excess cases (95% CI 115,596 to 116,930).	⊕◯◯◯ Very low[a]
**Overdiagnosis[b] (70 to 79 years) (follow-up: 8 years)**								
1[17]	non-randomised studies	very serious[a]	not serious	not serious	not serious	none	One study noted an observed incidence of 351,991 prostate cancer cases with screening and a predicted incidence of 238,803 cases without screening for 70 to 74 years, resulting in 113,188 excess cases (95% CI 112,530 to 113,846). For 75 to 79 years, the study noted an observed incidence of 277,274 prostate cancer cases with screening and a predicted incidence of 218,476 cases without screening, resulting in 58,798 excess cases (95% CI 58,324 to 59,273).	⊕◯◯◯ Very low[a]
**Overdiagnosis[b] (80 years and older) (follow-up: 8 years)**								
1[17]	non-randomised studies	very serious[a]	not serious	not serious	not serious	none	One study noted an observed incidence of 261,895 prostate cancer cases with screening and a predicted incidence of 246,002 cases without screening for 80 years and older, resulting in 15,893 excess cases (95% CI 15,646 to 16,140).	⊕◯◯◯ Very low[a]

CI: confidence interval

Explanations

a. There are concerns with adjusting for appropriate confounding variables (i.e., age, lead time bias, or self selection bias) and differences in both groups due to the use of predicted data to estimate overdiagnosis in the unscreened group. The time frame was also deemed not sufficient to be long enough for outcomes to occur (at least 10 to 15 years).
b. To estimate overdiagnosis by age stratum, the study projected what prostate cancer incidence would have been without PSA screening by modeling trends from 1973 to 1986 and extrapolating them through 1995 using linear regression by age and year. They calculated excess cases by subtracting these predicted values from observed diagnoses, standardized to the U.S. population. The estimates were then smoothed by age using locally-weighted scatterplot smoothing (lowess).

Table S7: KQ1 Overdiagnosis (non-randomized study stratified by PSA level at age 60)

Screened cohort vs unscreened cohort

Bibliography: Vickers et al., 2014[17]

Certainty assessment							Absolute risk		Effect		Certainty
№ of studies	Study design	Risk of bias	Inconsistency	Indirectness	Imprecision	Other considerations	Biopsy detected cancer in screened cohort (PCPT)	Clinically diagnosed cancer in unscreened cohort (MPP)	Relative (95% CI)	Absolute (95% CI)	
**Overdiagnosis[b] (PSA level <1.0 ng/ml) (follow-up: 25 years)**											
1[17]	non-randomised studies	very serious[a]	not serious	not serious	not serious	none	12.2%	4.5%	**RR 2.7** (1.9 to 4.2)	**77 more per 1,000** (41 more to 144 more)	⊕◯◯◯ Very low[a]
**Overdiagnosis[b] (PSA level 0.5 ng/ml) (follow-up: 25 years)**											
1[17]	non-randomised studies	very serious[a]	not serious	not serious	not serious	none	10.1%	3.8%	**RR 2.7** (1.8 to 4.5)	**65 more per 1,000** (30 more to 133 more)	⊕◯◯◯ Very low[a]
**Overdiagnosis[b] (PSA level 1.0 ng/ml) (follow-up: 25 years)**											
1[17]	non-randomised studies	very serious[a]	not serious	not serious	not serious	none	16.9%	6.1%	**RR 2.8** (2.3 to 3.5)	**110 more per 1,000** (79 more to 152 more)	⊕◯◯◯ Very low[a]
**Overdiagnosis[b] (PSA level 2.0 ng/ml) (follow-up: 25 years)**											
1[17]	non-randomised studies	very serious[a]	not serious	not serious	not serious	none	21.5%	13.1%	**RR 1.6** (1.5 to 2.0)	**79 more per 1,000** (66 more to 131 more)	⊕◯◯◯ Very low[a]

Certainty assessment							Absolute risk		Effect		Certainty
№ of studies	Study design	Risk of bias	Inconsistency	Indirectness	Imprecision	Other considerations	Biopsy detected cancer in screened cohort (PCPT)	Clinically diagnosed cancer in unscreened cohort (MPP)	Relative (95% CI)	Absolute (95% CI)	
**Overdiagnosis (PSA level 3.0 ng/ml) (follow-up: 25 years)**											
1[17]	non-randomised studies	very serious[a]	not serious	not serious	not serious	none	22.2%	16.8%	**RR 1.3** (1.1 to 1.5)	**50 more per 1,000** (17 more to 84 more)	⊕◯◯◯ Very low[a]
**Overdiagnosis[b] (PSA level 4.0 ng/ml) (follow-up: 25 years)**											
1[17]	non-randomised studies	very serious[a]	not serious	not serious	not serious	none	23.2%	19.6%	**RR 1.2** (0.8 to 1.3)	**39 more per 1,000** (39 fewer to 59 more)	⊕◯◯◯ Very low[a]
**Overdiagnosis[b] (PSA level 5.0 ng/ml) (follow-up: 25 years)**											
1[17]	non-randomised studies	very serious[a]	not serious	not serious	not serious	none	23.5%	21.9%	**RR 1.1** (0.7 to 1.2)	**22 more per 1,000** (66 fewer to 44 more)	⊕◯◯◯ Very low[a]
**Overdiagnosis[b] (PSA level 7.5 ng/ml) (follow-up: 25 years)**											
1[17]	non-randomised studies	very serious[a]	not serious	not serious	not serious	none	24.6%	28.2%	**RR 0.9** (0.4 to 1.1)	**28 fewer per 1,000** (169 fewer to 28 more)	⊕◯◯◯ Very low[a]
**Overdiagnosis[b] (PSA level 10 ng/ml) (follow-up: 25 years)**											
1[17]	non-randomised studies	very serious[a]	not serious	not serious	not serious	none	25.6%	33.6%	**RR 0.8** (0.3 to 1.0)	**67 fewer per 1,000** (235 fewer to 0)	⊕◯◯◯ Very low[a]

CI: confidence interval, PCPT: Prostate Cancer Prevention Trial, MPP: Malmö Preventive Program

Explanations

a. There are concerns with adjusting for appropriate confounding variables (i.e., age, lead time bias, or self-selection bias) and differences in both groups due to the use of two different population cohorts to estimate overdiagnosis in the screened and unscreened groups.

b. Overdiagnosis was estimated by dividing the risk of biopsy detected cancers from the screened PCPT or ERSPC cohorts by the long-term risk of clinical prostate cancer, metastasis and cancer-specific mortality in the unscreened Malmö Preventive Program (MPP) cohort.

Table S8: KQ2 Overdiagnosis (RCTs)

Additional screening test (i.e., risk stratification, MRI, biomarker panels [4K panel, STHLM3 panel], nomograms, etc.) used alone, sequentially or in combination with PSA test to determine the need for biopsy Vs. Usual care (i.e., PSA-based screening only)

Bibliography: Eklund et al., 2021[14]

Certainty assessment							№ of patients		Effect		Certainty
№ of studies	Study design	Risk of bias	Inconsistency	Indirectness	Imprecision	Other considerations	Incorporating additional testing information (i.e., MRI, risk stratification, etc.) with an elevated PSA test	Usual care (PSA-based screening only)	Risk Difference (RD) (95% CI)	Absolute (95% CI)	
**Overdiagnosis[b] (PSA ≥ 3 ng/ml or Stockholm3 ≥ 11%) (follow-up: at least 200 days after receiving PSA results)**											
1[14]	randomised trials	very serious[a]	not serious	not serious	not serious	none	41/929 (4.4%)	73/603 (12.1%)	RD -0.08 (-0.11 to -0.05)	**80 fewer per 1,000** (110 fewer to 50 fewer)	⨁⨁◯◯ Low[a]

RD: Risk difference; MRI: magnetic resonance imaging

Explanations

a. Lack information related to allocation concealment, concerns related to blinding of study participants and carers/clinicians about the assignment of intervention

b. Overdiagnosis was calculated as the percentage difference in the detection of clinically insignificant cancers (Gleason score 6) between the experimental and control groups

Table S9: KQ1 Quality of life (RCTs, cancer specific, UCLA-PCI survey)

Invited to screen vs not invited to screen

Bibliography: Talala et al., 2020[3]

Certainty assessment							№ of patients		Effect		Certainty
№ of studies	Study design	Risk of bias	Inconsistency	Indirectness	Imprecision	Other considerations	screening	no screening	Relative (95% CI)	Absolute (95% CI)	
**Urinary function[b]** (Follow-up: 10 years)											
1[3]	randomised trials	very serious[a]	not serious	not serious	not serious	none	348	223	-	MD **1 points higher** (3 lower to 5 higher)	⨁⨁◯◯ Low[a]
**Urinary bother[b]** (Follow-up: 10 years)											
1[3]	randomised trials	very serious[a]	not serious	not serious	not serious	none	348	223	-	MD **6 points higher** (1 higher to 11 higher)	⨁⨁◯◯ Low[a]
**Bowel function[b]** (Follow-up: 10 years)											
1[3]	randomised trials	very serious[a]	not serious	not serious	not serious	none	348	223	-	MD **1 points higher** (2 lower to 4 higher)	⨁⨁◯◯ Low[a]
**Bowel bother[b]** (Follow-up: 10 years)											
1[3]	randomised trials	very serious[a]	not serious	not serious	not serious	none	348	223	-	MD **4 points higher** (0 to 8 higher)	⨁⨁◯◯ Low[a]

Certainty assessment							№ of patients		Effect		Certainty
№ of studies	Study design	Risk of bias	Inconsistency	Indirectness	Imprecision	Other considerations	screening	no screening	Relative (95% CI)	Absolute (95% CI)	
**Sexual function**[b] (Follow-up: 10 years)											
1[3]	randomised trials	very serious[a]	not serious	not serious	not serious	none	348	223	-	MD **5 points higher** (1 higher to 10 higher)	⊕⊕◯◯ Low[a]
**Sexual bother**[b] (Follow-up: 10 years)											
1[3]	randomised trials	very serious[a]	not serious	not serious	not serious	none	348	223	-	MD **2 points lower** (9 lower to 5 higher)	⊕⊕◯◯ Low[a]

UCLA-PCI: University of California, Los Angeles Prostate Cancer Index, **CI:** confidence interval; **MD:** mean difference

Explanations

a. Blinding and allocation concealment were not possible. Baseline characteristics were not provided for the ERSPC trial, so the effectiveness of the randomization process could not be evaluated. There were very serious concerns with contamination bias and low participation rate in the ERSPC trial, therefore we downgraded twice. Results should also be interpreted with caution due to the overall low response rate, especially for high-risk patients or those on hormonal therapy.
b. Measured using the UCLA-PCI. Scales range from 0 to 100 after re-scoring, with a score of 100 representing optimal health, normal functioning, or no bother.

Table S10: KQ1 Quality of life (RCTs, generic health, RAND-36 survey)

Invited to screen vs not invited to screen

Bibliography: Talala et al., 2020[3]

Certainty assessment							№ of patients		Effect		Certainty
**№ of studies**	**Study design**	**Risk of bias**	**Inconsistency**	**Indirectness**	**Imprecision**	**Other considerations**	**screening**	**no screening**	**Relative (95% CI)**	**Absolute (95% CI)**	
**Physical functioning**[b] (Follow-up: 10 years)											
1[3]	randomised trials	very serious[a]	not serious	not serious	not serious	none	348	223	-	MD **2 points higher** (2 lower to 6 higher)	⊕⊕◯◯ Low[a]
**Role-physical**[b] (Follow-up: 10 years)											
1[3]	randomised trials	very serious[a]	not serious	not serious	not serious	none	348	223	-	MD **2 points lower** (9 lower to 5 higher)	⊕⊕◯◯ Low[a]
**Role-emotional**[b] (Follow-up: 10 years)											
1[3]	randomised trials	very serious[a]	not serious	not serious	not serious	none	348	223	-	MD **1 points higher** (6 lower to 8 higher)	⊕⊕◯◯ Low[a]
**Bodily pain**[b] (Follow-up: 10 years)											

Certainty assessment							№ of patients		Effect		Certainty
№ of studies	Study design	Risk of bias	Inconsistency	Indirectness	Imprecision	Other considerations	screening	no screening	Relative (95% CI)	Absolute (95% CI)	
1[3]	randomised trials	very serious[a]	not serious	not serious	not serious	none	348	223	-	MD **3 points higher** (1 lower to 7 higher)	⊕⊕◯◯ Low[a]
**General health[b]** (Follow-up: 10 years)											
1[3]	randomised trials	very serious[a]	not serious	not serious	not serious	none	348	223	-	MD **3 points higher** (0 to 6 higher)	⊕⊕◯◯ Low[a]
**Vitality[b]** (Follow-up: 10 years)											
1[3]	randomised trials	very serious[a]	not serious	not serious	not serious	none	348	223	-	MD **2 points higher** (2 lower to 6 higher)	⊕⊕◯◯ Low[a]
**Social functioning[b]** (Follow-up: 10 years)											
1[3]	randomised trials	very serious[a]	not serious	not serious	not serious	none	348	223	-	MD **1 points higher** (3 lower to 5 higher)	⊕⊕◯◯ Low[a]
**Mental health[b]** (Follow-up: 10 years)											
1[3]	randomised trials	very serious[a]	not serious	not serious	not serious	none	348	223	-	MD **1 points higher** (2 lower to 4 higher)	⊕⊕◯◯ Low[a]

CI: confidence interval; **MD:** mean difference

Explanations

a. Blinding and allocation concealment were not possible. Baseline characteristics were not provided for the ERSPC trial, so the effectiveness of the randomization process could not be evaluated. There were very serious concerns with contamination bias and low participation rate in the ERSPC trial, therefore we downgraded twice. Results should also be interpreted with caution due to the overall low response rate, especially for high-risk patients or those on hormonal therapy.
b. Measured using the RAND 36-Item Health Survey. Scales range from 0 to 100 after re-scoring, with a score of 100 representing optimal health, normal functioning, or no bother. Scpre means were and 95% CIs were model-adjusted for covariates.

Table S11: KQ2 Adverse events (post-biopsy)

Additional screening test (i.e., risk stratification, MRI, biomarker panels [4K panel, STHLM3 panel], nomograms, etc.) used alone, sequentially or in combination with PSA test to determine the need for biopsy Vs. Usual care (i.e., PSA-based screening only)

Bibliography: Eklund et al., 2021, Nordstrom et al., 2021

Certainty assessment							№ of patients		Effect		Certainty
№ of studies	Study design	Risk of bias	Inconsistency	Indirectness	Imprecision	Other considerations	Incorporating additional testing information (i.e., MRI, risk stratification, etc.) with an elevated PSA test	Usual care (PSA-based screening only)	Risk Difference (RD) (95% CI)	Absolute (95% CI)	
**Post-biopsy infection (PSA ≥ 3 ng/ml or Stockholm3 ≥ 11%) (follow-up: at least 30 days post-biopsy procedure)**											
1	randomised trials	very serious[a]	not serious	not serious	serious[b]	none	20/929 (2.2%)	23/603 (3.8%)	RD -0.02 (-0.04 to 0.001)	**20 fewer per 1,000** (40 fewer to 1 more)	⨁◯◯◯ Very low[a,b]
**Post-biopsy hospitalization (PSA ≥ 3 ng/ml or Stockholm3 ≥ 11%) (follow-up: at least 30 days post-biopsy procedure)**											
1	randomised trials	very serious[a]	not serious	not serious	not serious	none	13/929 (1.4%)	17/603 (2.8%)	RD -0.01 (-0.03 to -0.001)	**10 fewer per 1,000** (30 fewer to 1 fewer)	⨁⨁◯◯ Low[a]
**Post-biopsy infection (PSA <3 & PSA ≥ 3 ng/ml/Stockholm3 ≥ 11%) (follow-up: at least 30 days post-biopsy procedure)**											

Certainty assessment							№ of patients		Effect		Certainty
№ of studies	Study design	Risk of bias	Inconsistency	Indirectness	Imprecision	Other considerations	Incorporating additional testing information (i.e., MRI, risk stratification, etc.) with an elevated PSA test	Usual care (PSA-based screening only)	Risk Difference (RD) (95% CI)	Absolute (95% CI)	
1	randomised trials	very serious[a]	not serious	serious[c]	not serious	none	25/1372 (1.8%)	41/929 (4.5%)	RD -0.03 (-0.04 to -0.01)	**26 fewer per 1,000** (41 fewer to 11 fewer)	⊕◯◯◯ Very low[a,c]
**Post-biopsy hospitalization (PSA <3 & PSA ≥ 3 ng/ml/Stockholm3 ≥ 11%) (follow-up: at least 30 days post-biopsy procedure)**											
1	randomised trials	very serious[a]	not serious	serious[c]	not serious	none	16/1372 (1.2%)	31/921 (3.4%)	RD -0.02 (-0.03 to -0.01)	**22 fewer per 1,000** (34 fewer to 9 fewer)	⊕◯◯◯ Very low[a,c]

RD: Risk difference; MRI: magnetic resonance imaging

Explanations

a. Lack information related to allocation concealment, concerns related to blinding of study participants and carers/clinicians about the assignment of intervention

b. Rated down once for imprecision. The OIS criterion is not met (n=1532, i.e., <2000 patients) suggesting that the study itself has low power in making the effect measure to cross the null. Additionally, the CI is very tight.

c. Rated down once for indirectness. Participants in the Nordstrom et al. were drawn from the STHLM3-MRI trial population (same as Eklund et al.); however, the Nordstrom study population appears to be potentially contaminated, including individuals with PSA ≥ 3 ng/ml, PSA < 3 ng/ml, or a Stockholm3 score ≥ 11%, which may not fully align with the intended target population (i.e., PSA≥ 3 ng/ml).

Bibliography

1. Martin, R.M.; Turner, E.L.; Young, G.J.; Metcalfe, C.; Walsh, E.I.; Lane, J.A.; Sterne, J.A.C.; Noble, S.; Holding, P.; Ben-Shlomo, Y.; et al. Prostate-Specific Antigen Screening and 15-Year Prostate Cancer Mortality: A Secondary Analysis of the CAP Randomized Clinical Trial. *JAMA* **2024**, *331*, 1460–1470, doi:10.1001/jama.2024.4011.
2. Walter, S.D.; Hu, J.; Talala, K.; Tammela, T.; Taari, K.; Auvinen, A. Estimating the Rate of Overdiagnosis with Prostate Cancer Screening: Evidence from the Finnish Component of the European Randomized Study of Screening for Prostate Cancer. *Cancer Causes Control 32*, 1299–1313, doi:10.1007/s10552-021-01480-8.
3. Talala, K.; Heinävaara, S.; Taari, K.; Tammela, T.L.J.; Kujala, P.; Stenman, U.; Malila, N.; Auvinen, A. Long-term Health-related Quality of Life among Men with Prostate Cancer in the Finnish Randomized Study of Screening for Prostate Cancer. *Cancer Medicine* **2020**, *9*, 5643–5654, doi:10.1002/cam4.3181.
4. Lindberg, A.; Talala, K.; Kujala, P.; Stenman, U.H.; Taari, K.; Kilpelainen, T.P.; Tammela, T.L.; Auvinen, A. Bias-Corrected Estimates of Effects of PSA Screening Decisions on the Risk of Prostate Cancer Diagnosis and Death: Analysis of the Finnish Randomized Study of Screening for Prostate Cancer. *International journal of cancer* **2019**, *Vol.145*, 632–638p, doi:10.1002/ijc.32129.
5. Pakarainen, T.; Nevalainen, J.; Talala, K.; Taari, K.; Raitanen, J.; Kujala, P.; Stenman, U.H.; Tammela, T.L.J.; Auvinen, A. The Number of Screening Cycles Needed to Reduce Prostate Cancer Mortality in the Finnish Section of the European Randomized Study of Prostate Cancer (ERSPC). *Clinical cancer research Vol.25*, 839–843p, doi:10.1158/1078-0432.CCR-18-1807.
6. Villers, A.; Bessaoud, F.; Trétarre, B.; Grosclaude, P.; Malavaud, B.; Rébillard, X.; Iborra, F.; Daubisse, L.; Malavaud, S.; Roobol, M. Contamination in Control Group Led to No Effect of PSA-Based Screening on Prostate Cancer Mortality at 9 Years Follow-up: Results of the French Section of European Randomized Study of Screening for Prostate Cancer (ERSPC). *Progrès en Urologie* **2020**, *30*, 252–260.
7. Osses, D.F.; Remmers, S.; Schroder, F.H.; van der Kwast, T.; Roobol, M.J. Results of Prostate Cancer Screening in a Unique Cohort at 19 Yr of Follow-Up. *European Urology Vol.75*, 374–377p, doi:10.1016/j.eururo.2018.10.053.
8. Hogenhout, R.; Remmers, S.; van Slooten-Midderigh, M.E.; e Vos, I.I.; Roobol, M.J. From Screening to Mortality Reduction: An Overview of Empirical Data on the Patient Journey in European Randomized Study of Screening for Prostate Cancer Rotterdam After 21 Years of Follow-up and a Reflection on Quality of Life. *European urology oncology* **2024**, *7*, 713–720, doi:10.1016/j.euo.2023.08.011.
9. Pasanen, N.; Talala, K.; Remmers, S.; Tammela, T.L.J.; Hugosson, J.; Roobol, M.J.; Taari, K.; Arnsrud Godtman, R.; Bangma, C.; Auvinen, A. Which Men Benefit from Prostate Cancer Screening? Prostate Cancer Mortality by Subgroup in the European Randomised Study of Screening for Prostate Cancer. *BJU international* **2024**, *134*, 291–299, doi:10.1111/bju.16394.
10. Lujan Galan, M.; Paez Borda, A.; Llanes Gonzalez, L.; Romero Cajigal, I.; Berenguer Sanchez, A. Results of the Spanish Section of the European Randomized Study of Screening for Prostate Cancer (ERSPC). Update after 21 Years of Follow-Up. *Actas Urol Esp (Engl Ed) 44*, 430–436, doi:10.1016/j.acuro.2020.01.005.
11. Franlund, M.; Mansson, M.; Godtman, R.A.; Aus, G.; Holmberg, E.; Kollberg, K.S.; Lodding, P.; Pihl, C.G.; Stranne, J.; Lilja, H.; et al. Results from 22 Years of Followup in the Goteborg Randomized Population-Based Prostate Cancer Screening Trial. *J Urol* 101097JU0000000000002696, doi:10.1097/JU.0000000000002696.
12. Pinsky, P.F.; Miller, E.; Prorok, P.; Grubb, R.; Crawford, E.D.; Andriole, G. Extended Follow-up for Prostate Cancer Incidence and Mortality among Participants in the Prostate, Lung, Colorectal and Ovarian Randomized Cancer Screening Trial. *BJU Int* **2019**, *123*, 854–860, doi:10.1111/bju.14580.
13. Nordstrom, T.; Discacciati, A.; Bergman, M.; Clements, M.; Aly, M.; Annerstedt, M.; Glaessgen, A.; Carlsson, S.; Jaderling, F.; Eklund, M.; et al. Prostate Cancer Screening Using a Combination of Risk-Prediction, MRI, and Targeted Prostate Biopsies (STHLM3-MRI): A Prospective, Population-Based, Randomised, Open-Label, Non-Inferiority Trial. *Lancet Oncol* **2021**, *22*, 1240–1249, doi:10.1016/S1470-2045(21)00348-X.
14. Eklund, M.; Jäderling, F.; Discacciati, A.; Bergman, M.; Annerstedt, M.; Aly, M.; Glaessgen, A.; Carlsson, S.; Grönberg, H.; Nordström, T. MRI-Targeted or Standard Biopsy in Prostate Cancer Screening. *N Engl J Med* **2021**, *385*, 908–920, doi:10.1056/nejmoa2100852.
15. Hugosson, J.; Roobol, M.J.; Månsson, M.; Tammela, T.L.J.; Zappa, M.; Nelen, V.; Kwiatkowski, M.; Lujan, M.; Carlsson, S.V.; Talala, K.M.; et al. A 16-Yr Follow-up of the European Randomized Study of Screening for Prostate Cancer. *Eur Urol* **2019**, *76*, 43–51, doi:10.1016/j.eururo.2019.02.009.
16. de Vos, I.I.; Meertens, A.; Hogenhout, R.; Remmers, S.; Roobol, M.J. A Detailed Evaluation of the Effect of Prostate-Specific Antigen–Based Screening on Morbidity and Mortality of Prostate Cancer: 21-Year Follow-up Results of the Rotterdam Section of the European Randomised Study of Screening for Prostate Cancer. *European Urology* **2023**, *84*, 426–434, doi:10.1016/j.eururo.2023.03.016.
17. Vickers, A.J.; Sjoberg, D.D.; Ulmert, D.; Vertosick, E.; Roobol, M.J.; Thompson, I.; Heijnsdijk, E.A.; De Koning, H.; Atoria-Swartz, C.; Scardino, P.T.; et al. Empirical Estimates of Prostate Cancer Overdiagnosis by Age and Prostate-Specific Antigen. *BMC medicine* **2014**, *12*, doi:10.1186/1741-7015-12-26.

Supplementary File S10: Sensitivity Analyses

Figure S1. RCT - Prostate cancer mortality, all ages (low RoB RCTs)

Study or Subgroup	Screening Events	Screening Total	No screening Events	No screening Total	Weight	Risk Ratio M-H, Random, 95% CI
Martin 2024 (CAP)	1199	189326	1451	219395	32.4%	0.96 [0.89, 1.03]
Hugosson 2019 (ERSPC Multiple)	520	72890	793	89351	28.7%	0.80 [0.72, 0.90]
Franlund 2022 (ERSPC Sweden)	112	9945	158	9949	15.6%	0.71 [0.56, 0.90]
de Vos 2023 (ERSPC Netherlands)	283	20984	341	20916	23.3%	0.83 [0.71, 0.97]
**Total (95% CI)**		**293145**		**339611**	**100.0%**	**0.84 [0.74, 0.95]**
Total events	2114		2743			

Heterogeneity: Tau² = 0.01; Chi² = 11.00, df = 3 (P = 0.01); I² = 73%
Test for overall effect: Z = 2.74 (P = 0.006)

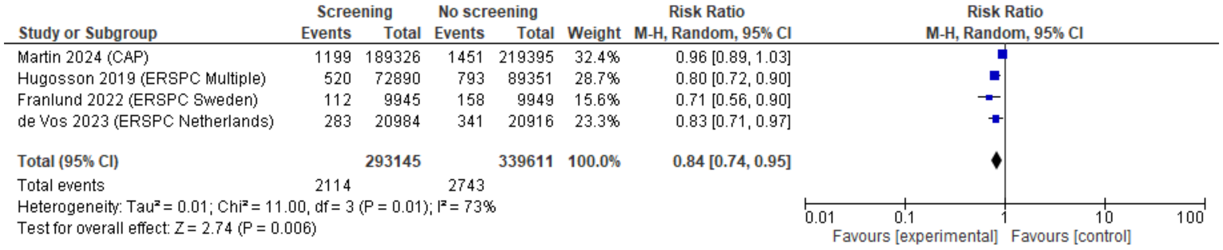

Figure S2. RCT - Prostate cancer mortality, all ages (without Hugosson et al., 2019)

Study or Subgroup	Screening Events	Screening Total	No screening Events	No screening Total	Weight	Risk Ratio M-H, Random, 95% CI
de Vos 2023 (ERSPC Netherlands)	283	20984	341	20916	17.2%	0.83 [0.71, 0.97]
Franlund 2022 (ERSPC Sweden)	112	9945	158	9949	9.2%	0.71 [0.56, 0.90]
Martin 2024 (CAP)	1199	189326	1451	219395	33.3%	0.96 [0.89, 1.03]
Pakaraine 2019 (ERSPC Finland)	229	31867	383	48282	16.3%	0.91 [0.77, 1.07]
Pinsky 2019 (PLCO)	333	38340	352	38343	18.2%	0.95 [0.81, 1.10]
Villers 2020 (ERSPC France)	78	38474	75	38161	5.8%	1.03 [0.75, 1.42]
**Total (95% CI)**		**328936**		**375046**	**100.0%**	**0.90 [0.83, 0.98]**
Total events	2234		2760			

Heterogeneity: Tau² = 0.00; Chi² = 8.00, df = 5 (P = 0.16); I² = 38%
Test for overall effect: Z = 2.48 (P = 0.01)

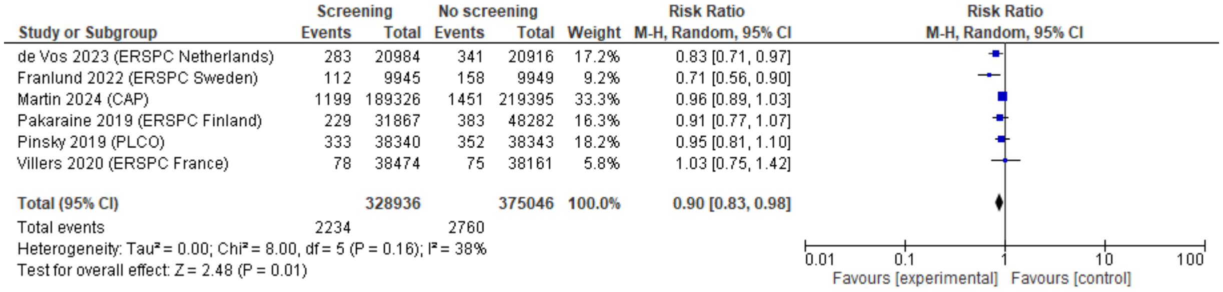

Figure S3. Prostate cancer mortality, 50-59 years (low RoB RCTs)

Study or Subgroup	Screening Events	Screening Total	No screening Events	No screening Total	Weight	Risk Ratio M-H, Random, 95% CI
Pasanen 2024 (ERSPC Netherlands)	31	6827	43	6663	54.5%	0.70 [0.44, 1.12]
Pasanen 2024 (ERSPC Sweden)	18	3123	43	3162	45.5%	0.42 [0.25, 0.73]
**Total (95% CI)**		**9950**		**9825**	**100.0%**	**0.56 [0.34, 0.92]**
Total events	49		86			

Heterogeneity: Tau² = 0.06; Chi² = 1.93, df = 1 (P = 0.16); I² = 48%
Test for overall effect: Z = 2.31 (P = 0.02)

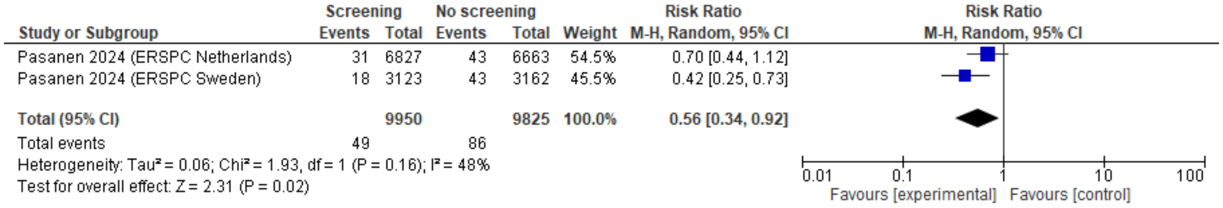

Figure S4. Prostate cancer mortality, 60-69 years (low RoB RCTs)

Study or Subgroup	Screening Events	Screening Total	No screening Events	No screening Total	Weight	Risk Ratio M-H, Random, 95% CI
Pasanen 2024 (ERSPC Netherlands)	79	10615	123	10727	57.2%	0.65 [0.49, 0.86]
Pasanen 2024 (ERSPC Sweden)	43	2777	44	2789	42.8%	0.98 [0.65, 1.49]
**Total (95% CI)**		**13392**		**13516**	**100.0%**	**0.77 [0.52, 1.16]**
Total events	122		167			

Heterogeneity: Tau² = 0.05; Chi² = 2.60, df = 1 (P = 0.11); I² = 61%
Test for overall effect: Z = 1.25 (P = 0.21)

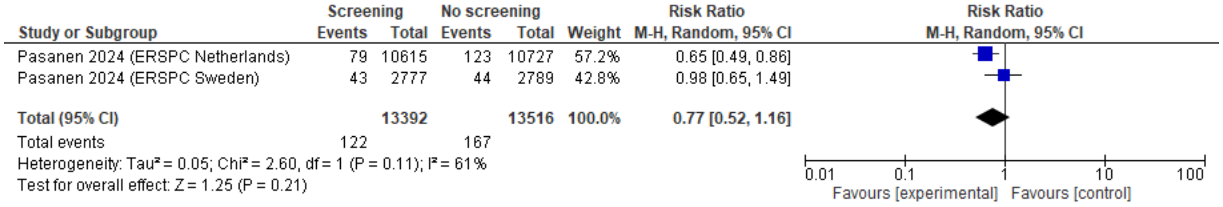

Figure S5. All-cause mortality, all ages (low RoB RCTs)

Study or Subgroup	Experimental Events	Experimental Total	Control Events	Control Total	Weight	Risk Ratio M-H, Random, 95% CI
Franlund 2022 (ERSPC Sweden)	3806	9945	3735	9949	26.2%	1.02 [0.98, 1.06]
Hugosson 2019 (ERSPC Multiple)	20352	72890	25365	89351	31.9%	0.98 [0.97, 1.00]
Lujan Galan 2020 (ERSPC Spain)	544	2415	450	1861	9.1%	0.93 [0.84, 1.04]
Martin 2024 (CAP)	45084	189326	50336	219395	32.8%	1.04 [1.03, 1.05]
**Total (95% CI)**		**274576**		**320556**	**100.0%**	**1.01 [0.97, 1.05]**
Total events	69786		79886			

Heterogeneity: Tau² = 0.00; Chi² = 32.79, df = 3 (P < 0.00001); I² = 91%
Test for overall effect: Z = 0.28 (P = 0.78)

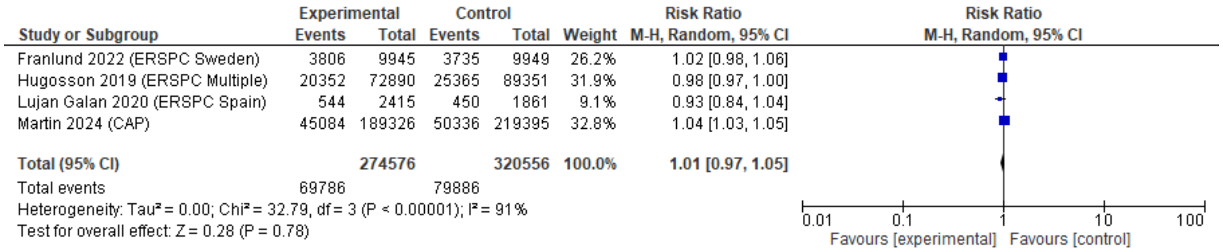

Figure S6. All-cause mortality, all ages (without Hugosson et al., 2019)

Study or Subgroup	Experimental Events	Experimental Total	Control Events	Control Total	Weight	Risk Ratio M-H, Fixed, 95% CI
Franlund 2022 (ERSPC Sweden)	3806	9945	3735	9949	6.7%	1.02 [0.98, 1.06]
Lujan Galan 2020 (ERSPC Spain)	544	2415	450	1861	0.9%	0.93 [0.84, 1.04]
Martin 2024 (CAP)	45084	189326	50336	219395	83.4%	1.04 [1.03, 1.05]
Villers 2020 (ERSPC France)	5249	38474	5028	38161	9.0%	1.04 [1.00, 1.07]
**Total (95% CI)**		**240160**		**269366**	**100.0%**	**1.04 [1.03, 1.05]**
Total events	54683		59549			

Heterogeneity: Chi² = 4.51, df = 3 (P = 0.21); I² = 33%
Test for overall effect: Z = 6.73 (P < 0.00001)

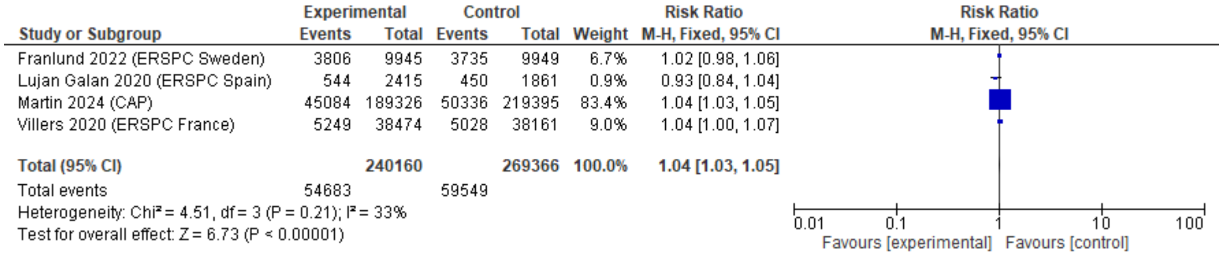

Figure S7. All-cause mortality, 60-69 years (low RoB studies)

Study or Subgroup	Experimental Events	Experimental Total	Control Events	Control Total	Weight	Risk Ratio M-H, Random, 95% CI
Hugosson 2019 (ERSPC Multiple)	20352	72953	25365	89436	100.0%	0.98 [0.97, 1.00]
**Total (95% CI)**		**72953**		**89436**	**100.0%**	**0.98 [0.97, 1.00]**
Total events	20352		25365			

Heterogeneity: Not applicable
Test for overall effect: Z = 2.07 (P = 0.04)

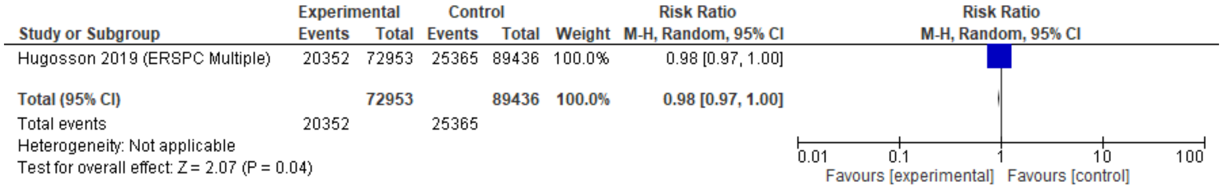

Figure S8. All-cause mortality, 60-69 years (without Hugosson et al., 2019)

Study or Subgroup	Experimental Events	Experimental Total	Control Events	Control Total	Weight	Risk Ratio M-H, Random, 95% CI
Villers 2020 (ERSPC France)	3866	24784	3710	24445	100.0%	1.03 [0.99, 1.07]
**Total (95% CI)**		**24784**		**24445**	**100.0%**	**1.03 [0.99, 1.07]**
Total events	3866		3710			

Heterogeneity: Not applicable
Test for overall effect: Z = 1.30 (P = 0.19)

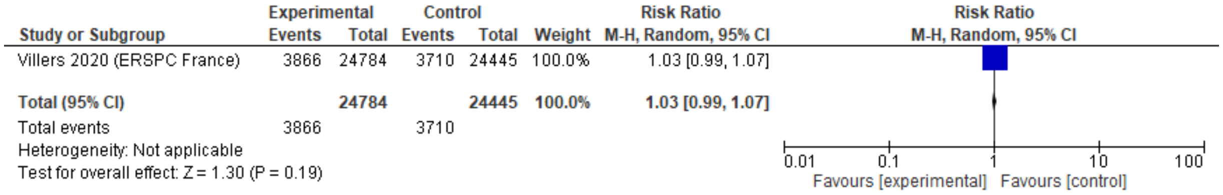

Figure S9. Metastatic cancer, all ages (low RoB RCTs)

Study or Subgroup	Screening Events	Screening Total	Control Events	Control Total	Weight	Risk Ratio M-H, Random, 95% CI
Lujan Galan 2020 (ERSPC Spain)	7	188	5	97	38.1%	0.72 [0.24, 2.22]
Ossess 2018 (ERSPC Netherlands)	7	553	16	581	61.9%	0.46 [0.19, 1.11]
**Total (95% CI)**		**741**		**678**	**100.0%**	**0.55 [0.27, 1.09]**
Total events	14		21			

Heterogeneity: Tau² = 0.00; Chi² = 0.39, df = 1 (P = 0.53); I² = 0%
Test for overall effect: Z = 1.71 (P = 0.09)

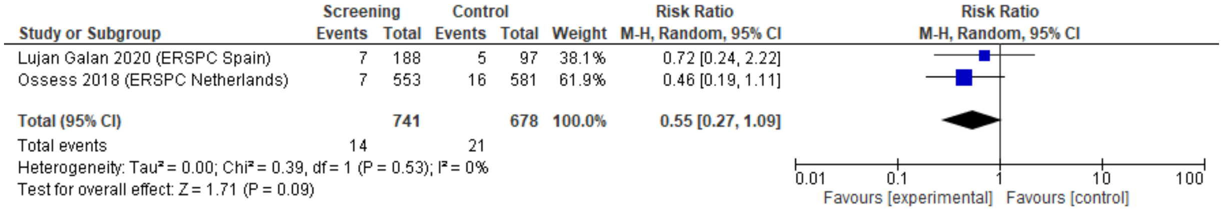

Supplement: Supplementary file 1 [file curroncol-33-00199-s001.zip › curroncol-4167371-supplementary.pdf]
